# Regional diversity of cranial skeletal stem cells governs bone morphogenetic protein-2 mediated bone regeneration

**DOI:** 10.1038/s41467-026-76286-w

**Published:** 2026-08-06

**Authors:** Eri Takematsu, Rovin N. Lachmansingh, Kyle Johnston, Sophia Hou, Elyon Bailey, Yuting Wang, Liming Zhao, Malachia Hoover, Hieu T. M. Nguyen, Kumarizeel M. Parmar, Heather Talbott, Michael T. Longaker, Charles K. F. Chan

**Affiliations:** 1 Division of Plastic and Reconstructive Surgery, Department of Surgery, Stanford University School of Medicine, Stanford, CA USA; 2 Department of Periodontics and Oral Medicine, School of Dentistry, University of Michigan, Ann Arbor, MI USA; 3 Molecular and Cellular Biology, University of Illinois at Urbana-Champaign, Urbana, IL USA; 4 Department of Neurobiology, Physiology and Behavior, University of California at Davis, Davis, CA USA; 5 Department of Radiation Oncology and Medical Physics, School of Medicine, Stanford University, Palo Alto, CA USA

**Keywords:** Regeneration, Adult stem cells, Bone

## Abstract

Skeletal stem cells (SSCs), originally identified in 2015, are increasingly understood to exhibit significant heterogeneity throughout the body. Cranial SSCs provide a unique model to investigate this diversity because cranial bones arise from both neural crest and mesoderm, unlike mesoderm-derived long bones. Here, we show that cranial SSCs possess anatomically defined transcriptomic and functional heterogeneity. Through comprehensive in vitro and in vivo functional assays combined with targeted gene expression analyses by qPCR, we demonstrate that cranial SSCs exhibit region-specific transcriptional signatures and lineage biases that influence their osteogenic and chondrogenic capacities. Temporal analysis further reveals that SSCs progressively outnumber progenitor cells with age, suggesting that the SSC-to-progenitor ratio may serve as an indicator of skeletal developmental and regenerative potential. Finally, we demonstrate that SSC heterogeneity critically influences bone regeneration in response to Bone Morphogenetic Protein-2 in vitro and in vivo, supporting the need for region-specific optimization of BMP-2-based regenerative therapies.

## Abstract (web summary)

Here the authors show that cranial skeletal stem cells differ depending on where they are located, with distinct gene activity and regenerative potential. Their responses to the regenerative protein BMP-2 also vary, suggesting that future bone repair treatments may need to be tailored.

## Introduction

Adult stem cells are pivotal in regenerative medicine due to their self-renewal capabilities and multi-potency, which allows them to differentiate into distinct cell types of their specific lineage^1^. Mesenchymal stem cells (MSCs) have traditionally been regarded as the progenitors for skeletal tissues^2^. However, MSCs are heterogenous and possess the capacity to differentiate into more than one lineage, including skeletal, adipogenic, and muscle tissues. This multi-lineage potential renders MSCs suboptimal for the targeted regeneration of skeletal tissues alone^3^. In 2015, our laboratory identified skeletal stem cells (SSCs) that exclusively give rise to skeletal cell fates: bone, cartilage, and stromal cells^4^. This discovery spurred subsequent, extensive characterization of SSCs in physiologic and pathologic conditions. These studies primarily focused on SSCs from endochondral-derived long bones, such as the femur^5–7^. For instance, SSCs in the knee were found to be capable of regenerating articular cartilage following microfracture surgery, thus presenting a new avenue for treating osteoarthritis^8^. Additionally, aged SSCs were shown to regain youthful levels of activity via combination therapy with a CSF1 antagonist and BMP-2, suggesting a novel approach to target impaired fracture healing in elderly individuals^9^.

While a growing body of research has explored femoral SSCs, the study of cranial bone-derived SSCs has been limited. Given that cranial and long bones have different developmental origins (the femur is derived from lateral plate mesoderm, while cranial bones originate from the neural crest and paraxial mesoderm), it stands to reason that they may also possess distinct molecular profiles and functional capacities. Such divergence would be consistent with growing evidence suggesting functional stem cell diversity in different regions of the skeleton^10,11^.

In this study, we identified the anatomical location of stem cells within cranial bones via clonality tracing with Actin^CreER^;R26^VT2/GK3^ mice, followed by comprehensive in vitro and in vivo functional and gene expression analyses of the immunophenotypically isolated SSCs. Our gene expression analysis of cranial SSCs isolated from different regions of the skull revealed distinct patterns of osteogenic and chondrogenic gene regulation, consistent with functional assays demonstrating region-specific differences in lineage commitment. Finally, by tracking SSC and progenitor dynamics across developmental stages, we introduce the BCSP/SSC ratio (bone cartilage stromal progenitors/skeletal stem cell ratio) as a quantitative indicator of skeletal tissue activity, providing a framework to assess both developmental growth and regenerative potential in different bones.

Understanding the functional and transcriptional diversity of SSCs is critically important for the success of bone regenerative therapies. Current craniofacial bone regenerative therapy relies on autografts from the iliac crest^12^. However, autografting poses concerns for both potential graft failure and donor site morbidity^13,14^, underscoring the demand for alternative, less invasive treatment modalities. One promising approach for regenerating cranial bones without autografts involves growth factor-based bone regenerative therapies, such as using recombinant bone morphogenetic protein-2 (BMP-2) to induce the production of bone-forming cells from native stem cells^15,16^. However, this approach was originally developed in the context of femoral regeneration, and the treatment regimen has therefore been tailored to long bone repair. An analogous BMP-2 dosage has yet to be established for cranial bone regeneration, and given the biological differences between these skeletal regions, the optimal dosing for cranial bone is likely distinct from that used for long bones. Lack of dose standardization may underlie the varied outcomes of clinical trials for BMP-2-based cranial bone regeneration depending on anatomical site. For example, reported success rates of BMP-2 for promoting bone healing have been as low as 50% in the palate or maxillary bone, compared to over 90% in the mandible^17–20^. Certainly, many factors could contribute to these disparate outcomes—for instance, differences in bone density^21,22^ and mechanical forces^19,23^. However, given the central role of stem cells in bone regeneration^8,24^ and our findings of anatomical heterogeneity of cranial SSC phenotypes, we hypothesized that variability in treatment outcomes is attributable to SSCs’ differential responsiveness to BMP signaling. Mounting evidence has linked differing SSC transcriptomic and functional profiles—which could underlie differential BMP responsiveness and healing potential—to the anatomical site of origin^11,25,26^. While SSC heterogeneity is not a new concept, its functional relevance to BMP-dependent bone repair was previously undefined. Our study bridges this gap by demonstrating that cranial SSCs from different anatomical origins interpret BMP-2 stimulation through distinct lineage programs, uncovering a positional code that governs regenerative behavior. These findings reframe the concept of SSC diversity as a directly clinically relevant determinant of therapeutic response, thus paving the way for precision-targeted BMP treatment for cranial bone regeneration.

## Results

### Clonality tracing with Actin^CreER^;R26^VT2/GK3^ mice demonstrated stem-like cell populations in cranial bones

**Fig. 1:**
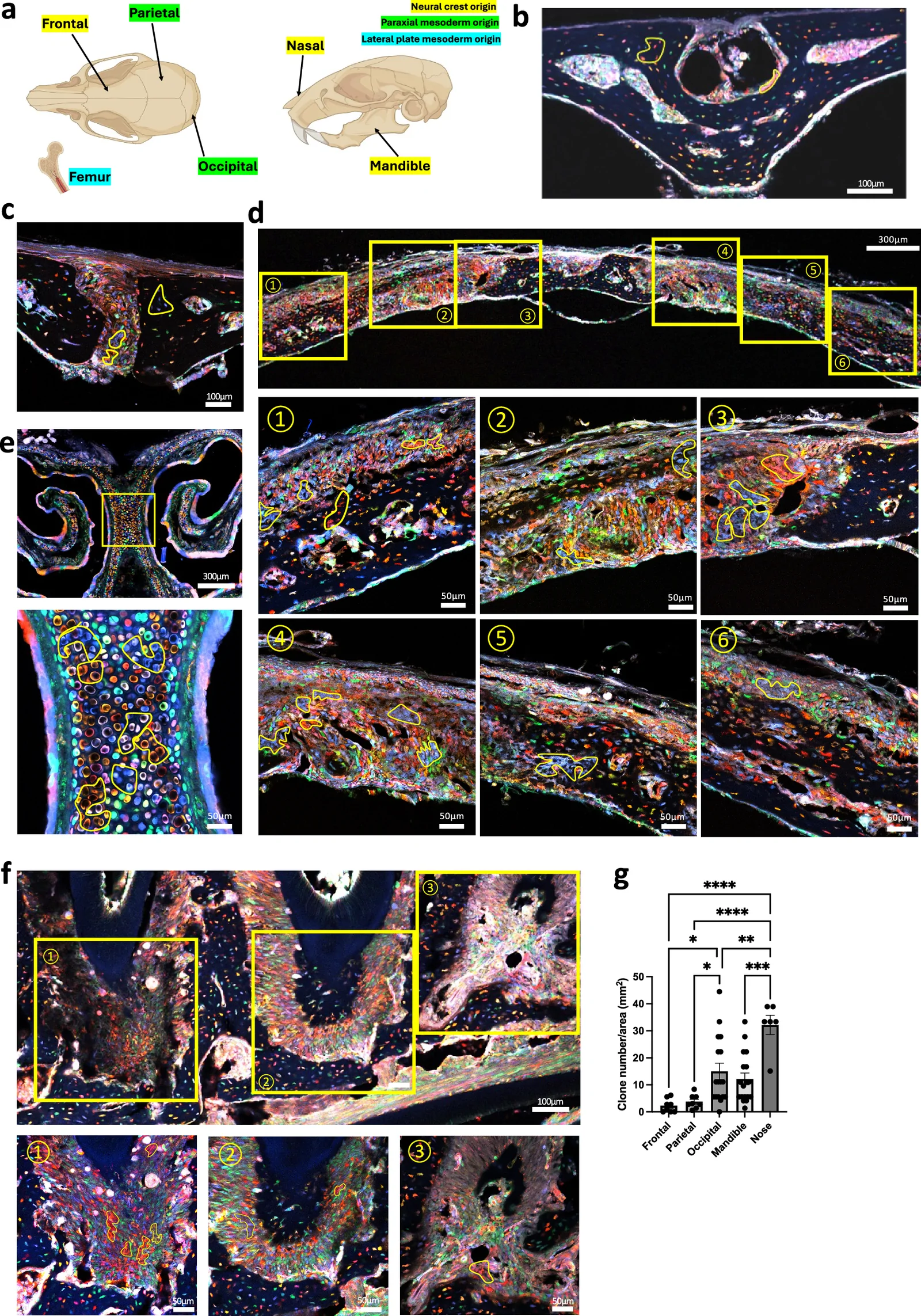
Lineage tracing with Actin^CreER^;R26^VT2/GK3^ mice revealed clonally expanded cells in cranial bones. **a** Schematic illustration of mouse bones evaluated in this study, indicating their developmental origin. Created in BioRender. Takematsu, E. (2026) https://BioRender.com/mftr3ke. Confocal images of the frontal bone (**b**), parietal bone (**c**), occipital bone (**d**), nasal bone (**e**), and mandibular bone (**f**) from Actin^CreER^;R26^VT2/GK3^ mice, with yellow outlined regions indicating clones composed of ≥5 adjacent cells expressing the same Rainbow color. All images were taken at postnatal day (P)45, 35 days after tamoxifen injection at P10. **b**–**e** Is coronal, and **f** is sagittal section. **d–f** Show low magnification image in top panel, with higher magnification (20×) images in bottom panels (from regions corresponding to yellow squares in lower-power images). **g** Quantification of clone density (clones per mm^2^) in the indicated bones. *n* = 4–6 mice; multiple fields were analyzed per region and per mouse. Statistical significance was determined by one-way ANOVA with Tukey’s post hoc test. For indicated comparisons, *p < 0.05, **p < 0.01, ***p < 0.001, and ****p < 0.0001. Statistical analyses were performed using GraphPad Prism 9 (GraphPad).

In order to identify the location of SSCs within the cranium, we utilized the Actin^CreER^;R26^VT2/GK3^ mouse to assess clonal skeletogenesis in the frontal, parietal, occipital, and nasal bones and the mandible (Fig. 1a) as described previously^8^. The Actin^CreER^;R26^VT2/GK3^ system enables assessment of clonal dynamics without restricting analysis to a predefined lineage. Thus, labeled cells may include SSCs and/or other progenitor populations. This approach allows comprehensive evaluation of cellular contributions within the tissue, which would not be achievable using lineage-specific drivers. To systemically induce recombination of the rainbow construct (leading to random colorimetric labeling of all cells), we delivered tamoxifen via oral gavage into postnatal day 10 (P10) mice. We then isolated cranial tissue at P45 and measured the frequency of cellular clones in tissues by histological analysis of identically-colored cell clusters (indicating that the cells all originated from the same parent cell). One month after the initial tamoxifen pulse is the standardized time point for analyzing clonality to indicate the presence of progenitor cells^4,8,27–29^. We counted a cluster consisting of five or more same-colored cells as one clone, and measured the location and frequency of these clones, which would indicate potential stem cells (*n* = 3–6 mice).

In the frontal bone, clones were mainly found at the posterior-frontal suture and occasionally in mineralized bone areas (Fig. 1b, yellow circles). Similarly, in the parietal bone, clones were primarily located at the sagittal suture and sometimes in mineralized bone areas (Fig. 1c). In the occipital bone, numerous clones were observed within the suture mesenchyme (Fig. 1d). In the nasal bone, clones were mainly located in the cartilaginous area (Fig. 1e). In the mandibular bone, clones were predominantly located in the area of the periodontal ligament (PDL) (Fig. 1f). Quantification of clone density in each bone revealed that clones were most abundant in the nasal bone, followed by the occipital, mandibular, parietal, and frontal bones (Fig. 1g).

To further interrogate whether the observed clones in fact represented long-lasting stem cells, we examined clonality 12 weeks after tamoxifen induction, corresponding to the P13.5-week time point (Supplementary Fig. 1a–e). Similar to the P45 time point, the highest clonality at P13.5-weeks was observed in the nasal bone, followed by the occipital, mandibular, and parietal bones (Supplementary Fig. 1f). Interestingly, no clones were detected in the frontal bone at this later time point (Supplementary Fig. 1a). This finding was supported by quantitative comparison of clone density between P45 and P13.5-weeks (Supplementary Fig. 2a), suggesting that the frontal bone cells found to give rise to clones at P10 are likely progenitor cells rather than long-lasting SSCs. The transient nature of clonal contribution suggests that these cells lack long-term self-renewal, a defining feature of skeletal stem cells. In contrast to this finding in the frontal bone, clone density in the parietal, occipital, mandibular, and nasal bones remained similar between P45 and P13.5-weeks (Supplementary Fig. 2b–e), indicating that labeled cells in these regions are in fact long-term stem cells. Supplementary Figs. 3 and 4 show the corresponding hematoxylin and eosin (H&E) staining for the P45 and P13.5-weeks time points.

### Skeletal stem cells from cranial bones demonstrate functional variability in vitro and in vivo

**Fig. 2:**
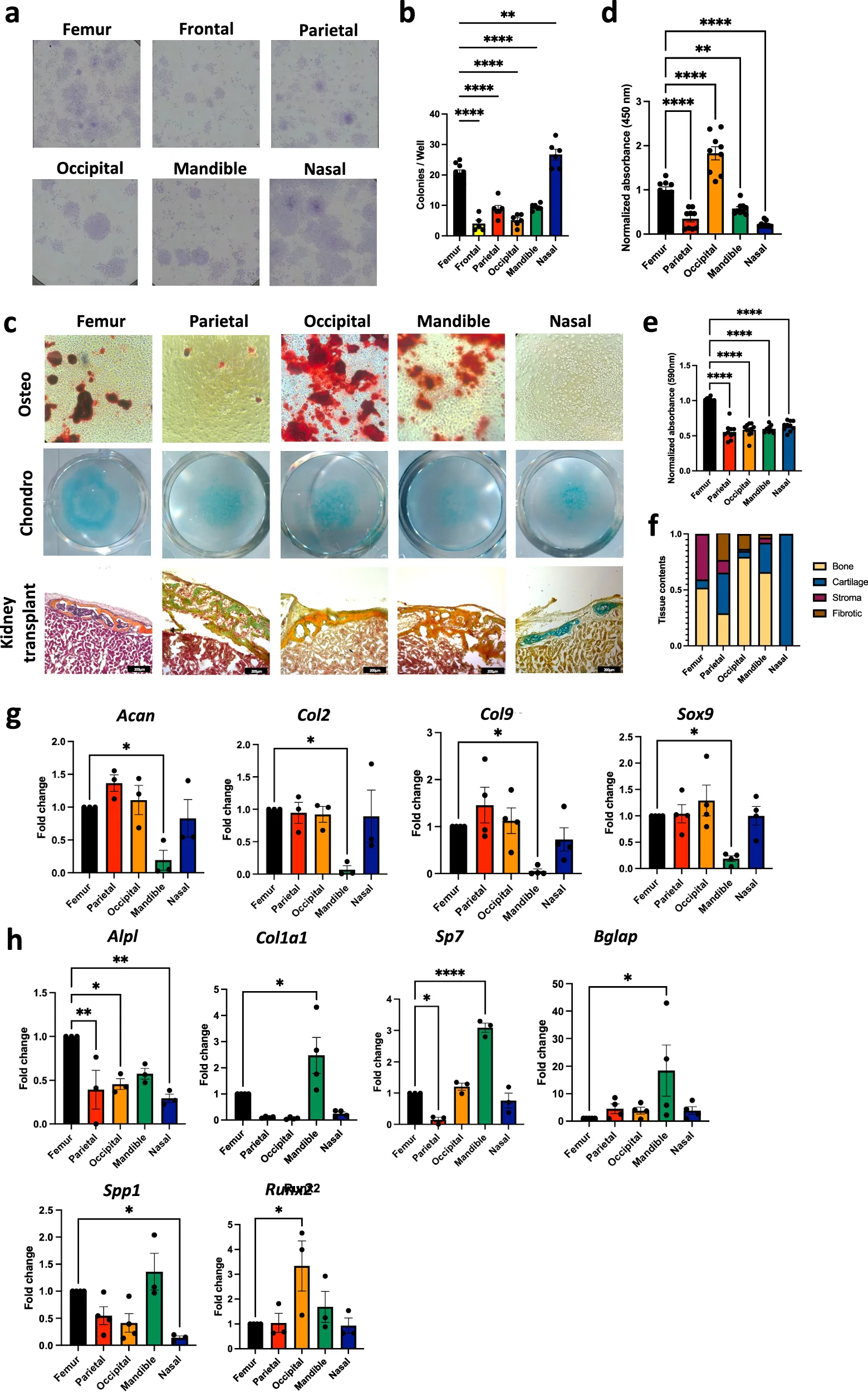
In vitro and in vivo assays revealed functional diversity of cranial-SSCs based on their anatomical location. **a** Representative crystal violet staining images of colony forming unit (CFU) assays for SSCs from indicated bones. **b** Quantification of CFU assay results showing number of colonies per well for each bone (*n* = 2–3 biological replicates, with 5–9 technical replicates each, per bone type). **c** Top row: representative Alizarin Red staining images for osteogenic assay of SSCs from indicated bones. Middle row: representative Alcian Blue staining images for chondrogenic assay of SSCs from indicated bones. Bottom row: representative Pentachrome staining of recipient kidneys 4 weeks following renal transplant of SSCs (1 × 10^5^ SSCs isolated from P3 mice per implantation) from the indicated bones. Cartilage, green; bone, yellow/orange. **d** Quantification results of Alizarin Red staining following osteogenesis assay (*n* = 3 biological replicates/9 technical replicates total per condition). **e** Quantification results of Alcian Blue staining following chondrogenesis assay (*n* = 3 biological replicates/7–9 technical replicates total per condition). **f** Quantification of the proportion of different tissue types arising from renal transplant of SSCs. Relative areas of bone, cartilage, stroma, and fibrotic tissue were quantified from Pentachrome staining images using Fiji imaging software (results are the average of *n* = 3 grafts/biological replicates per group). Quantification of relative expression levels of **g** chondrogenic genes *Acan, Col2, Col9, and Sox9*; and **h** osteogenic genes *Alpl, Col1, Sp7, Bglap, Spp1, and Runx2;* from qPCR analysis of freshly isolated SSCs from indicated bones. Gene expression levels are normalized to those of femur-SSCs from each experimental replicate. Data are presented as mean ± SEM from *n* = 3–4 biological replicates. **b**, **d**, **e**–**h** Statistical significance was determined by one-way ANOVA with a Tukey post-hoc test; for indicated comparisons, *p < 0.05, **p < 0.01, ***p < 0.001, and ****p < 0.0001.

In order to evaluate the intrinsic functional ability of SSCs residing in each cranial region, we dissected the cranial bones from P3 mice pups and prospectively isolated SSCs from these bones using fluorescence-activated cell sorting (FACS) with surface markers established previously^4^ (FACS gating strategy demonstrated in Supplementary Fig. 5). We also isolated P3 femoral SSCs in the same manner for comparison. We first conducted an in vitro colony-forming assay to evaluate clonogenic potential of SSCs from each region of the cranium (Fig. 2a). Upon plating, nasal bone-derived SSCs exhibited the highest colony-forming ability, followed by femoral, mandibular, parietal, occipital, and frontal SSCs (Fig. 2b). The enhanced colony-forming activity observed in nasal SSCs corresponds to greater clonal expansion in this bone in vivo, while the low colony-forming ability of frontal SSCs is consistent with limited frontal bone clonality observed in vivo (Fig. 1g). Collectively, these findings support that in vitro colony-forming potential does reflect the in vivo abundance and self-renewal capacity of SSCs within each region.

Next, in order to compare SSC differentiation potential between the different cranial bones, we conducted in vitro osteogenesis and chondrogenesis assays (Fig. 2c). The frontal bone was omitted from these experiments, as we were unable to obtain sufficient SSCs from the frontal region. SSCs from all cranial regions other than the nasal bone demonstrated osteogenic ability; quantification revealed that the osteogenic ability of occipital-SSCs was highest, followed by femur-, mandibular-, parietal-, and nasal-SSCs (Fig. 2d). Chondrogenesis assays revealed cartilage-forming activity in all cranial-SSCs and femur-SSCs, with cranial-SSCs demonstrating significantly lower chondrogenic activity in comparison to femur-SSCs (Fig. 2e).

We further demonstrated the intrinsic functional activity of cranial-SSCs in vivo using renal capsule transplantation. SSCs isolated from P3 mice from each region (100K cells/graft) were transplanted into the kidney capsule. Four weeks post-transplantation, we harvested the grafts, sectioned, and performed pentachrome staining to evaluate the formation of bone, cartilage, and stroma by the transplanted cells. As previously reported, femur-SSCs were capable of forming bone, cartilage and stroma (Fig. 2c, f). Interestingly, occipital-SSCs predominantly formed bone, and nasal-SSCs exclusively formed cartilage, while parietal- and mandibular-SSCs formed a mixture of bone, cartilage, and stromal tissue (Fig. 2c, f). Computed tomography (CT) scans of the kidneys further confirmed bone formation arising from transplanted femur-, mandibular-, occipital-, and parietal-SSCs but not nasal-SSCs (Supplementary Fig. 6).

To examine whether the observed lineage biases are associated with differences in intrinsic transcriptional regulation, we analyzed chondrogenic and osteogenic gene expression by qPCR. Interestingly, chondrogenic genes (*Acan, Col2a1, Col9a1*, and *Sox9*) were all significantly downregulated in mandibular-SSCs (Fig. 2g), whereas osteogenic genes (*Col1a1*, *Sp7*, and *Bglap*) were significantly upregulated in these cells (Fig. 2h). This transcriptomic profile was consistent with kidney capsule implantation of mandibular-SSCs resulting predominantly in bone formation (Fig. 2f), collectively indicating that mandibular-SSCs are intrinsically biased toward the osteogenic lineage. Occipital-SSCs showed elevated *Runx2* expression and reduced expression of late osteogenic genes (*Col1a1* and *Alpl*) by qPCR, indicating an early osteoprogenitor-like state with strong osteogenic potential (Fig. 2h). In nasal-SSCs, *Spp*1 and *Alpl* expression levels were significantly lower compared with femur-SSCs, while chondrogenic gene expression was comparable (Fig. 2g, h). This profile indicates reduced osteogenic potential in nasal-SSCs, consistent with their functional bias away from osteogenesis and toward chondrogenic differentiation (Fig. 2c–f). Together, these results demonstrate that immunophenotypically defined SSCs from different cranial regions display distinct functional activities that are, at least in part, supported by intrinsic transcriptional differences.

We also examined the expression of previously identified cranial-SSC marker genes (Supplementary Fig. 7). *Ctsk* expression was significantly higher in parietal-SSCs compared with femur-SSCs, consistent with prior reports identifying Ctsk^+^ periosteal progenitor cells enriched in craniofacial bones^6^, particularly within the parietal region. *Zic1* expression was also significantly elevated in parietal- and occipital-SSCs (Supplementary Fig. 7), aligning with the findings of Sun et al.^25^, who identified Zic1^+^ SSCs in vertebral bone of paraxial mesodermal origin. This suggests that elevated *Zic1* expression in parietal- and occipital-SSCs may reflect their shared paraxial mesoderm lineage identity. The transcriptional regulators *Msx1* and *Msx2* exhibited distinct expression patterns: *Msx1* was primarily expressed in nasal-SSCs, while *Msx2* was active in both parietal- and occipital-SSCs (Supplementary Fig. 7). This observation is consistent with prior findings that calvarial osteoprogenitor cell activity is mediated by *Msx* expression^30^. Overall, these results define a functional and molecular map of cranial-SSC diversity, providing a foundation for region-tailored regenerative strategies that account for intrinsic stem cell biases.

### Cranial SSC and BCSP dynamics changes over the course of postnatal development

**Fig. 3:**
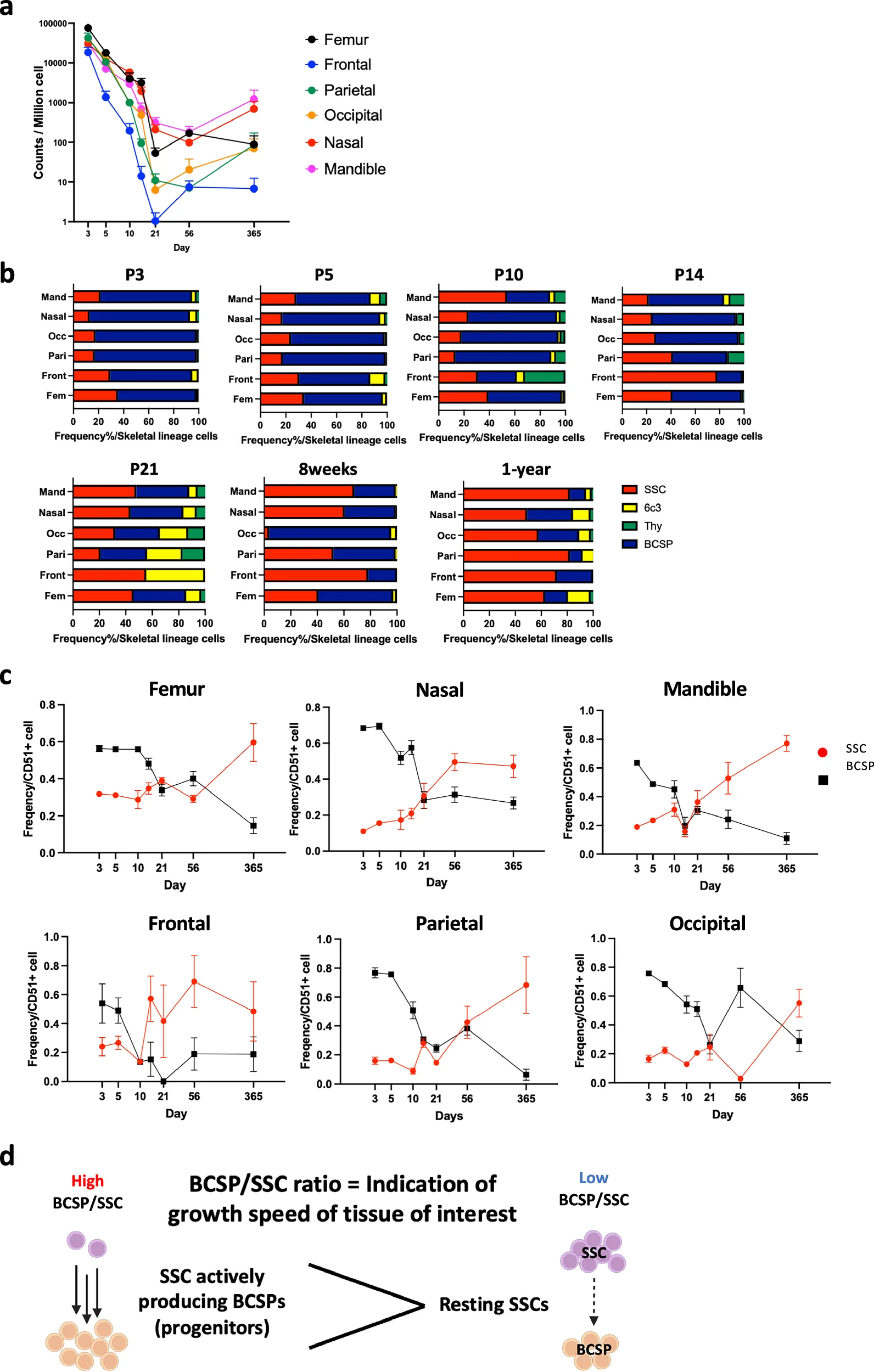
Cranial SSCs and BCSPs undergo dynamic changes over developmental time. **a** SSC counts per million cells (quantified based on flow cytometric analysis), from indicated bones at indicated postnatal developmental timepoints. Data are shown on a logarithmic scale, and error bar is S.E.M. (*n* = 4 mice). **b** Relative frequency of indicated cell types (SSCs, red; BCSPs, blue; Thy+ cells, green; 6c3+ cells, yellow) among all CD45-Tie2-Ter119-CD51+ (skeletal lineage) cells in indicated bone types (“Mand,” mandible; “Occ,” occipital; “Pari,” parietal; “Front,” frontal; “Fem,” femur), at indicated developmental timepoints (*n* = 4 biological replicates per condition). **c** Frequency of SSCs (red) and BCSPs (black) as a proportion of the CD45-Tie2-Ter119-CD51+ cell population at indicated developmental timepoints (*n* = 4 mice, error bar is S.E.M). **d** Schematic illustration of the concept of BCSP/SSC ratio as an indicator of skeletal tissue development. Created in BioRender. Takematsu, E. (2026) https://BioRender.com/0ijpw5l.

Next, we investigated temporal changes in both the frequency and quantity of SSCs, which could offer vital insights for customizing skeletal tissue regeneration to align with patient age. Because FACS-based analysis requires relatively few cells, the frontal bone was included in this experiment despite being excluded from downstream functional assays due to limited SSC yield in the previous section. First, we quantified changes over time by counting SSCs and their descendants, bone cartilage stromal progenitors (BCSPs), which we isolated by using previously established markers^4^. We tracked SSC and BCSP counts per million events from various ages of mice: P3, P5, P10, P14, P21, 8 weeks, and 1 year (Supplementary Fig. 8a–g). Early on (P3 and P5), SSC counts were higher in the femur compared to cranial bones (Fig. 3a). Notably, there was a decrease in SSC counts by P21 for all sites; this was followed by an increase in SSCs in cranial bones, while femur-SSC counts remained relatively consistent post-P21 (Fig. 3a). Comparing across all regions at P21, there were significantly more SSCs derived from the mandible and nasal bones compared to other areas (Supplementary Fig. 8h). This trend continued to 1 year postnatally, at which point the number of mandibular-SSCs remained significantly greater compared to other regions (Supplementary Fig. 8i). Importantly, SSC enrichment within the mandible is primarily localized to the periodontal ligament-associated alveolar bone (Fig.1f), which arises from dental follicle-derived mesenchyme. Therefore, these findings likely reflect progenitor dynamics within tooth-associated niches and may not uniformly represent all mandibular regions, such as the body or ramus. Our time course analysis indicates that SSC dynamics are fundamentally distinct between cranial and long bones: in early stages (P3-P5), femur-SSCs outnumber cranial-SSCs, yet in later stages (P21 and beyond), mandibular- and nasal-SSCs surpass femur-SSCs. Interestingly, it has been shown that the mandible generally heals more rapidly than the femur, with mandibular recovery occurring within 4–8 weeks, compared to femoral healing taking 3–6 months or longer^31,32^. This difference in healing speed, which persists into adulthood, could be attributable to the mandible’s higher frequency of SSCs.

We also assessed the prevalence of SSCs and BCSPs within the skeletal lineage by measuring their frequency as a proportion of the CD45-Tie2-Ter119-CD51+ cell population. Since the CD45-Tie2-Ter119-CD51+ population represents all skeletal lineage cells, the proportion of SSCs or BCSPs within this group reflects the extent of stem cell composition of the skeletal lineage. We quantified the prevalence of four cell populations—SSCs, BCSPs, 6c3+ (stromal) cells, and Thy+ (bone/cartilage) cells—within skeletal lineage cells from each site (Fig. 3b). Of these four cell types, SSCs are the least differentiated; BCSPs are more lineage-committed compared to SSCs, and 6c3+ and Thy+ cells are further differentiated from BCSPs^4^. At P3, SSCs comprised 30%, and BCSPs made up 70% of skeletal lineage cells from the femur (Fig. 3b, top left panel). Cranial bones at this time point had a lower relative incidence of SSCs and a higher frequency of BCSPs compared to the femur. This difference was most evident in the nasal bone, where SSCs constituted only 10% of skeletal lineage cells at P3, while BCSPs constituted 70%. At P21, we noted a higher frequency of more lineage-committed cell types like the 6c3+ and Thy+ populations in the cranial bones compared to earlier time points (Fig. 3b, bottom left panel), consistent with a phase of rapid tissue development. The P21 time point in mice is analogous to around 2–3 years of age in human development, corresponding to a crucial phase of cranial bone growth^33^. An intriguing pattern we observed was that at early time points, BCSPs outnumbered SSCs in both femur and cranial bones, but this trend was reversed in aged, 1-year-old mice (Fig. 3c). This shift is consistent with distinct developmental requirements over time: in early life, the femur and cranial bones are actively developing and growing, necessitating a large number of BCSPs to generate more differentiated cells, such as osteoprogenitors and chondroprogenitors, whereas demand for these intermediate progenitor cells is lessened later in life. Based on our findings from this time course analysis of skeletal progenitor cells, we propose that the BCSP/SSC ratio can serve as a functionally relevant indicator of skeletal development (Fig. 3d). A higher BCSP/SSC ratio suggests that the tissue is in an active stage of development (with active growth necessitating stem cell differentiation into specific skeletal tissues and, therefore, a higher presence of BCSPs compared to SSCs). Consistent with this concept, the elevated BCSP/SSC ratios that we observed in the nasal, parietal, and occipital bones during early developmental periods (Supplementary Fig. 8j–n) align well with times of active cranial bone development. Notably, the BCSP/SSC ratio for the mandibular bone peaks at 2 weeks (P14) (Supplementary Fig. 8n). This transient increase occurs during the period of rapid postnatal mandibular growth (P7–P21), when mouse pups begin transitioning toward solid food while continuing to nurse^34^. Although the underlying mechanism remains unclear, the elevated BCSP/SSC ratio may reflect the developmental demands associated with mandibular maturation. Beyond P14, the BCSP/SSC ratio subsequently decreases, suggesting progression beyond this transient developmental stage.

### Cranial-SSCs’ differential response to BMP-2 stimulation

**Fig. 4:**
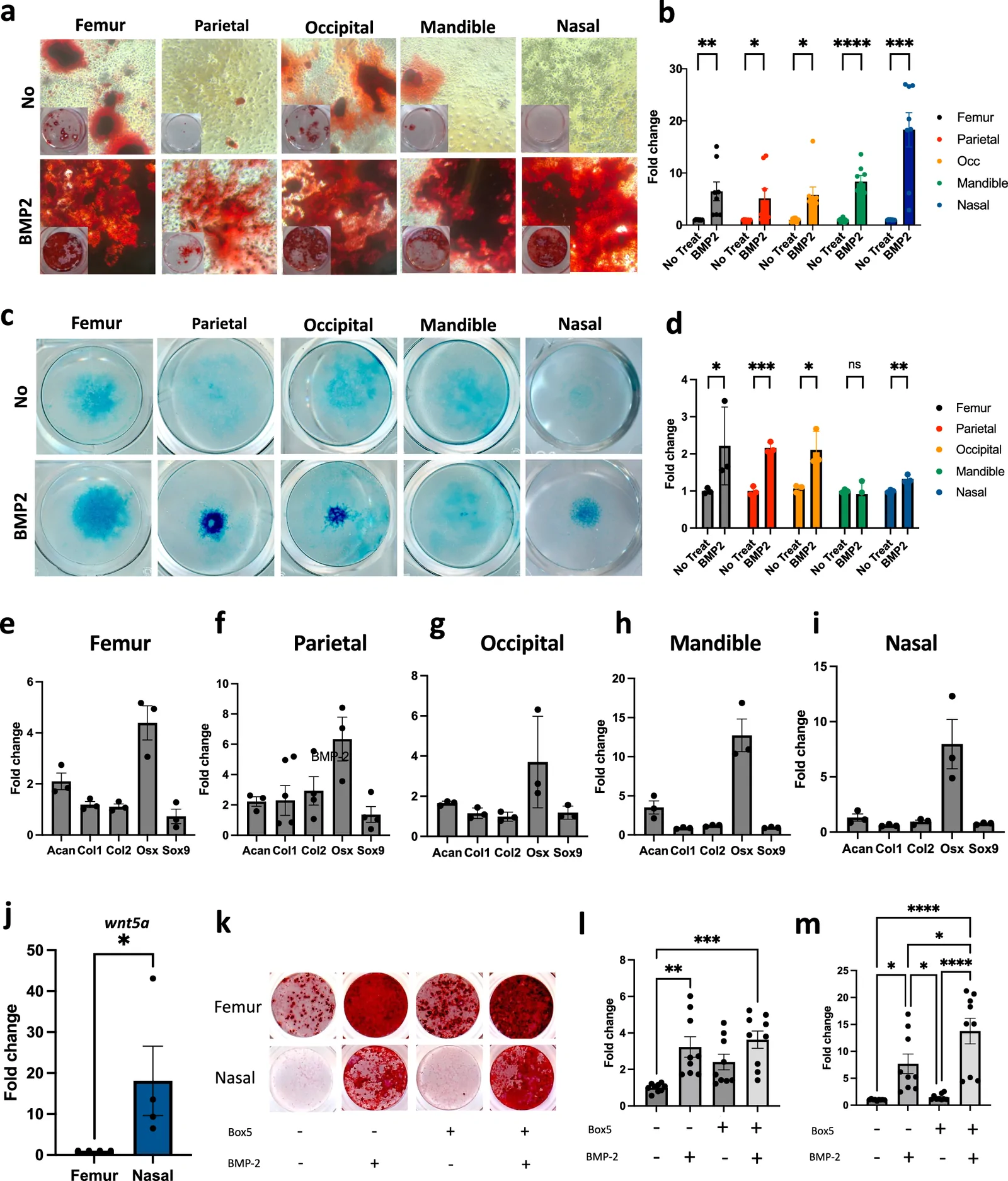
Cranial-SSCs exhibit site-specific responses to BMP-2 stimulation. **a** Representative Alizarin Red staining images of SSCs from indicated bones following an osteogenic differentiation assay without (top row) or with (bottom row) added BMP-2 (100 ng/mL). Inset smaller images show entire wells; larger images show higher magnification of representative regions. **b** Quantification of Alizarin Red staining intensity for SSC osteogenic differentiation assay shown in (**a**), with values for BMP-2 treated condition (“BMP2”) normalized to non-BMP-2-treated control (“No”) for each bone (*n* = 3 biological replicates/8 technical replicates per condition). **c** Representative Alcian Blue staining images of SSCs from indicated bones following chondrogenic differentiation assay without (top row) or with (bottom row) added BMP-2 (100 ng/mL). **d** Quantification of Alcian Blue staining intensity for SSC chondrogenic differentiation assay shown in (**c**), with values for BMP-2 treated condition (“BMP2”) normalized to non-BMP-2-treated control (“No”) for each bone (*n* = 3 biological replicates per condition). **e**–**i** qPCR analysis of indicated osteogenic and chondrogenic gene expression in SSCs from indicated bones, showing fold difference in expression for SSCs treated with BMP-2 (100 ng/mL) compared to non-BMP-2-treated control cells (*n* = 3–4 biological replicates per condition). **j** qPCR analysis of relative Wnt5a expression in femur- and nasal-SSCs (*n* = 4 biological replicates), normalized to femur-SSC expression levels for each replicate. **k** Representative Alizarin Red staining images of osteogenic differentiation assay for femur (first row) and nasal (second row) SSCs, with and without treatment with Wnt5a inhibitor Box5 (100 µM) and/or BMP-2 (100 ng/mL) as indicated. Quantification of Alizarin Red staining intensity in femur-SSCs (**l**) and nasal-SSCs (**m**) following osteogenic differentiation assay, with and without Box5 and/or BMP-2 treatment as indicated (*n* = 3 biological replicates/9 technical replicates). Values show fold change normalized to untreated (Box5- BMP-2-) control SSCs from each bone. **b**, **d**, **e**–**j**, **l**, **m** All data are presented as mean ± SEM. Statistical significance was determined by t-test (two tailed) to compare no treatment and BMP-2 treatment in (**b**, **d**), and one-way ANOVA with a Tukey post-hoc test to compare multiple samples in (**l**, **m**); for indicated comparisons, *p < 0.05, **p < 0.01, ***p < 0.001, and ****p < 0.0001.

Given the functional diversity that we uncovered within cranial-SSCs, we next questioned how this heterogeneity could influence the outcomes of bone regenerative therapies. The effectiveness of BMP-based bone regeneration therapy has been found to vary significantly across different cranial locations. We hypothesized that this variability in therapeutic response may arise from the inherent diversity of SSCs, given these cells’ central role in bone regeneration. To understand how SSC diversity affects the response to bone regenerative treatments, we probed SSC responses to BMP-2, a protein known for its robust bone regenerative capabilities that is commonly used for calvarial bone repair. We first evaluated the osteogenic response of cranial-SSCs to BMP-2 treatment (100 ng/mL), compared to that of femoral-SSCs (Fig. 4a). Nasal SSCs exhibited the strongest response to BMP-2, demonstrating the highest fold change and more pronounced mineralization compared with SSCs from other regions (Fig. 4b). The order of BMP-2 sensitivity for mineral deposition was nasal > mandibular > femur >  occipital > parietal-SSCs. Next, we assayed chondrogenesis and quantified extracellular matrix (ECM) deposition in response to 100 ng/ml BMP-2 treatment (Fig. 4c). Among cranial-SSCs, parietal-, occipital-, and nasal-SSCs exhibited significant ECM accumulation after BMP-2 treatment, whereas mandibular SSCs had negligible response to BMP-2 (Fig. 4d).

To explore the transcriptional basis of these regional differences, we examined the expression of key osteogenic and chondrogenic markers following 3 days of BMP-2 treatment (100 ng/mL) using qPCR (Fig. 4e–I show changes in expression in response to BMP-2 treatment, compared to untreated controls). In femur-, parietal- and occipital-SSCs, *Acan* and *Osx* were upregulated approximately two-fold and four-fold, respectively (Fig. 4e–g), consistent with both osteogenic and chondrogenic differentiation observed in these cells in response BMP-2 (Fig. 4b, d). Mandibular-SSCs showed a dramatic (12-fold) induction of *Osx* versus a much weaker (only three-fold) increase in *Acan*, consistent with their robust bone mineral deposition but limited ECM accumulation observed in vitro following BMP-2 treatment (Fig. 4a, c). Nasal-SSCs exhibited a seven-fold increase in *Osx* expression, consistent with their strong osteogenic response to BMP-2 (Fig. 4b). Collectively, these results demonstrate substantial variation in BMP-2 responsiveness among cranial SSCs, with nasal-SSCs showing the most pronounced osteogenic activation.

We next investigated the mechanisms underlying these divergent osteogenic capacities, focusing on the striking BMP-2 responsiveness we observed in nasal-SSCs. Bulk RNA-sequencing revealed that *Wnt5a* expression was significantly higher in nasal-SSCs compared with femur-SSCs (Supplementary Fig. 9a), which was confirmed by qPCR showing nearly 20-fold higher *Wnt5a* transcript levels in nasal- versus femur-SSCs (Fig. 4j). Based on previous study showing that non-canonical Wnt signaling can suppress osteogenesis in cranial stem cells^35^, we hypothesized that elevated *Wnt5a* suppresses osteogenic differentiation through enhanced non-canonical Wnt signaling. To test this, we assessed osteogenesis in femur- and nasal-SSCs treated with Box5 (100 µM), a selective Wnt5a antagonist, in the presence or absence of BMP-2 (100 ng/mL) (Fig. 4k). As expected, BMP-2 treatment alone promoted mineralization in both femur- and nasal-SSCs (Fig. 4l, m, first and second bars). However, when Box5 was administered without BMP-2, neither femur- nor nasal-SSCs demonstrated substantial enhancement in mineral deposition relative to untreated controls (Fig. 4l, m). In contrast, Box5 treatment combined with BMP-2 resulted in increased osteogenesis compared with BMP-2 alone, with a modest improvement observed in femur-derived SSCs and a significantly greater enhancement in nasal-SSCs (Fig. 4l, m). These data refine our initial model and indicate that Wnt5a primarily acts to restrain BMP-responsive osteogenesis rather than suppress basal osteogenic differentiation. In femur-SSCs, low basal Wnt5a expression imposes minimal inhibition, resulting in only a slight enhancement when Box5 is added to BMP-2. In contrast, the high Wnt5a expression in nasal-SSCs creates a strong inhibitory threshold that can be relieved when both BMP-2 stimulation and Wnt5a blockade are present. Thus, the reduced osteogenic potential of nasal SSCs compared with femur-derived SSCs under BMP-2 stimulation (Fig. 4k) appears to be driven, at least in part, by elevated non-canonical Wnt signaling mediated by Wnt5a, which restricts their responsiveness to osteoinductive cues, such as BMP-2. BMP-2 may interact with Wnt5a signaling^36^; this interaction may contribute to the enhanced effect of Wnt5a inhibition by Box5 in the presence of BMP-2. However, as we did not directly assess Wnt pathway activity following BMP-2 stimulation in this study, the precise interaction between BMP-2 and Wnt5a remains to be determined.

### Cranial-SSCs exhibit region-specific lineage responses to BMP-2 in vivo

**Fig. 5:**
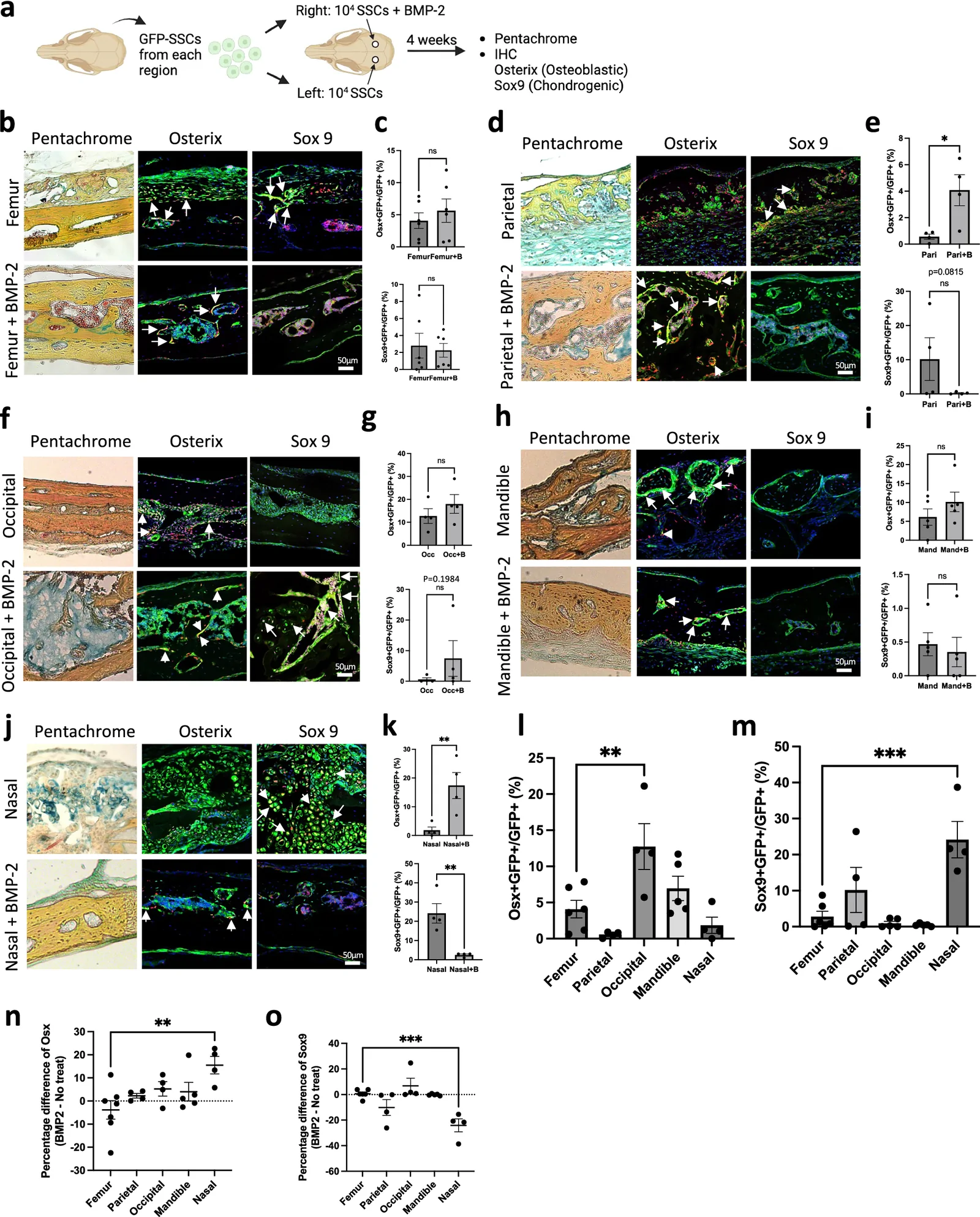
Cranial-SSCs exhibit distinct lineage biases and differential BMP-2 responses in vivo. **a** Schematic illustration of the calvarial defect transplantation experiment to assay SSC activity in vivo. Created in BioRender. Takematsu, E. (2026) https://BioRender.com/8tyiqep. Representative images of Pentachrome staining (first column), and IHC staining for Osterix (middle column) and Sox9 (right column), in calvarial defects implanted with **b** femur-, **d** parietal-, **f** occipital-, **h** mandibular-, and **j** nasal-SSCs, respectively. All images are sectioned in the coronal plane. Top row: SSC-only implants. Bottom row: SSC + BMP-2 implants. For IHC, green indicates implanted SSCs (GFP), red indicates Osterix or Sox9 staining, and blue indicates DAPI nuclear staining. White arrows mark double-positive GFP^+^/Osterix^+^ or GFP^+^/Sox9^+^ cells, representing SSCs differentiating toward the osteoblastic (Osterix^+^) or chondrogenic (Sox9^+^) lineage. Quantitative analysis of lineage differentiation of transplanted **c** femur-, **e** parietal-, **g** occipital-, **i** mandibular-, and **k** nasal-SSCs, respectively, without (left bar) and with (right bar) BMP-2 treatment, based on IHC for Osterix and Sox9. Top: percentage of Osterix^+^GFP^+^ cells over total GFP^+^ cells. Bottom: percentage of Sox9^+^GFP^+^ cells over total GFP⁺ cells (*n* = 4–6 biological replicates per condition, error bar is S.E.M.). Comparison of percentage of transplanted SSCs (GFP^+^) that were **l** Osterix^+^ or **m** Sox9^+^ between SSCs from indicated bones (*n* = 4–6 biological replicates per condition, error bar is S.E.M.). Data reflect non-BMP-2-treated SSCs. Difference in the percentage of **n** Osterix^+^ or **o** Sox9^+^ transplanted SSCs between the BMP-2 treated condition and the control (non-treated) condition. Positive values indicate increased osteoblastic (**n**) or chondrogenic (**o**) commitment with BMP-2 treatment; negative values indicate decreased osteoblastic (**n**) or chondrogenic (**o**) commitment with BMP-2 treatment. Statistical significance was determined using a t-test (two-tailed) to compare two samples and one-way ANOVA with a Tukey post-hoc test to compare multiple samples. *p < 0.05, **p < 0.01, ***p < 0.001, and ****p < 0.0001.

Lastly, we examined how the fate of cranial-SSCs changes upon BMP-2 stimulation in vivo. GFP^+^ SSCs were harvested from GFP-transgenic mice, embedded in 1% alginate gel, and implanted into 1 mm calvarial defects (10^4^ SSCs/defect) with or without added BMP-2 (1 µg/defect). After 4 weeks, tissues were harvested for histological analysis, with Pentachrome staining to assess matrix composition, and immunohistochemistry (IHC) for Osterix (Osx) and Sox9 to trace osteogenic and chondrogenic differentiation, respectively (Fig. 5a). We focused on the 4-week endpoint to assess overall regenerative outcome, while recognizing that earlier stages, such as day 7, may be informative and warrant future investigation. While local niche factors may contribute to the regenerative outcome, parietal bone was selected as the defect site because it is a standardized and well-established location for consistent comparison across SSC populations.

For femur-SSCs transplanted without BMP-2, both GFP^+^/Osx^+^ and GFP^+^/Sox9^+^ populations were detected (Fig. 5b, c), suggesting activation of both osteogenic and chondrogenic-associated lineage programs among transplanted cells. Although the GFP^+^/Sox9^+^ cells did not exhibit the classical morphology of mature chondrocytes, Sox9 expression may reflect cartilage-associated lineage activity in the regenerative environment. Pentachrome staining showed mainly bone formation in this experimental condition (Fig. 5b, top left), supporting the interpretation that Sox9^+^ cells may represent a transient cartilage-associated component during ossification rather than mature cartilage tissue. Upon BMP-2 addition, femur-SSC implants contained predominantly GFP^+^/Osx^+^ cells, with a modest but nonsignificant decrease in GFP^+^/Sox9^+^ cells (Fig. 5b, c).

In defects implanted with parietal-SSCs, the non-BMP-2-treated condition exhibited primarily GFP^+^/Sox9^+^ cells and few GFP^+^/Osx^+^ cells, consistent with Pentachrome staining revealing abundant cartilage-like matrix (Fig. 5d, e). BMP-2 treatment of parietal-SSCs significantly increased the proportion of GFP^+^/Osx^+^ cells and reduced GFP^+^/Sox9^+^ cells, with Pentachrome staining confirming predominantly bone formation (Fig. 5d, e). Occipital-SSC implantation without BMP-2 resulted mainly in bone formation with numerous GFP^+^/Osx^+^ cells (Fig. 5f, g). Interestingly, BMP-2 treatment of occipital-SSCs induced cartilage formation, characterized by increased GFP^+^/Sox9^+^ cells and Alcian Blue-positive matrix on Pentachrome staining (Fig. 5f, g). Given the posterior Hox^+^ identity of occipital-SSCs (Supplementary Fig. 9b), this response likely reflects a positional bias that directs BMP/TGF-β signaling toward a transient Sox9-dependent, endochondral-like program in vivo.

Mandibular-SSC implantation produced distinct ring-like ossification structures, composed of GFP^+^/Osx^+^ osteoprogenitors surrounding marrow-like cavities (Fig. 5h). Similar to BMP-2-treated parietal SSCs, mandibular-SSC implants exhibited predominantly osteogenic features with minimal GFP^+^/Sox9^+^ cells (Fig. 5h, i), consistent with a direct osteogenic differentiation trajectory. Upon BMP-2 treatment, the proportion of GFP^+^/Osx^+^ cells increased slightly (Fig. 5h, i), suggesting that mandibular-SSCs primarily respond to BMP-2 by enhancing an already osteogenic program rather than initiating a cartilage-associated trajectory. Nasal-SSC implantation resulted predominantly in cartilage-like tissue characterized by abundant GFP^+^/Sox9^+^ cells and minimal bone matrix (Fig. 5j, k). This pattern reflects a strong intrinsic chondrogenic bias under low BMP signaling conditions. In contrast, BMP-2 treatment markedly shifted the repair outcome toward bone formation, yielding extensive mineralized matrix and numerous GFP^+^/Osx^+^ osteoprogenitors with a corresponding reduction in GFP^+^/Sox9^+^ cells (Fig. 5j, k). Nasal SSCs further contributed to bone formation upon BMP-2 treatment, as indicated by GFP⁺ cells within newly formed bone (Fig. 5j).

Quantitative comparison of the frequency of Osx^+^ and Sox9^+^ cells among GFP^+^ cells from each SSC origin revealed site-specific differences in lineage commitment in the absence of BMP-2 (Fig. 5l, m). Occipital-SSC implants contained a significantly higher proportion of GFP^+^/Osx^+^ cells compared with femur-SSC implants, whereas nasal-SSC implants showed a significantly higher proportion of GFP^+^/Sox9^+^ cells compared to femur-SSC implants (Fig. 5l, m), further highlighting the intrinsic differentiation capacity among cranial-SSCs. To evaluate the effect of BMP-2 across cranial-SSC types, we calculated the percentage change in GFP^+^/Osx^+^ and GFP^+^/Sox9^+^ fractions before and after BMP-2 treatment (Fig. 5n, o). Relative to femur-SSCs, nasal-SSCs exhibited a significantly greater increase in GFP^+^/Osx^+^ cells and a significantly and markedly greater reduction in GFP^+^/Sox9^+^ cells (Fig. 5n, o), indicating a robust osteogenic shift upon BMP-2 stimulation. Overall, BMP-2 induces region-specific lineage responses in cranial SSCs: nasal and parietal SSCs shift from chondrogenic to osteogenic differentiation, mandibular SSCs enhance osteogenesis without initiating cartilage formation, and occipital SSCs exhibit a distinct response with slightly increased chondrogenesis. In summary, these results reveal that SSCs from distinct cranial regions decode BMP-2 signals in unique ways, underscoring the importance of region-specific tuning of BMP-2 delivery for optimal calvarial bone regeneration.

## Discussion

As technological advancements have facilitated more nuanced cell clustering and profiling, recent research has increasingly uncovered anatomical diversity among somatic stem cells, such as muscle stem cells^37^ and adipose stem cells^38^. Similarly, in the field of skeletal biology, emerging evidence had suggested the existence of transcriptomic and functional diversity within SSCs depending on their anatomical location^11^. However, such anatomical diversity within cranial bone SSCs had previously remained unexplored, despite the intriguing premise of cranial bones’ distinct developmental origins (with different regions of the cranium arising from neural crest vs. paraxial mesoderm vs. lateral plate mesoderm—potentially underlying analogous functional diversity among each regions’ resident stem cells). Here, we define, to our knowledge, the first comprehensive molecular and functional map of cranial-SSCs, revealing how positional identity within the cranium governs stem cells’ lineage potential and morphogen responsiveness. These insights provide a framework for understanding cranial bone development and regeneration, and for refining region-specific therapeutic strategies.

### Regional identity underlies SSC heterogeneity

Clonality tracing using Actin^CreER^;R26^VT2/GK3^ mice demonstrated durable clonal expansion of SSCs across cranial regions, with the highest frequency of clones in the nasal bone, followed by the occipital and mandibular regions, and the lowest in parietal and frontal bones. The paucity of long-term clones and colonies in the frontal bone indicates that shorter-term clonal expansion in this bone reflects largely short-lived progenitors rather than bona fide SSCs. As a neural crest-derived bone, the frontal bone may rely more on transient progenitor populations than on a long-lived SSCs. In addition, the frontal bone undergoes active growth and remodeling during the postnatal period analyzed, which may further favor progenitor turnover over maintenance of a stable SSC population. Immunophenotypic isolation further showed variable prevalence and self-renewal capacity of SSCs among cranial sites. Parietal- and occipital-SSCs (isolated based on the markers originally identified by Chan et al. in long bones^4^) exhibited robust CFU formation and multilineage potential, supporting their identity as genuine SSCs. In contrast, mandibular-SSCs, although clonogenic, expressed elevated *Sp7*, *Col1a1*, and *Bglap*, indicating enrichment for osteogenically committed progenitors. These results imply that while canonical long-bone SSC markers effectively enrich SSCs in the context of most cranial bones, mandibular bone may require complementary markers to fully define its true stem cell compartment.

### Functional continuum of cranial-SSCs

**Fig. 6:**
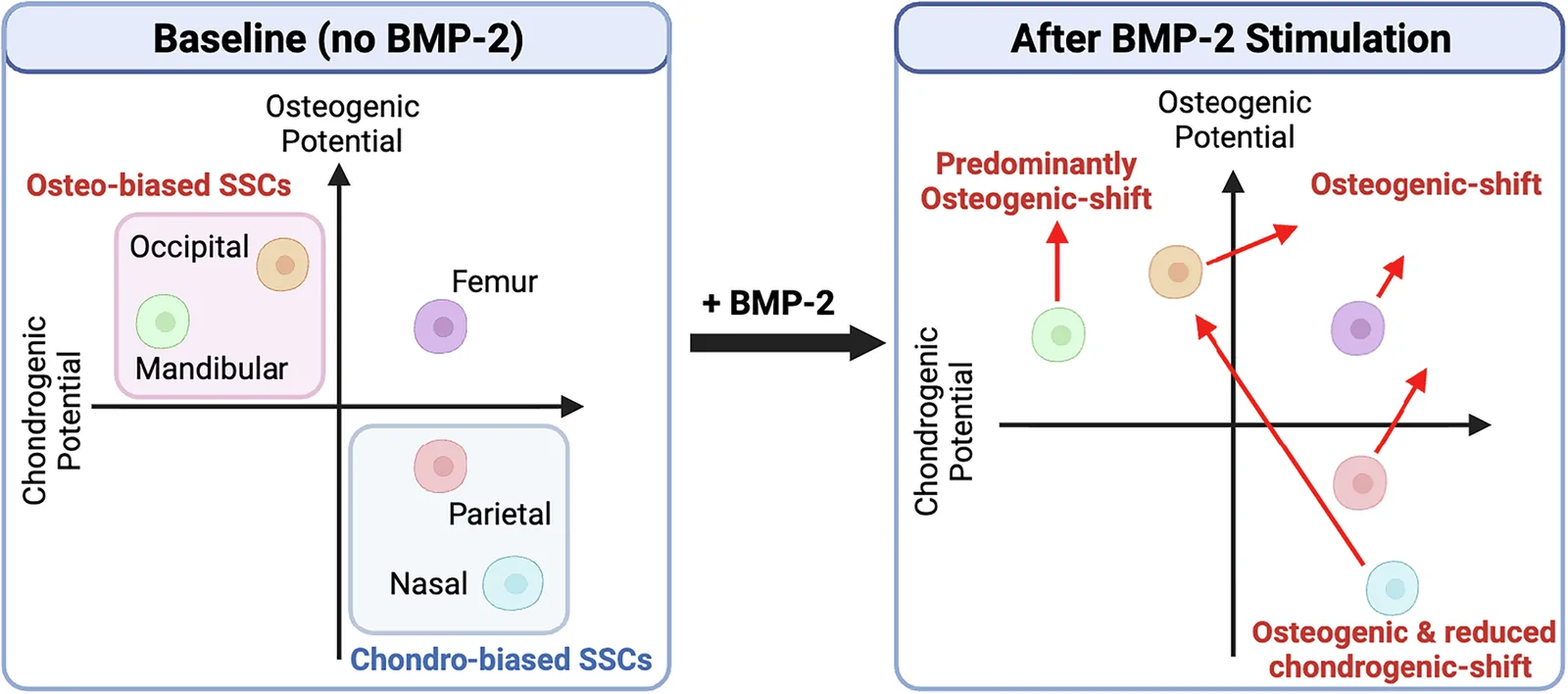
Region-specific lineage bias and BMP-2-induced differentiation shifts in cranial SSCs. Combined in vitro and in vivo functional assays, together with osteogenic and chondrogenic gene expression profiles, reveal that immunophenotypically isolated cranial SSCs exhibit distinct lineage biases. In the basal condition, occipital and mandibular SSCs display reduced chondrogenic potential but strong osteogenic capacity, whereas parietal and nasal SSCs show enhanced chondrogenic potential and diminished osteogenic activity. Upon BMP-2 stimulation, this landscape shifts in a region-specific manner. Red arrows indicate the direction of lineage differentiation, with arrow length representing the magnitude of change in lineage allocation following BMP-2 treatment. Nasal SSCs exhibit the most dramatic response, showing a marked shift toward osteogenesis accompanied by suppression of the chondrogenic program in vivo (Fig. 5j, k), reflected by the prominent upper-left trajectory. Occipital SSCs display mildly enhanced Sox9-associated/chondrogenic features in vivo while maintaining osteogenic potential, represented by an upper-right shift. Parietal SSCs exhibit enhanced osteogenesis in vivo together with preserved chondrogenic responsiveness in vitro, also reflected by an upper-right shift. Femur SSCs demonstrate limited lineage changes in vivo despite enhanced osteogenic and chondrogenic responses in vitro, resulting in a modest upper-right trajectory. Mandibular SSCs respond to BMP-2 predominantly through enhanced osteogenesis with minimal chondrogenic induction. This schematic highlights how intrinsic SSC diversity governs BMP-2 responsiveness and underscores the need for region-specific optimization of BMP-2-based bone regeneration strategies. Created in BioRender. Takematsu, E. (2026) https://BioRender.com/oenubhm.

Comprehensive in vitro and in vivo assays delineated a functional continuum across cranial-SSCs (Fig. 6). Occipital- and mandibular-SSCs exhibited strong osteogenic activity and were classified as osteo-biased, whereas nasal-SSCs displayed limited osteogenesis and a pronounced chondrogenic preference. Nasal SSCs maintained their self-renewal capacity but remained largely chondrogenic under basal conditions. They exhibited osteogenic competence when BMP signaling was enhanced (Fig. 4a), which was further promoted when non-canonical Wnt signaling was inhibited via Wnt5a antagonism (Fig. 4k).

These findings suggest that additional regulatory factors are required to control the fate of nasal SSCs under basal conditions. Furthermore, cell fate decisions may be established at earlier developmental stages (e.g., neonatal time points), warranting further investigation. Parietal-SSCs, in contrast, showed balanced osteogenic and chondrogenic potential with a mild bias toward cartilage formation in vivo, suggesting a partially repressed osteogenic program rather than a fixed lineage restriction. Notably, regional differences in SSC behavior may also reflect distinct microenvironmental influences. For example, the occipital region is associated with greater muscle attachment and mechanical loading, which may promote osteogenic differentiation through niche-dependent cues. Conversely, differences in the local cranial niche may contribute to broader lineage potential in other regions, although this possibility was not directly examined in the present study. Collectively, these findings define a graded spectrum of cranial-SSC states, in which positional identity modulates lineage bias through differential sensitivity to morphogen cues.

### Positional tuning of BMP-2 responsiveness

BMP-2 stimulation elicited distinct lineage outcomes among cranial-SSCs of different regional origins (Fig. 6). Upon BMP-2 treatment, nasal-SSCs exhibited a pronounced shift from SOX9^+^ to OSX^+^ lineages (Fig. 5n, o), with robust bone formation (Fig. 5j), whereas mandibular-SSCs selectively expanded their osteogenic output without initiating chondrogenesis (Fig. 4b, d), consistent with their intrinsic osteogenic bias. Occipital- and parietal-SSCs responded more modestly overall, while femur-SSCs showed a balanced osteo-chondrogenic response. Notably, despite exhibiting high clonal frequency—a feature generally associated with proliferative and self-renewal potential—nasal SSCs predominantly formed cartilage under basal conditions, indicating that proliferative capacity and lineage fate are not necessarily coupled. This suggests that nasal SSCs are highly proliferative yet exhibit a context-dependent lineage bias, requiring additional cues, such as BMP-2, to fully activate osteogenic differentiation. These findings demonstrate that cranial-SSCs decode BMP-2 cues in a region-dependent manner, reflecting positional tuning of BMP pathway activity. This positional specificity may contribute to the site-dependent variability that has been clinically observed in BMP-2-based craniofacial repair, such as the frequent occurrence of nasal stenosis following alveolar cleft reconstruction with BMP-2^39^. Accordingly, our findings support that region-appropriate BMP-2 dosing and delivery strategies should be considered for cranial bone regeneration, rather than simply extrapolating parameters from appendicular bone models.

There are likely additional factors beyond SSC heterogeneity that impact differential regeneration between bone sites. For instance, the observation that mandibular bone generally exhibits superior regenerative capacity compared with the femur likely results from multiple factors beyond SSC behavior—including region-specific vascularization, marrow composition, and mechanical loading. By thoroughly delineating the intrinsic diversity and BMP-2 responsiveness of SSCs, our study provides a framework for future investigations that integrate these other factors—such as biomechanical and vascular contexts—to fully explain and predict site-specific regenerative outcomes.

### SSC dynamics and a developmental activity index

Temporal flow-cytometric analysis revealed persistently higher SSC numbers in nasal and mandibular bones than in the femur from aged (one-year-old) mice, possibly reflecting region-specific physiological stimuli, including mastication in the mandible and local biomechanical or physiological stimulation associated with airflow in the nasal cavity, although this possibility remains speculative. Based on the results of our study, we introduce the BCSP/SSC ratio as a quantitative index of skeletal developmental activity: a higher ratio of BCSPs to SSCs reflects active differentiation and tissue growth. Supporting this construct, we found that craniofacial bones exhibited elevated BCSP/SSC ratios during early postnatal development (P3–P5); notably, mandibular ratios peaked at P14 (Supplementary Fig. 8n), coinciding with the onset of mastication. We further speculate that an increased demand for lineage-committed cells during earlier developmental stages may reduce the relative SSC pool in the nasal bone compared to other cranial regions (Fig. 3c).

Although SSC prevalence does appear to increase after this developmental window, this change likely reflects the accumulation of quiescent, self-renewing SSCs rather than renewed proliferative activity, and is consistent with a transition from growth to maintenance. BCSP/SSC ratios declined uniformly with ageing across all bones, reflecting reduced skeletal turnover. Overall, the BCSP/SSC ratio provides a tractable means to assess and compare developmental and regenerative dynamics across bones.

### Concluding remarks and translational outlook

Together, our findings uncover previously unrecognized functional and signaling heterogeneity among cranial-SSCs. Importantly, these results suggest that craniofacial repair strategies should account for region-specific SSC behavior, as a uniform BMP-2-based approach may not be optimal across different cranial sites. Where feasible, tailoring morphogen delivery and considering region-matched cellular sources may improve regenerative outcomes. Understanding this diversity will be essential for elucidating the cellular basis of craniofacial skeletal disorders and for developing precision, site-specific BMP-2-based regenerative therapies. Our results also lay the groundwork for a cranial-SSC response map that links anatomical origin, lineage bias, and morphogen sensitivity. Such a framework could guide the rational design of region-specific regenerative protocols, biomaterials, and screening platforms. By benchmarking candidate osteoinductive or chondrogenic cues against intrinsic SSC response profiles, this map would enable identification of context-appropriate factor combinations and dosages for each craniofacial site, advancing precision tissue regeneration and personalized surgical planning.

## Methods

### Mice

All mouse experiments complied with all relevant ethical regulations and were conducted under approved protocols by Stanford’s Administrative Panel on Laboratory Animal Care. Mice were maintained at the Stanford University Research Animal Facility in accordance with Stanford University guidelines. Mice were given food and water ad libitum and housed in temperature-, moisture- and light-controlled (12-h light-dark cycle) micro-insulators. For isolating SSCs, B6 mice (C57BL/Ka-Thy1.1-CD45.1; JAX: 000406) with indicated age were used. For renal transplantation recipient mice, 8–12-week-old immunodeficient Rag2/gamma(c) knockout mice were used (both female and male mice were used in equal number). For clonality analysis, we utilized Actin^CreER^;R26^VT2/GK3^ rainbow transgenic reporter mice. These mice were generated by crossing the Actin^CreER^ transgenic mouse with the R26^VT2/GK3^ transgenic mouse, to enable Cre-dependent cell labeling with a four-color reporter construct at the ROSA locus. In this construct, tamoxifen delivery activates Cre recombinase that induces random and specific recombination, causing Cre-positive cells to express 1 of 10 possible color combinations of 4 fluorescent proteins at the ROSA locus: GFP (green fluorescent protein), CFP (cyan fluorescent protein), RFP (red fluorescent protein), or OFP (orange fluorescent protein). Actin is ubiquitously expressed; thus, tamoxifen induction of Actin^CreER^;R26^VT2/GK3^ mice allows for an unbiased in vivo survey of clonal activity by all potential cell types, rather than specific lineage-restricted promoters^40^.

### FACS isolation and analysis of mouse SSCs

The distal femur, frontal, parietal, occipital and nasal bones and mandible were dissected from postnatal day 3 (P3) mice of both sexes. For the frontal bone, the posterior frontal suture was included; for the parietal bone, the sagittal suture was included; and for the occipital bone, the lambdoid suture was included. Dissections of the nasal bone encompassed the maxilloturbinate and nasoturbinate regions. For the mandible, due to the technical difficulty of removing developing molar teeth and to avoid the potential loss of stem cells associated with the periodontal ligament, all tissue compartments—including the molar teeth—were collected. After harvesting the tissue, cells were isolated with a combination of mechanical and collagenase digestion as described previously^41^. The solution was then centrifuged and washed twice with FACS buffer solution (2% FBS/PBS). The cells were then stained for antibodies to the following antigens: CD45 (Biolegend, Cat # 103110), Ter119 (Biolegend, Cat # 116210), Thy1.1 (Biolegend, Cat # 202520), Thy1.2 (Biolegend, Cat # 140331), CD105-biotin (Invitrogen, Cat # 13-1051-82), CD51 (Invitrogen, Cat # 12-0512-82), 6C3 (Biolegend, Cat# 108312), and CD200 (BD Horizon, Cat # 565547)^4^. CD105-biotin was further stained with PE-Cy7 streptavidin (Invitrogen, Cat # 50-112-8721). Propidium iodide was used to label and exclude any dead cells. FACS analysis was performed on an FACS Aria ll Instrument (BD Biosciences) or MA900 (Sonny), using FlowJo v10 software.

### Cell culture

SSCs were maintained in an incubator at 37 °C with 5% CO_2_. For cultivation, MEMalpha (Invitrogen) was supplemented with 100 U/ml penicillin and 1000 U/ml streptomycin (Invitrogen) and 10% FBS.

### Chondrogenesis differentiation

Assays were performed in micromass cultures by pipetting a 10-µl droplet of cell suspension containing 2 × 10^4^ SSCs from femur or each of the cranial bones in the center of each well (24-well plate). Micromasses were cultured for 20 min in the incubator, before the addition of warm chondrogenic medium (high-glucose DMEM (Invitrogen) with 10% FBS, 100 nM dexamethasone, 1 µM L-ascorbic acid-2-phosphate and 10 ng/ml transforming growth factor β1). Cell medium was changed every other day. For BMP treatment, 100 ng/ml of BMP-2 was supplemented for 21 days. At day 21, cells were fixed and stained with 1% Alcian-Blue staining solution (Sigma, Cat # A5268) for 30 min at room temperature. Five hundred microliters of guanidine solution (12 M) was then applied to each well to destain Alcian-Blue dye for 2 weeks. Absorbance at 590 nm was measured for quantification.

### Osteogenic differentiation with and without BMP-2 treatment

2 × 10^3^ SSCs from femur or cranial bones were seeded per well (96-well plate) and supplemented with osteogenic medium (MEMalpha low glucose (Invitrogen) with 10% FBS, 100 nM dexamethasone, 0.2 mM L-ascorbic acid-2-phosphate, and 10 mM β-glycerophosphate) for 21 days. For BMP treatment, 100 ng/ml of BMP-2 was supplemented for 21 days. Cells were then paraformaldehyde (PFA)-fixed and stained with 2% Alizarin Red S (Roth, Cat # 0348.3) in distilled water. Two hundred microliters of destaining solution (10% acetic acid, 20% methanol in DI water) was applied to each well. To quantify osteogenesis, absorbance at 450 nm was read by plate reader.

### CFU assay

SSCs isolated from P3 femur and cranial bones were used for this study. 1000 cells per well were seeded in 6 well plates and cultured for 3 weeks. SSCs were fixed with 4% PFA followed by crystal violet staining, and colony number was counted.

### SSC transplantation in kidney capsule

Renal capsule transplantations were conducted as previously described^4,42^. In brief, in the anaesthetized mouse, a 5-mm dorsal incision was made, and the kidney was exposed manually. Then, a 2-mm incision was created in the renal capsule using a needle bevel, and 10^5^ FACS-purified SSCs isolated from P3 mice were resuspended in 3 μl of Matrigel and transplanted beneath the capsule. The kidney was re-approximated manually, and incisions were closed using sutures and staples. Grafts were collected after 28 days.

### Tamoxifen induction and tissue preparation

Tamoxifen (200 mg per kg (body weight)) was injected via oral gavage to P10 Actin^CreER^;R26^VT2/GK3^ rainbow reporter mice to label clonality. Skeletal tissues were harvested at P45 and P13.5 weeks time points. The Rainbow reporter is a multi-color Cre-dependent marker system with a four-color construct. Once recombination occurs, cells are randomly and genetically marked with a color (one of ten total colors). All daughter cells will inherit that same color. Fixation and decalcification of the isolated cranial bones were performed using 4% PFA at 4 °C for 24 h, followed by 0.4 M EDTA in PBS (pH 7.2) at 4 °C for 2 weeks. Tissues were embedded in optimal cutting temperature compound after 24 h in 30% sucrose. Ten micrometer slices were made with cryo-sectioning. Confocal microscopy was employed to image clonality. Clones were defined as clusters of ≥5 contiguous cells sharing similar spectral signatures, a threshold selected to minimize false-positive identification due to stochastic color overlap. As clone size thresholds and analysis time points are not standardized and vary by tissue context and labeling system, this criterion was chosen to provide a robust estimate of clonal expansion. Clone identification and counting were performed in a blinded manner. For each section, the number of distinct clones per area (mm^2^) were recorded. Due to the stochastic nature of reporter recombination and the limited number of distinguishable colors, some clones with similar color profiles may not have been resolved; therefore, clone counts likely represent an underestimate of true clonal frequency.

### Quantitative PCR measurement of BMP-2-treated SSCs in vitro

SSCs from femur and cranial bones were FACS-isolated, cultured in 24 well plates, and expanded for 3–4 days. When 70% confluency was reached, cells were treated with 100 ng/ml of BMP-2 for 3 days. Trizol was used to harvest RNA, and RNA was extracted by RNeasy Kits (Qiagen, Cat # 74104). Reverse transcription was conducted by RevertAid First Strand cDNA Synthesis Kit (ThermoFisher, K1621), and qPCR was performed using SYBR™ Green PCR Master Mix (ThermoFisher, 4309155). Three to four biological replicates were made per condition, and the average across biological replicates was used for quantification.

### In vivo calvarial implantation assay

Donor cells were harvested from postnatal day 3 (P3) GFP-transgenic mice. After mechanical and enzymatical digestion and washing steps, cells were stained with SSC marker antibodies and isolated by fluorescence-activated cell sorting (FACS). Purified SSCs were expanded in vitro for three days prior to implantation. Cells were embedded in 1% (w/v) alginate gel with or without recombinant BMP-2 (1 µg per graft) and adjusted to a final volume of 3 µl containing 1 × 10^4^ SSCs. Recipient NOD.Cg-Prkdc^scid^ Il2rg^tm1Wjl^/SzJ (NSG) mice were anesthetized, and a 1-cm midline incision was made over the calvarium. Following careful elevation of the periosteum, two 1-mm circular defects were created on the left and right sides of parietal bone using a 1-mm diameter drill bit. A 1-mm non-critical-size defect was selected to maximize donor SSC survival and reproducible engraftment at the feasible cell dose (1 × 10^4^ cells), to preserve periosteal and dural niche cues, and to enable mechanistic assessment of lineage allocation under controlled inflammatory and vascular conditions. The left defect was filled with SSCs alone, and the right defect received SSCs supplemented with BMP-2, allowing each animal to serve as its own internal control. Differences in Osx^+^ and Sox9^+^ cell frequencies were quantified within the same mouse (right defect – left defect). After 4 weeks, mice were perfused transcardially with PBS followed by 2% paraformaldehyde. Calvarial tissues were decalcified in 400 mM EDTA (pH 7.4) for 7-10 days at 4 °C, cryosectioned, and analyzed by immunohistochemistry using anti-Osterix (Abcam, ab209484) and anti-Sox9 (Abcam, EPR14335) antibodies. Pentachrome staining was performed to assess matrix composition.

### Bulk-RNA sequencing of SSCs

P3 pups (*n* = 8–10) were pooled as one biological replicate. We prepare three biological replicates for this experiment. After harvesting tissues, SSCs were isolated using FACS, then expanded for 5 days in vitro prior for sequencing. Cells were then harvested by Trizol. RNA was extracted using RNeasy Kits (Qiagen, Cat # 74104) and sent out for sequencing at Novogene. Quality controls, library preparation and sequencing were performed according to Novogene’s platform. After obtaining the raw data in fastq file, data filtering was performed to remove low-quality nucleotides (Base Quality less than 5). After filtering, more than 96% of the reads were clean read for all samples. To compare gene expression levels under different conditions, the FPKM among different samples were utilized. FPKM data is available in the source data.

### Statistical analyses

Data were considered approximately normally distributed, and variances were comparable across groups. For comparison of more than two groups, ANOVA analysis was used with a Tukey’s post-hoc test. *P* values were considered significant if *P < 0.05, **P < 0.01, ***P < 0.001, or ****P < 0.0001. Statistical analyses were performed using GraphPad Prism 9 (GraphPad). All data points represent biological replicates. All data are presented as mean ± s.e.m.

### Reporting summary

Further information on research design is available in the Nature Portfolio Reporting Summary linked to this article.

## Supplementary information

Supplementary Information

Reporting Summary

Transparent Peer Review File

## Data Availability

Raw RNA-sequencing data are available from the NCBI Sequence Read Archive (SRA) under accession PRJNA1499731. Processed gene expression matrices are available at Figshare (https://doi.org/10.6084/m9.figshare.31968258).
