# Supplementary material for: Regional diversity of cranial skeletal stem cells governs bone morphogenetic protein-2 mediated bone regeneration
Source: Nat Commun. 2026 Aug 6;17:9479. doi: 10.1038/s41467-026-76286-w (PMC13542091; doi:10.1038/s41467-026-76286-w)
Supplement: Supplementary file 1 [file 41467_2026_76286_MOESM1_ESM.pdf]

Supplementary Figure

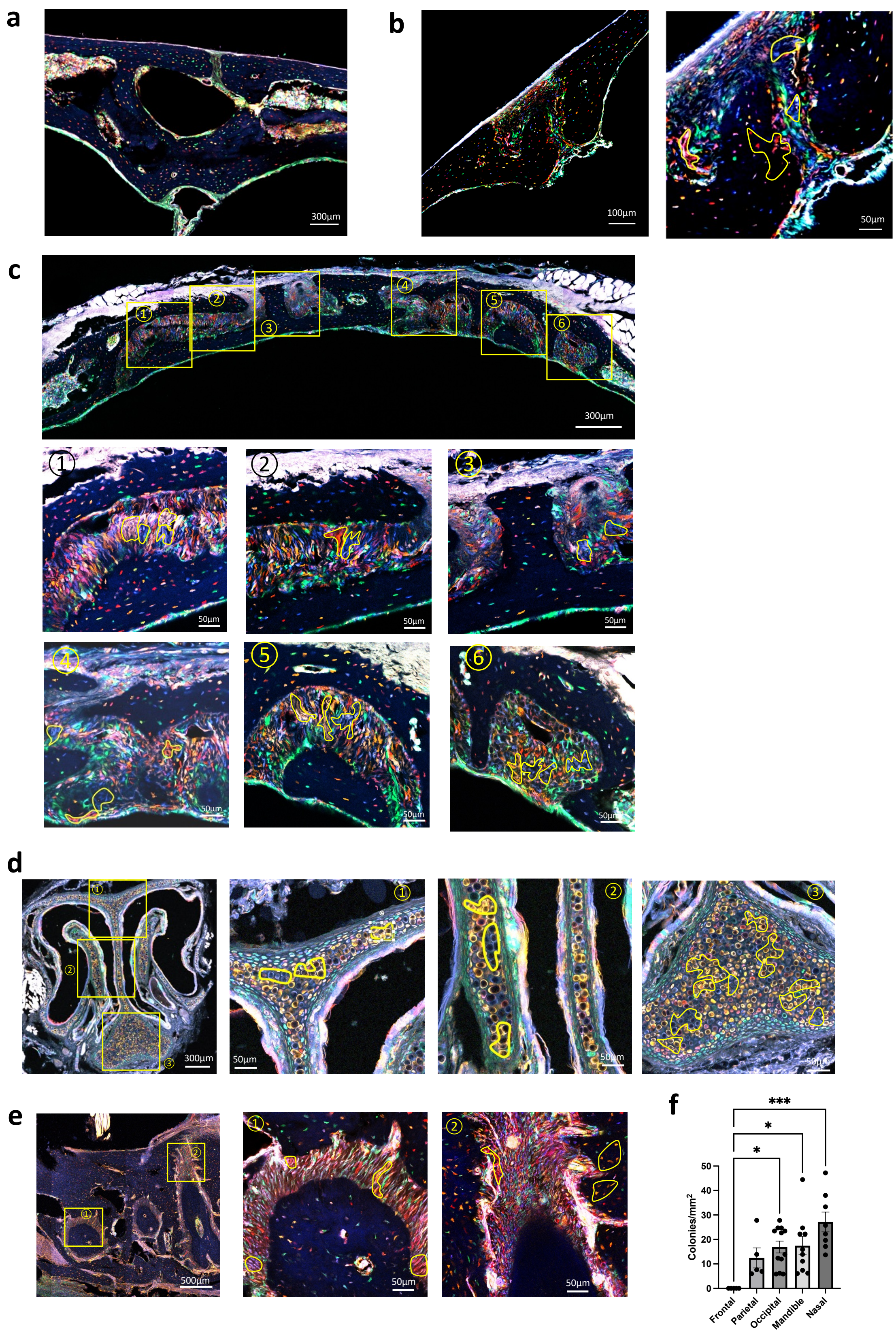

Supplementary Figure 1. Long-term clonal expansion in craniofacial bones of β actin-Cre Rainbow mice (tamoxifen at P10; harvest 12 weeks later). (a-e) Confocal images of the **(a)** frontal bone, **(b)** parietal bone, **(c)** occipital bone, **(d)** nasal bone, and **(e)** mandible of β actin-Cre Rainbow mice which were systemically induced with tamoxifen at postnatal day (P)10 and harvested 12 weeks later. **(b-e)** Yellow outlined regions mark putative clones, defined as ≥5 contiguous cells of the same color. (No clones were detected in frontal bone.) **(b)** Left, 10x; right, 20x magnification. **(c-e)** First panel, low magnification; numbered panels show 20x fields corresponding to numbered outlined regions in first panel. **(f)** Quantification of clone density (clones per mm^2) in indicated bones (n = 4–6 mice per bone; multiple fields were analyzed per region and per mouse). For indicated comparisons, *$p < 0.05$ and ***$p < 0.001$.

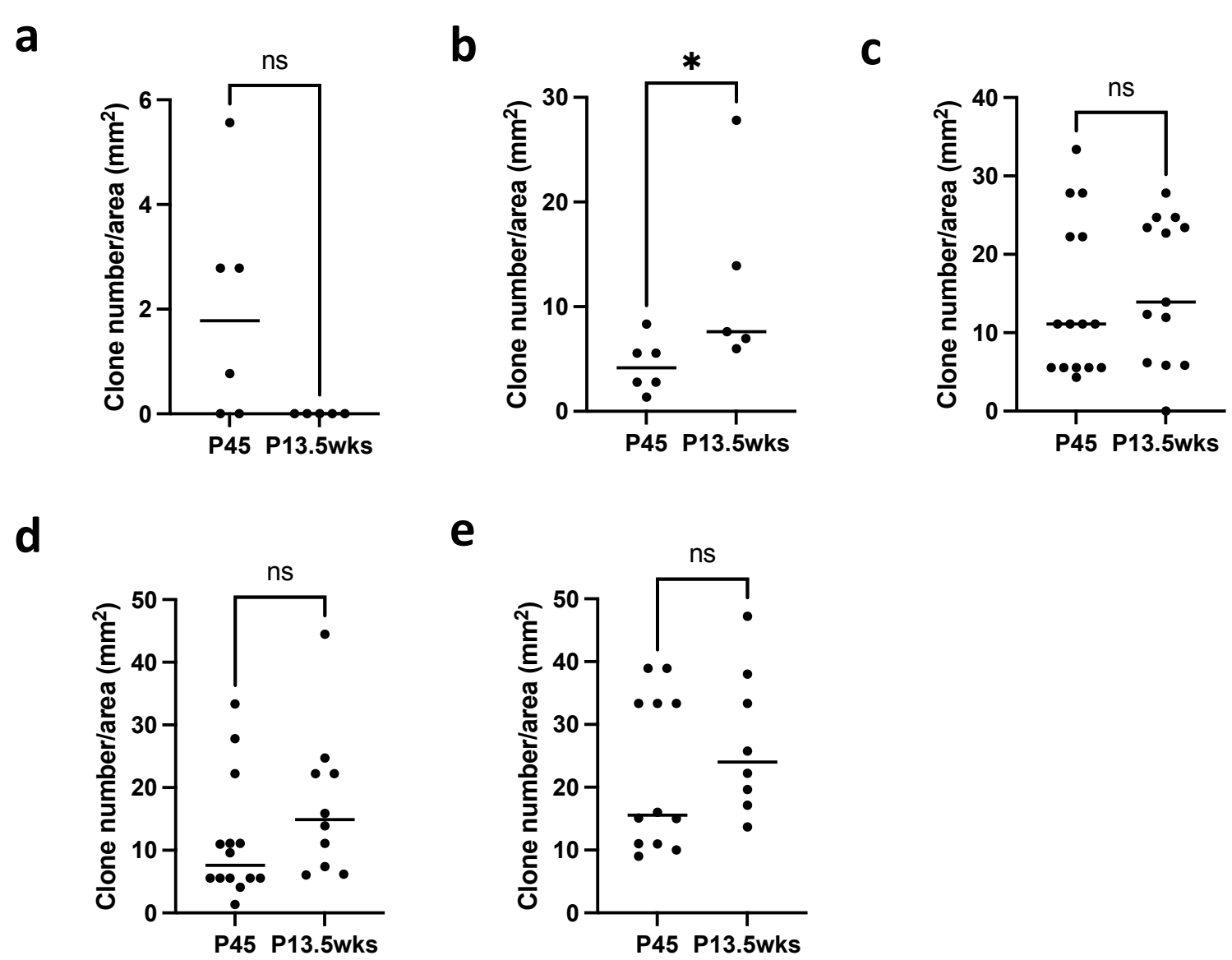

Supplementary Figure 2. Comparison of clone density across cranial bones. (a-e) Quantification of clones per mm^2 in **(a)** frontal bone, **(b)** parietal bone, **(c)** occipital bone, **(d)** mandible, and **(e)** nasal bone, in β actin-Cre Rainbow mice induced with tamoxifen at

P10 and harvested at indicated time points (P45 days and P13.5 weeks). n = 4–6 mice per bone; multiple fields were analyzed per region and per mouse.For indicated comparisons, ns indicates $p > 0.05$; $*p < 0.05$.

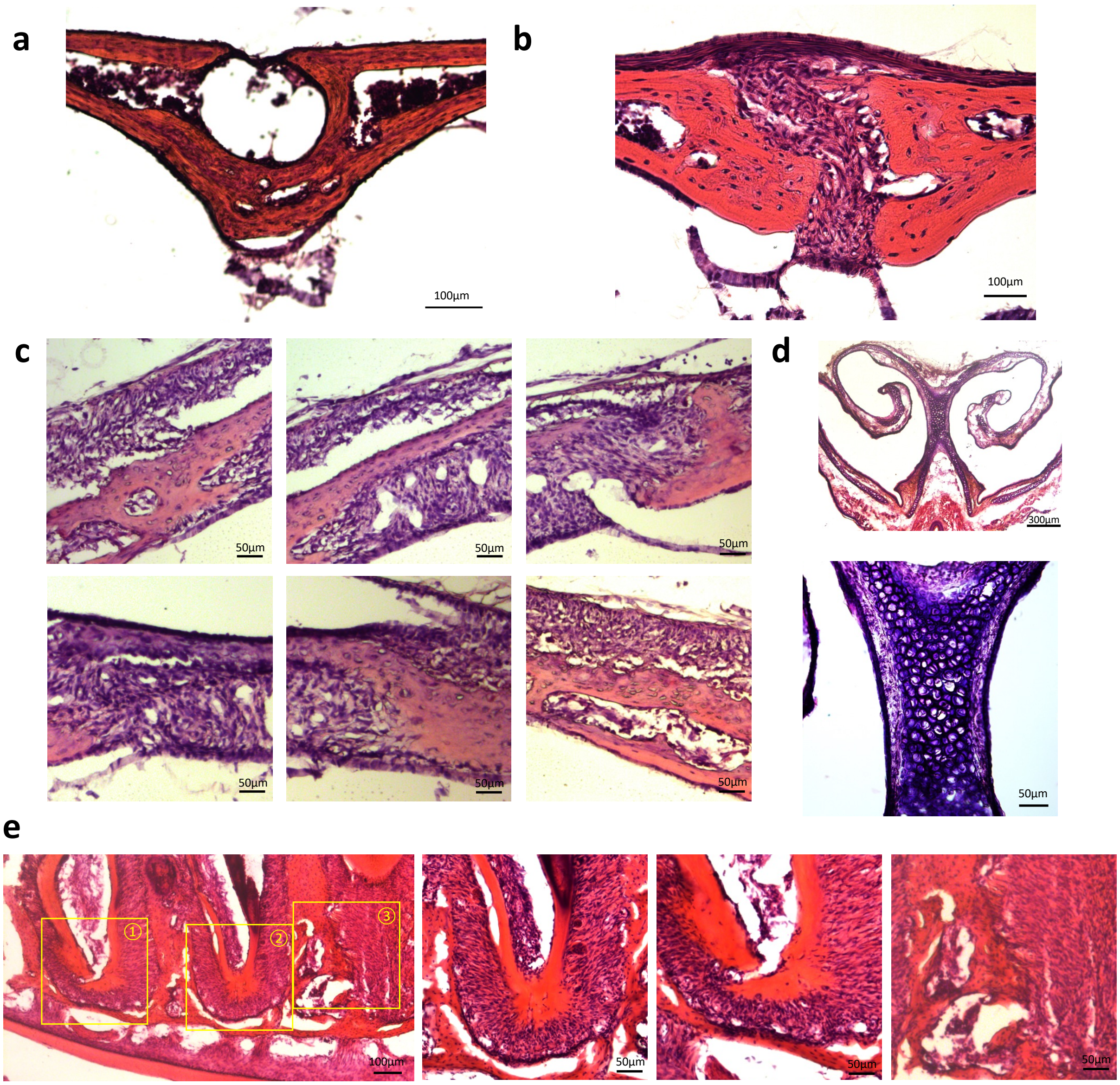

Supplementary Figure 3. Hematoxylin and eosin staining of mouse cranial bones at P45. (a-e) Hematoxylin and eosin (H&E)-stained sections of **(a)** frontal bone, **(b)** parietal bone at the sagittal suture, **(c)** occipital bone, **(d)** nasal bone, and **(e)** mandible, all from β

actin-Cre Rainbow mice (tamoxifen administered at P10, harvested at P45). **(d)** Top, low magnification; bottom, high magnification. **(e)** First panel, low magnification; second through fourth panels, high-magnification views of corresponding numbered regions demarcated in first panel. **(a-e)** Each panel corresponds to the same regions shown in confocal images in **Figure 1b-f**.

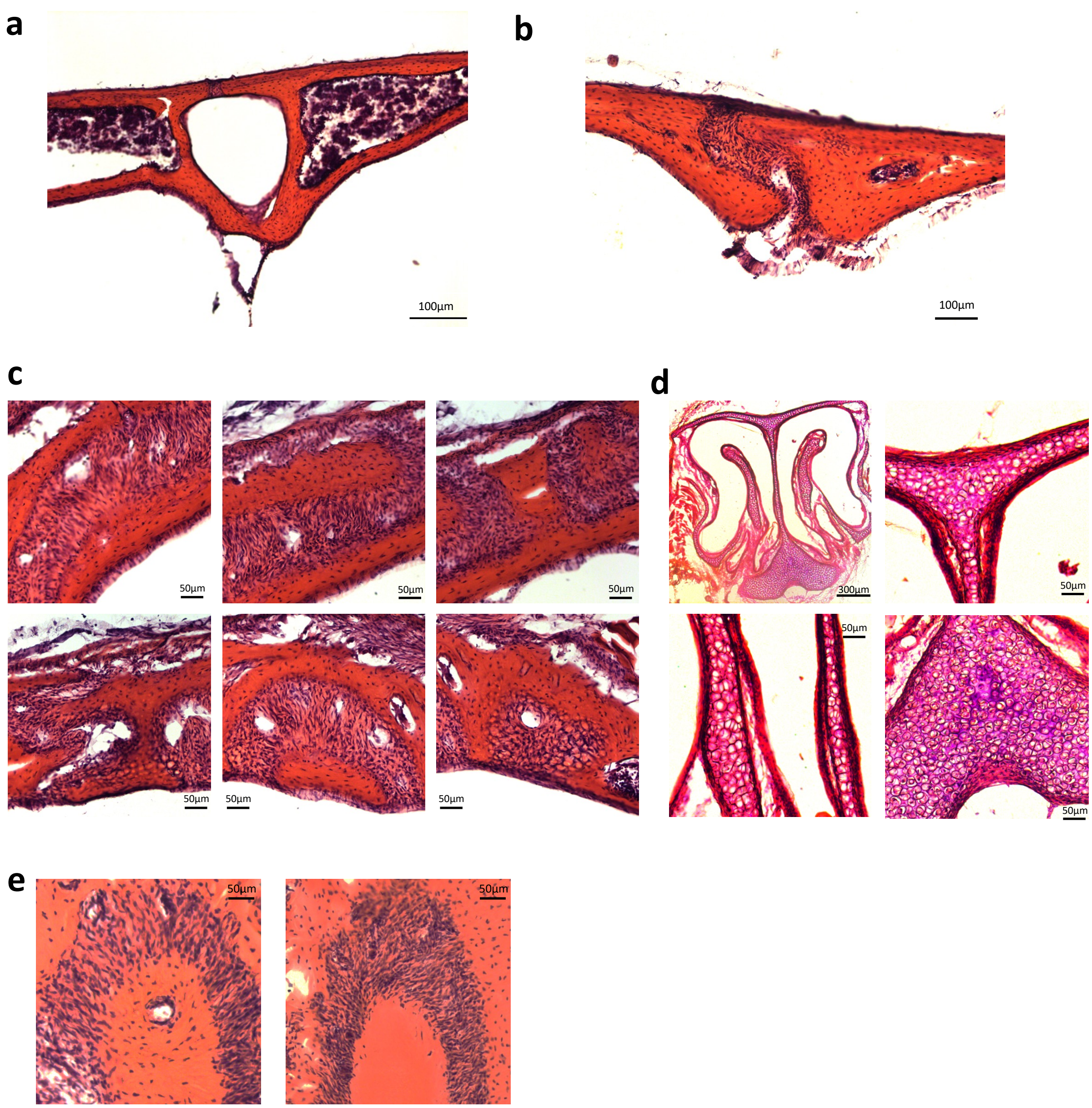

Supplementary Figure 4. H&E staining of mouse cranial bones 12 weeks after tamoxifen injection. (a-e) H&E-stained sections of **(a)** frontal bone, **(b)** parietal bone at the sagittal suture, **(c)** occipital bone, **(d)** nasal bone, and **(e)** mandible, all from β actin-Cre Rainbow mice (tamoxifen administered at P10, harvested 12 weeks later). **(d)** First panel, low magnification; remaining panels, high magnification. **(a-e)** Each panel corresponds to the same regions shown in confocal images in **Supplementary Figure 1a-e.**

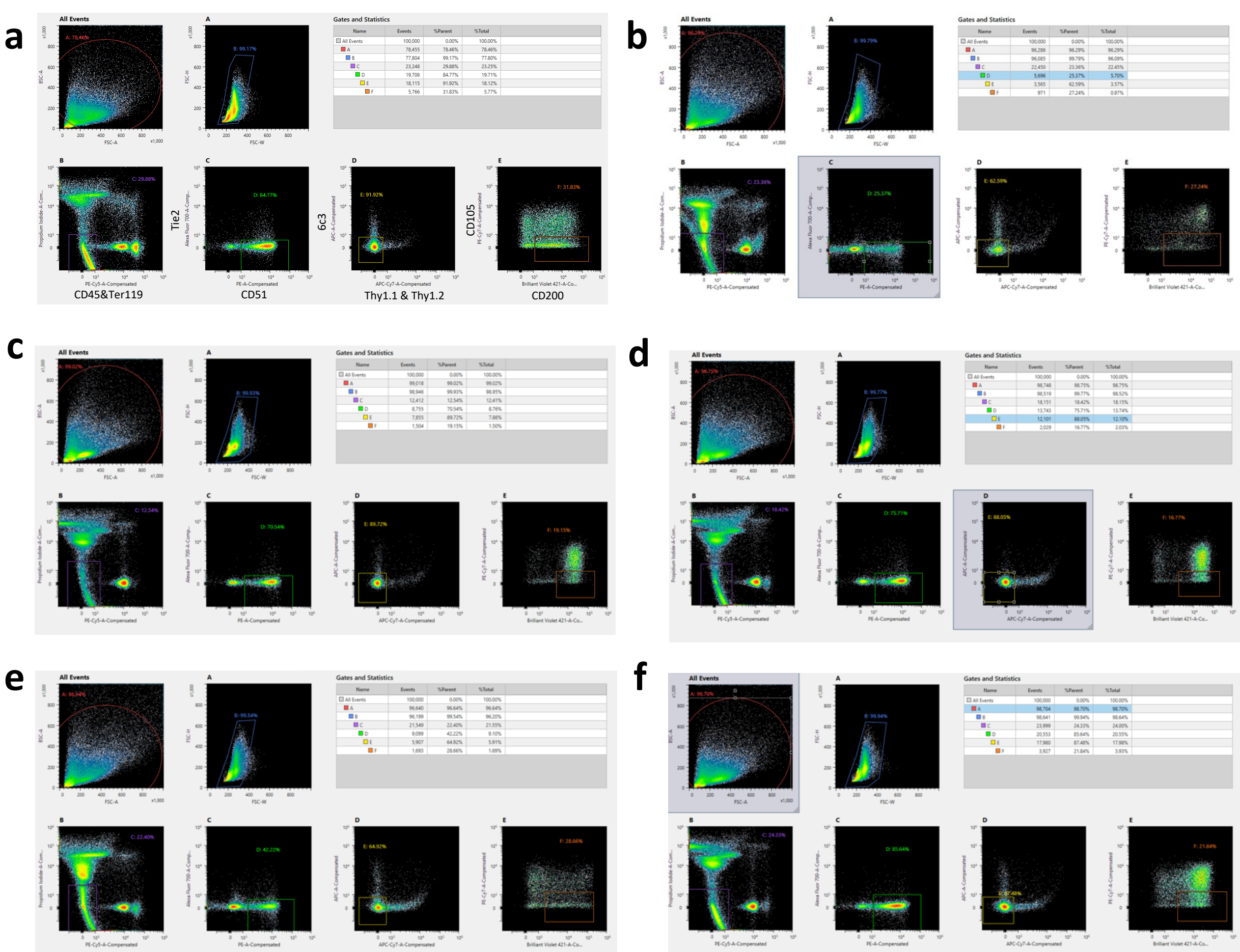

Supplementary Figure 5. Fluorescence activated cell sorting (FACS) gating strategy for quantifying SSCs.

(a-e) Representative FACS plots for sorting of cells from **(a)** femur, **(b)** frontal, **(c)** parietal, **(d)** occipital, **(e)** mandible, and **(f)** nasal bone, illustrating gating strategy used for quantification and isolation of SSCs. The process begins with the isolation of singlets to ensure individual cell analysis (red and blue gates). Subsequently, the CD45- Ter119- double-negative population (purple gate) is selected to exclude hematopoietic and erythroid cells. Within this subset, the Tie2- and CD51+ population (green gate) is selected to capture non-endothelial skeletal lineage cells. Gating on the Thy-6c3- population (yellow gate) excludes bone-lineage and stromal-lineage cells. Finally, within this select group, cells are segregated based on CD105 and CD200 expression: CD200+CD105- cells (orange gate) are sorted and identified as SSCs, while CD105+ cells are categorized as BCSPs.

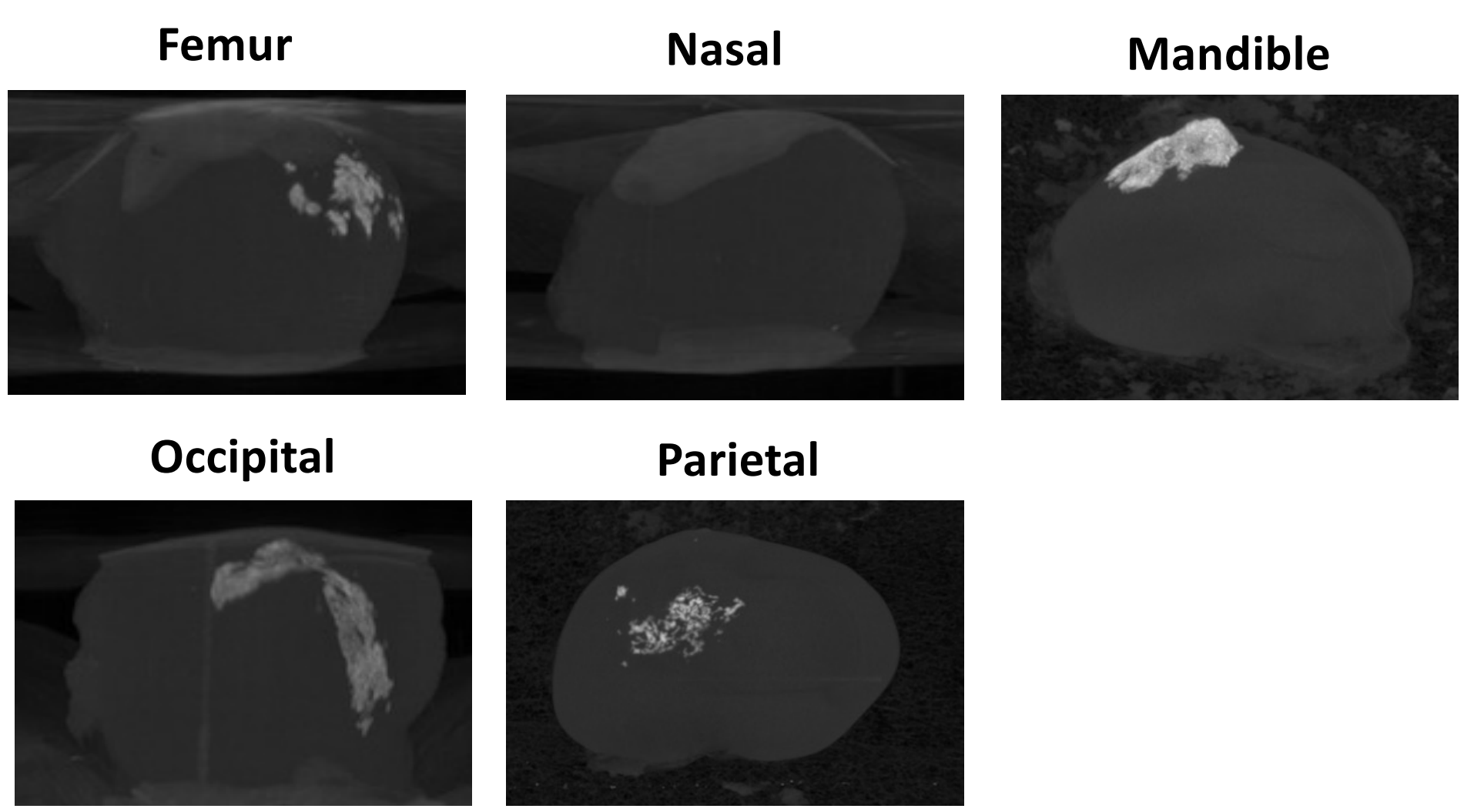

Supplementary Figure 6. Micro-computed tomography (CT) imaging of SSC-engrafted kidneys. Representative micro-CT images of kidneys 4 weeks following engraftment of SSCs from the indicated bones under the kidney capsule. White area represents bone deposition.

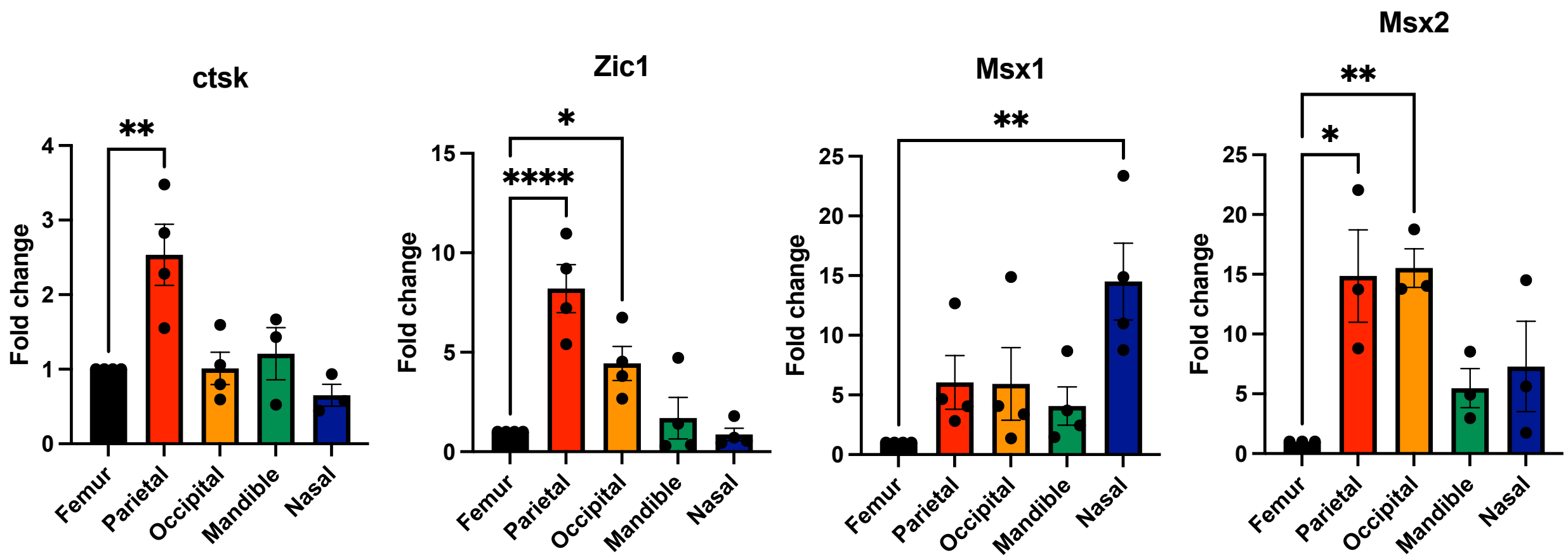

Supplementary Figure 7. Gene expression of previously identified cranial-SSC markers by qPCR. Results of qPCR assays for indicated genes (n=3-4 biological replicates per condition), showing fold difference normalized to expression levels in femur-SSCS for each replicate. For indicated comparisons, one-way ANOVA with Tukey Post-hoc was used, $*p < 0.05$, $**p < 0.01$, and $****p < 0.0001$.

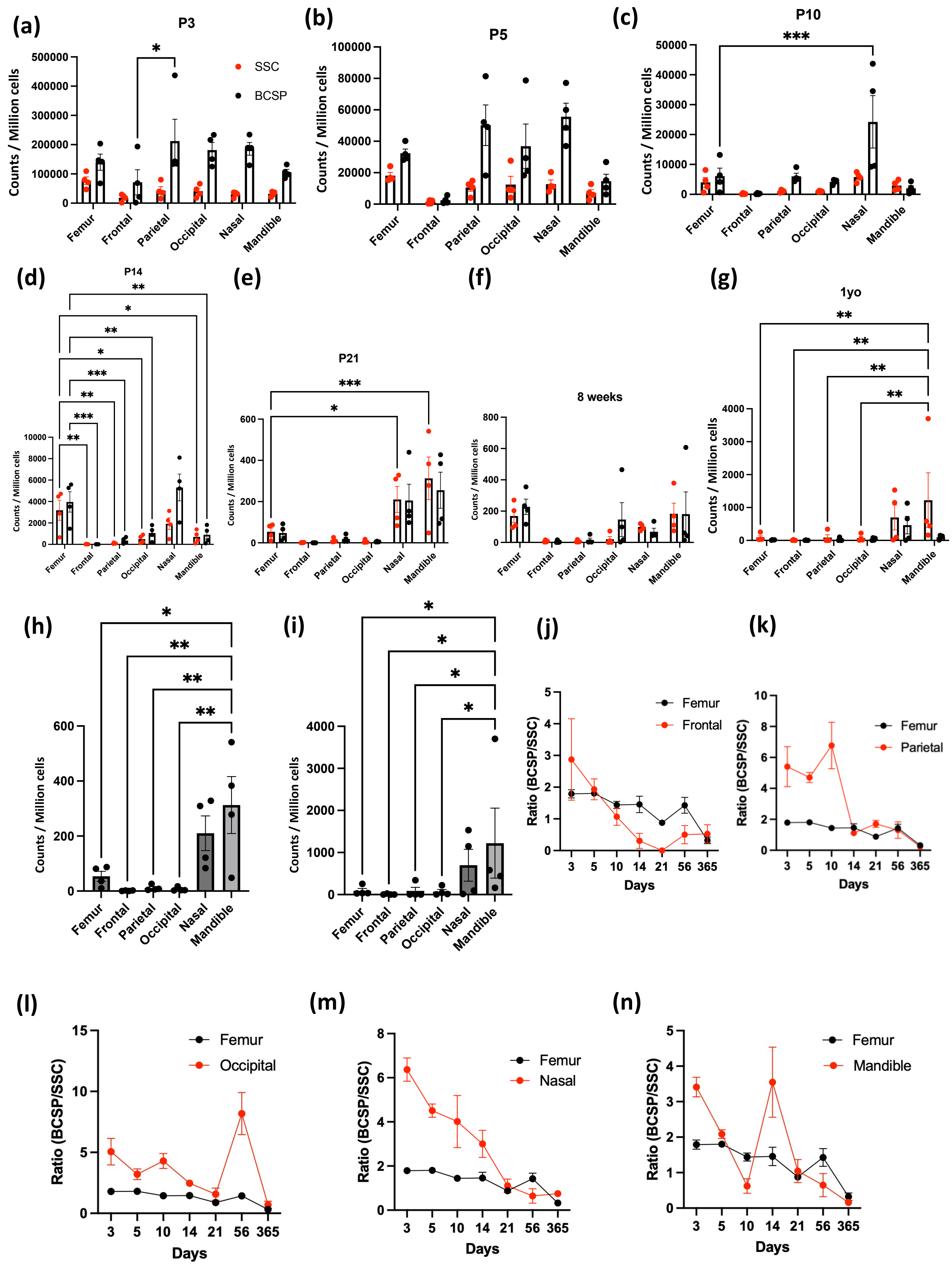
(a)
P3
SSC
BCSP
Counts / Million cells
Femur
Frontal
Parietal
Occipital
Nasal
Mandible
(b)
P5
(c)
P10
(d)
P14
(e)
P21
(f)
8 weeks
(g)
1yo
(h)
(i)
(j)
Femur
Frontal
Ratio (BCSP/SSC)
Days
(k)
Femur
Parietal
(l)
Femur
Occipital
(m)
Femur
Nasal
(n)
Femur
Mandible

Supplementary Figure 8. Prevalence of SSCs and BCSPs in cranial bones and femurs over time. (a-g) SSC and BCSP counts per million cells, based on FACS analysis of indicated bones, in **(a)** P3 mice, **(b)** P5 mice, **(c)** P10 mice, **(d)** P14 mice, **(e)** P21 mice, **(f)** 8-week-old mice, and **(g)** 1-year-old mice. **(h-i)** SSC counts per million cells from indicated bones in **(h)** P21 mice and **(i)** 1-year-old mice, showing comparisons in SSC prevalence between different bones. **(j-n)** Quantification of BCSP/SSC ratio over developmental time in femur (black) versus indicated cranial bones (red). **(a-i)** For indicated comparisons, *p < 0.05, **p < 0.01, and ***p < 0.001. n=4 biological replicates per condition.

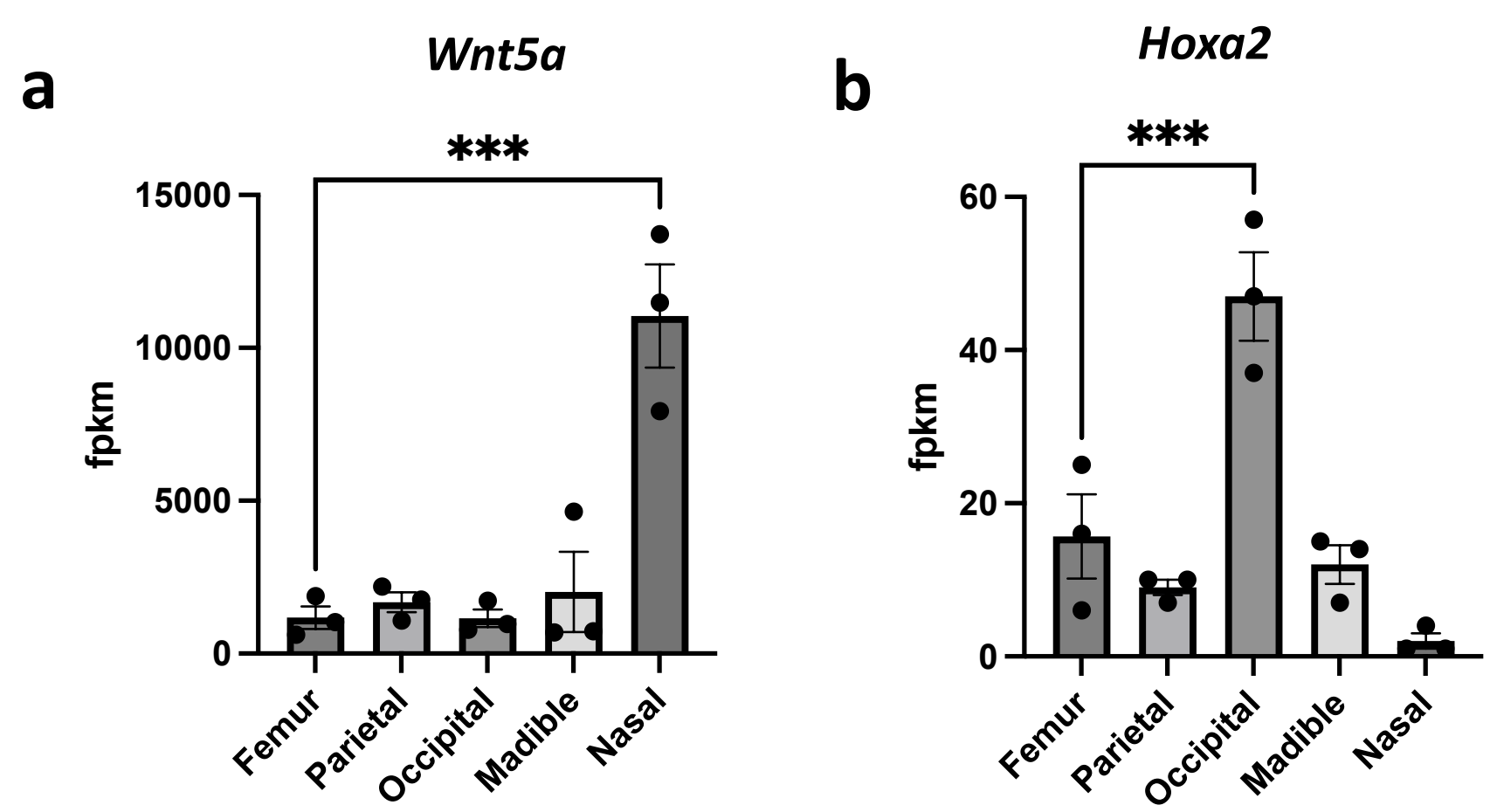

Supplementary Figure 9. Bulk RNA-sequencing analysis of *Wnt5a* and *Hoxa2* expression in SSCs. (a-b) Expression levels of **(a)** *Wnt5a* and **(b)** *Hoxa2* in SSCs isolated from indicated bones, based on bulk RNA-seq analysis. For indicated comparisons, one-way ANOVA with Tukey post-hoc was used, ***p < 0.001. n=3 biological replicates per condition.
