# Supplementary material for: Regional diversity of cranial skeletal stem cells governs bone morphogenetic protein-2 mediated bone regeneration
Source: Nat Commun. 2026 Aug 6;17:9479. doi: 10.1038/s41467-026-76286-w (PMC13542091; doi:10.1038/s41467-026-76286-w)
Supplement: Supplementary file 3 [file 41467_2026_76286_MOESM3_ESM.pdf]

nature portfolio

Peer Review File

Regional Diversity of Cranial Skeletal Stem Cells Governs Bone Morphogenetic Protein-2 Mediated Bone Regeneration

Corresponding Author: Dr Michael Longaker

This file contains all reviewer reports in order by version, followed by all author rebuttals in order by version.

A version of this paper was originally rejected for publication by Nature Communications, however that decision was reconsidered after appeal by the authors.

Version 0:

Reviewer comments:

Reviewer #1

(Remarks to the Author)
This manuscript from Takematsu, et al characterizes the activity of various immunophenotyped skeletal stromal cells from the craniofacial skeleton and their differential responses to BMP-2. There is an interesting start to this story, but it is currently insufficiently rigorous for reporting. It is not clear to this reviewer if there is insufficient data or if it is a presentation of data issue. Detailed comments below; nearly all comments of note have to do with lack of clarity on Ns analyzed in experiments, or lack of appropriate presentation of data.

Introduction:
Authors reference differential BMP-2 therapeutic efficiency in palate and maxillary as 50% and 90% in mandible. With only 2 references, it seems these differences could be attributable to any number of factors, but the next sentence discusses origin as a potential – linking the two thoughts would be good – i.e. noting differences in origin in palate, maxillary and mandible.

The intro also wraps up the story with the preliminary conclusion that sites of SSC/progenitor derivation show heterogeneity – something already also introduced as known. The concluding sentence or two in the introduction should clarify what this contribution provides in terms of new knowledge.

Reference 16 is referenced in the introduction (as introductory information, but the reference for this is not accessible). Perhaps this will be resolved for publication, but it is probably inappropriate to reference introductory material that is not peer-reviewed or at least accessible.

Figure 1: This reviewer is unsure if 1 month following a PN3 TMX injection is standard for assessing clonality; the authors reference one previous publication by the same authors. Perhaps this can be elaborated upon or reworded.
The quantification of Figure 1b must be shown in main text – one cannot state significance with a panel image. In this case, the higher number of ‘systematically higher number’ from nasal bone appears to be from one single N – is the N shown in the image from what appears to be an outlier – this would be of questionable rigor/reproducibility as the other two data points are much more similar to the rest of the bones. What do the 3 Ns represent? Three sections from a single animal, a single section, FOV from three animals? Three sections (and FOVs at same magnification) from three animals would likely be minimum necessary. Figure 1b is not even noted in figure legend – also ‘confocal image’ is used interchangeably with ‘magnified’ for this figure. Scale bars and legend clarification is needed.

Figure 2b: notable more Ns shown for this data. Explanation of what each dot represents needed to assess rigor.
Figure 2d and 2e – again, what does each dot on the quantification represent? Number of animals (biological replicates) and technical replicates needed and should be shown and legends include these details on rigor.
The differential outcomes for this work is very interesting, but highlights the necessity of a rigorous and reproducible assessment. Results from osteo- and chondro- differentiation assays can have high variability. It is unclear if the results have been reproduced sufficiently for publication.
Ns used in Figure 2g and h are not shown and should be. Presumably RNAseq was performed on more than a single

replicate – heat map should be inclusive of all data, not averaged (or if only one sample, Ns are insufficient).

Figure 3-5 – The same issues exist for this data - again Ns and visualization of data needs to be demonstrated. Clarification on biological and technical replicates, RNAseq presentation of biological Ns (or insufficient number if just one).

Figure 4 – It is insufficient level of examination to conclude the involvement of Wnt signaling from bulk RNAseq results. Validation of this interesting set of results is required. This figure should be dropped or pursued (or put in supplemental) – one cannot make conclusions from it.

Reviewer #2

(Remarks to the Author)
The repair of cranioskeletal tissues is a significant clinical problem in relation to disease and injury. Although mesenchymal stem cells were traditional considered the progenitors of skeletal tissue, their differentiation capacity is quite broad and not skeleton specific. The authors of this study previously identified skeletal stem cells (SSC) that exclusively differentiate into skeletal lineage cells such as bone, cartilage and stromal cells, and through CSF antagonist/BMP2 it’s been shown SSCs exhibit enhanced activity in repairing fractures in the elderly.

But different bones in different parts of the body have different mesoderm or neural crest cell origins and while most studies have focused on mesoderm derived long bones, which are formed via endochondral ossification, few have investigated the neural crest or mesoderm derived intramembranous bones of the skull. The authors therefore ask the question of whether cranial SSCs possess unique and functional attributes.

In this study the authors initially use B-actin-Cre rainbow mice to perform lineage tracing and clonal analyses within different bones, then they immunosort SSCs, demonstrate they can be successfully differentiated into bone or cartilage derivatives, evaluate their transcriptional profile, and response to BMP signaling all of which are critical for consideration in surgical skeletal repair, and may avoid some of the complications of autografts from the iliac crest.

While it is important to optimize treatment in various areas of cranial bone repair, and the findings are interesting, they are incomplete and the functional criteria the authors want to establish are not met in an acceptable in vivo model. The key limitation is the absence of in vivo functional testing in which the SSCs are used in a critical skull bone injury model.

Clonal analyses were performed from P3-P33 (one month) and the nasal tissue was found to contain the highest number of clones, followed by the mandible, but the other cranial bones did not demonstrate high clonality. But what this means isn’t clear. How to the authors define high versus low clonality. What’s the metric? I did not see any quantification, nor any rationale or articulation of what the clonality represents, or its connection to SSCs, other than just an observation. Is there any physiological or functional difference? As the authors show later, the gene expression profiles across the different bone samples are very different, and simple differences in cell adhesion could make all the difference in clonality score. Similarly, after sorting SSCs from different cranial bones, nasal derived SSCs had the highest colony forming ability, while in contrast it was low for the frontal bone. But the significance of this isn’t made clear.

One of the challenges to the data is that there’s no real pattern or consistency among the different bones, in terms of their clonal distribution or colony forming ability, except being highest for the nasal tissue, and the fact that nasal-SSCs do not have osteogenic capacity in the (cell culture and kidney capsule) differentiation assays performed, whereas all the different bone derived SSCs have chondrogenic ability.

The osteogenic versus chondrogenic differentiation capacity may be associated with expression of bone anabolic or remodeling gene signatures which were high in the mandible and femur, but low in the nasal SSCs. But this then raises the question of why there are differences in the efficacy of grafts of mandibular bone versus femur bone in critical size bone defect repair.

The data comparing the ratio of SSCs and Bone Cartilage Stromal Progenitors from P3-1 year is interesting but also somewhat confounding. At least in the skull, the first 2-3 weeks of postnatal development coincide with the highest degree of craniofacial growth so isn’t it counterintuitive that SSC’s ramp up after this period? The ratio peak at 2 weeks may therefore be more relevant. I do however think it is important that the number of SSCs is higher in 1 year old mice in the mandible than the femur, when considering the source of bone for a cranial skeletal defect repair in humans or why mandible holes repair faster than femur holes. But there are many other reasons why this might be the case considering the adult functions and physiological forces and stresses that each of these bone are affected or influenced by in an adult. Nonetheless it is interesting that the SSC dynamics are different between cranial bones and long bones and this can help determine where too source the cells for the most efficacious autologous grafts.

The bulk RNA sequencing of SSCs isolated from each bone has the potential to reveal important differences, but too many assumptions are made about the presence of individual Wnt factors or stemness markers or their downstream genes, as well

as cytokines and ctsk. This section describing every assay and parameter and bone was really confusing. There was no coherent message connecting the dots, but more importantly the data is primarily correlative and speculative with no confirmation of function.

I have similar concerns with the experiments involving different populations of SSCs and their responses to BMP signaling. There's a lot of variation which may be possible to tie to intrinsic gene expression profiles, but again the interpretations are quite speculative and a demonstration of the mechanism underlying the differences is not provided.

Overall, it's interesting that the nasal tissue has the highest number of SSCs and the greatest response to BMP2, but their differentiation is limited to chondrogenesis in the assays used. The full potency was not tested under other possible differentiation conditions.

I think it is incorrect to state for example on line 289 that "we have uncovered the genetic and functional diversity within cranial SSCs which critically influences the outcomes of bone regenerative therapies" because this was never functionally tested. Testing whether the different SSCs truly have distinct repair capacities in critical sized bone defects would seem to be an absolutely essential part of the study to validate the claims.

Reviewer #3

(Remarks to the Author)
The Diversity in Cranial Skeletal Stem Cells Regulates Bone Regeneration with Bone Morphogenetic Proteins

In this study, Takematsu et al. (2024) report the diversity in cranial skeletal stem cells (SSCs) regulates bone regeneration with bone morphogenetic proteins. They evaluated the clonality of osteogenic lineage cells in the cranial bones by using Actb-CreERT;Rainbow mouse. They next isolate SSCs from each bone (femur, frontal, parietal, occipital, nasal, and mandible) and compared the clonal capacity, the differentiation capacity, and the gene expression property. They demonstrated that higher osteogenic property in the SSCs isolated from femur, occipital and mandible, while cells from nasal cells showed high chondrogenic property. They also evaluated the percentage of SSCs, osteogenic cell, chondrogenic cells, and bone cartilage stromal progenitors (BCSPs) in the isolated cells from each bone. From DEG analysis of RNA sequence, authors presented marker genes specific for each bone. Finally, they tested the effects of bone morphogenetic protein (BMP)-2 on induction osteogenic capacity of isolated cells.
The viewpoints of this study are valuable, and they identified different properties of osteogenic lineage cells in each cranial bone. However, results are still descriptive and lacking molecular functional analyses explaining each cellar properties and gene expression. The study could be very interesting but it is too premature at this point. The manuscript is too unfriendly to the readers.

I also describe some points which require to improve this study.

1. From which stage of mice did the authors isolated cells for in vitro study? The property of the cells must be different between stages. I could not find it but even if the authors describe it in the text, they should mention it in the result section.

2. It is difficult to understanding the tissue structure from Figure 1b-j. Haematoxylin-Eosin staining should be included with fluorescent images. Because it is very important to understand original tissues to consider the properties of isolated cells.

3. It is difficult to understand the meaning of the figures. Appropriate labelling is required. It is not reader friendly.

4. Nasal bone has large cartilaginous region. Authors may also show the cartilaginous region of nasal bone in Figure 1b. They tested the chondrogenic properties of SSCs, but it is considered that isolated cells from the nasal bone were chondro-progenitors rather than SSCs.

5. Authors should describe material and method or experimental strategies more kindly, for example, age and gender of used mice, tissue isolation procedures, and detail information of the antibodies.

6. Authors define the SSCs properties from each bone, but they also should show which part of the tissue they originally isolated cells from.

Version 1:

Reviewer comments:

Reviewer #2

(Remarks to the Author)

The authors should be commended for their efforts to revise their manuscript in response to the reviewers' concerns. I especially appreciate the addition of the critical size calvarial bone defect transplantation repair assay. This was not a trivial experiment but the new data does provide important in vivo support for the authors' interpretations and conclusions. Removing some of the transcriptomic data together with elimination of the speculation in the previous submission has strengthened and tightened the study.

My remaining questions are comparatively minor but clarification can further strengthen the study
For the benefit of the readers, especially non-experts, it would be helpful to indicate the plane of section in the clonal and other skull analyses.

The clonality score is still somewhat confusing because, although clones were noted, the methodology used doesn't convey a complete evaluation of the total number of clones. How does one ensure clones were not missed in the sea of different colors which to my eye can make it difficult to identify all the clones in the first place. Also, can the authors better justify β actin-CreERT/Rainbow instead of maybe Sox10-CreERT as an indelible label of SSCs?

Line 60 reports the Actb-CreERT (βactin-CreERT/Rainbow) mice, but the methods refer to ActinCreER-R26VT2/GK3. The nomenclature for the mice used should be consistent throughout the manuscript.
Why do the authors think there were no clones in the P13.5W neural crest cell derived frontal bone?

If they are progenitor cells instead of SSCs, is this a limitation with the βactin-CreERT/Rainbow approach or are those original neural crest cells replaced by some other non βactin-CreERT cell type?

The small number of SSCs in the frontal bone and their low colony forming assay ability was surprising – any explanation – are they NCC derived or rapidly replaced. Is it the bone of maximum growth in the skull during the post -natal period analysed?

The data is a bit confusing because at P21, the nasal bones have comparatively more SSCs than other calvarial bone regions. And yet, the that nasal bone doesn't have osteogenic capacity but primarily forms cartilage under renal capsule assay? Can the authors account for this or does it come down to Wnt5-Bmp2 competing expression?

Why do immunophenotypically defined SSCs from different cranial regions display distinct functional activities – are there any known differences in the physiology of occipital versus parietal versus frontal versus nasal bone (apart from progenitor cell origin). The authors allude to a difference in the mandible which may have a physiologcal-mechanical underpinning, but what about for the calvarial bones? Or is it simply technical and and experimental limitation.

The higher frequency of SSCs in the mandible seems to me that it could underpin the general success of mandibular distraction as a surgical repair technique for hypoplastic mandibles.

The BCSP/SSC ratio is an intriguing measure for active growth but how do the authors correlate that with the relative paucity of SSCs in the nasal bones compared to other cranial bones and what can the authors say about the distinct growth patterns of the nasal bone relative to other bones that could account for this.

It's interesting that nasal SSCs exhibited the strongest response to BMP-2. Maybe the nasal SSCs are a bit of an enigma because, they produce the highest frequency of clones, yet in the critical size calvarial bone defect assay the nasal SSCs predominantly formed cartilage unless treated with BMP2.

If cranial-SSCs decode BMP-2 cues in a region-dependent manner, what does the field now do with this information when it comes to calvarial or other cranioskeletal repair? Is like for like repair the recommendation wherever possible? Concluding the manuscript with a recommendation based on the authors data would make the manuscript more complete.

Reviewer #3

(Remarks to the Author)
The manuscript entitled "The Diversity in Cranial Skeletal Stem Cells Regulates Bone Regeneration with Bone Morphogenetic Proteins" has been revised.

The results in the manuscript indicate the importance to optimize of BMP-2–based bone regeneraton therapies.

The first part that analyzed the SSC status during mouse postnatal became clearer to suggest that the ratio of SSC number and BCSP number could be explained by the requirement of Bone Cartilage Stromal Progenitors.

In the introduction, when the proper name of BCSC is not written when it first appears.
It may not be the main interference of the manuscript but in Fig.3, the authors write that the mandible generally heals more rapidly than the femur. They attribute the finding to the higher number of mandibular SSC. At the beginning, the authors find the main SSC localization in the mandible is the periodontal ligament. Does it mean the healing is better when mandibular body was broken compared to when that ramus of the mandible is broken? Since dental mesenchyme could be different from that to form mandibular bone since the periodontal ligament is mainly derived from the dental follicle that is dental

mesenchyme. The dental follicle forms alveolar bone but not mandibular bone during development. The explanation seems to be required.

The authors did not analyze the frontal SSC in Fig. 2 since they could not obtain a good number of cells, however, Fig.3 shows that the rate of the frontal SSC is more or less the same.
Were Fig.2g and h results obtained from SSC or after differentiation induction?
In Fig. 4, the authors demonstrate that Wnt5a is highly expressed in the nasal SSC, that is the reason of different response to BMP2 from the femur SSC. However, BMP2 addition can induce calcification in the nasal SSC although addition of Box5 enhanced the effect. Are there any possibilities that BMP2 treatment affect Wnt signaling?
Fig. 5 is a chaos. It is hard to recognize the contribution of each SSC in the images. For instance, in Fig. 5J Nasal SSC appears to form the cartilage like structure without BMP2, however, with BMP2 newly formed bone osteocytes seem to be negative for GFP. Didn't nasal SSC contribute to bone formation? In this case, since there are many players, it is hard to get the simple answer. However, the authors carried out a lot of experiments, the readers would like to have as simple answer as possible. One more concern is that the authors are looking at the results. After bone defect formation, the healing process starts at least by day 7 and it might be appropriate to observe earlier stage. In addition, all cell types were implanted to the parietal defect. It is probably the best place to carry out stable experiments, however, It is possible that environmental factors implanted into the parietal bone might have some effects on the results.

Is Fig6 required? There must be a better way to describe the authors' observation.

Version 2:

Reviewer comments:

Reviewer #2

(Remarks to the Author)
The authors have satisfactorily addressed my primary concerns and clarified a number of issues which has improved their paper.

I am however still hung up on the description of the Cre line, because some of the authors of this paper have used the same clonal labeling strategy in previous papers, most notably Rinkevitch et al., 2015 where the line was first denoted as ActinCreER (and a gift from Liqun Lo) to generate ActinCreER; R26VT2/GK3 mice, and again in Zhao et al 2023 where the mice were denoted as ActinCreER/R26VT2/GK3 Rainbow reporter mice.

If the authors now call this line B-ActinCreERT then it becomes near impossible and confusing to trace the origins of original Cre line used in their study and this is important for accuracy and context. The original tamoxifen inducible B-ActinCre (B-ActinCreERT) line was generated by Guo, Yang and Lobe (https://doi.org/10.1002/gene.10021)

In the interests of the field and to be consistent with prior literature (especially papers published by some of the same authors) please use the original nomenclature throughout the manuscript so that the field clearly understands what Cre line was used and what was the original source of the Cre line used (with citations). This is critical for rigor and reproducibility.

Reviewer #3

(Remarks to the Author)

The manuscript has been revised and now easier to understand the content. However, it still has the points to be proved. Some Figures do not show the temporal sequence. Fig.1 f and g should be swapped. In Fig. 5, l and m should be located at least before b.
In Sup Fig.5, the authors describe the isolation of SSC from parietal, occipital, mandible, and nasal bone, but not frontal. In Fig. 2 the result of colony forming assay involves frontal cells. It is therefore confusing if the authors adopted the same method to isolate SSC through the investigation Later in Fig. 3 there are results with frontal cells. It would be important to make the text easy to understand.

In Sup Fig. 8, are all y-axes the same, Counts/ Million cell?

In Fig.5a, GFP+/Sox9+ cells indicated by arrows do not show chondrogenic morphology. Since GFP+ cells were implanted, their characters may change, however, they do not look like chondrocytes compared with arrows in 9d. The results are after 4 weeks so it is hard to consider the cells are in differentiation process.

P.25, the authors describe the result with mandibular SSC and mention that mandibular bone occurs by intramembranous ossification, however, the parietal bone forms by intramembranous ossification. It seems that parietal SSC seems to show the same tendency.

In Fig. 6, the position of Parietal and Femur circles should be considered. Although in vitro cartilage induction indicated that Femur has mot capacity, Parietal demonstrate more Sox9+GFP+ cells in Fig. 5. In addition, with BMP2 treatment, in vivo experiments exhibited suppression of cartilage formation overall. Therefore, it seems not right to see that most of arrows move to top right direction.

Line 584: The use of the parietal bone defect is understandable, however, “parietal regions may provide a more permissive environment that supports broader lineage potential” is the authors’ expectation without trying other bone defects.

Line 594: Is high clonality generally associated with the differentiation bias of the isolated SSC or high proliferation?

Line 617: How does airflow produce mechanical stimulation?

Version 3:

Reviewer comments:

Reviewer #3

(Remarks to the Author)
The manuscript has been revised properly.

Line: 344-345: Mouse weaning does not occur at P14. The authors refer the paper that describe "In commercial breeding facilities, weaning occurs usually in the third week after birth. If dams are checked for parturition twice weekly—which according to personal communication with various commercial breeders seems to be common practice—newly weaned animals may range in age from 17 to 25 days of age.". The data for BCSP/SSC ratio in mandible must have other reasons. At p14, the pups are still drinking milk.

Open Access This Peer Review File is licensed under a Creative Commons Attribution 4.0 International License, which permits use, sharing, adaptation, distribution and reproduction in any medium or format, as long as you give appropriate credit to the original author(s) and the source, provide a link to the Creative Commons license, and indicate if changes were made.
In cases where reviewers are anonymous, credit should be given to 'Anonymous Referee' and the source.
The images or other third party material in this Peer Review File are included in the article’s Creative Commons license, unless indicated otherwise in a credit line to the material. If material is not included in the article’s Creative Commons license and your intended use is not permitted by statutory regulation or exceeds the permitted use, you will need to obtain permission directly from the copyright holder.
To view a copy of this license, visit https://creativecommons.org/licenses/by/4.0/

Tittle: The Diversity in Cranial Skeletal Stem Cells Regulates Bone Regeneration with Bone Morphogenetic Proteins

Manuscript number: NCOMMS-24-25449

Table of Contents

Reviewer comments	Page Number
**Reviewer 1**	
Comment 1	Page 2
Comments 2-4	Page 3
Comments 5-8	Page 4
Comments 9-13	Page 5
**Reviewer 2**	
Comment 1	Page 7-9
Comment 2	Page 9-10
Comment 3	Page 10
Comments 4-5	Page 11
Comment 6	Page 12
Comments 7-8	Page 13
Comment 9	Page 14
**Reviewer 3**	
Comment 1	Page 15
Comments 2-4	Page 16
Comment 5	Page 17

Reviewer #1 (Remarks to the Author)

This manuscript from Takematsu, et al characterizes the activity of various immunophenotyped skeletal stromal cells from the craniofacial skeleton and their differential responses to BMP-2. There is an interesting start to this story, but it is currently insufficiently rigorous for reporting. It is not clear to this reviewer if there is insufficient data or if it is a presentation of data issue. Detailed comments below; nearly all comments of note have to do with lack of clarity on Ns analyzed in experiments, or lack of appropriate presentation of data.

We appreciate the reviewer's comments and interest in this story. We have now added sufficient biological replicates and clearly stated sample size in all figure legends, and we believe the clarity and rigor of the study are much improved by our revisions in response to reviewer comments, as outlined in detail below.

1. Introduction:
Authors reference differential BMP-2 therapeutic efficiency in palate and maxillary as 50% and 90% in mandible. With only 2 references, it seems these differences could be attributable to any number of factors, but the next sentence discusses origin as a potential – linking the two thoughts would be good – i.e. noting differences in origin in palate, maxillary and mandible.

We thank the reviewer for pointing this out. The results of our study do highlight a likely stem cell basis for differential BMP-2 therapeutic efficiency at these different sites, based on our finding of differential BMP-2 responsiveness by resident SSCs in different regions, and the known central role of SSCs in bone healing/regeneration. However, we agree that the clinically observed healing differences between sites could also have contributions from many different factors, and it is important to discuss this complexity in greater detail in the manuscript.

We have now provided additional citations to support the bone healing differences in the maxilla versus mandible. Additionally, we have expanded in both the Introduction and the Discussion sections (pgs. 5 and 31 of the revised manuscript, respectively) on the potential contribution of additional factors to regional healing differences:

> *Pg. 5:* Lack of dose standardization may underlie the varied outcomes of clinical trials for BMP-2-based cranial bone regeneration depending on anatomical site. For example, reported success rates of BMP-2 for promoting bone healing have been as low as 50% in the palate or maxillary bone, compared to over 90% in the mandible[18-21]. Certainly, many factors could contribute to these disparate outcomes—for instance, differences in bone density[22,23] and mechanical forces[20,24]. However, given the central role of stem cells in bone regeneration[8,25] and our findings of anatomical heterogeneity of cranial SSC phenotypes, we hypothesized that variability in treatment outcomes is attributable to SSCs' differential responsiveness to BMP signaling.

Pg. 31: There are likely additional factors beyond SSC heterogeneity that impact differential regeneration between bone sites. For instance, the observation that mandibular bone generally exhibits superior regenerative capacity compared with the femur likely results from multiple factors beyond SSC behavior—including region-specific vascularization, marrow composition, and mechanical loading. By thoroughly delineating the intrinsic diversity and BMP-2 responsiveness of SSCs, our study provides a framework for future investigations that integrate these other factors—such as biomechanical and vascular contexts—to fully explain and predict site-specific regenerative outcomes.

2. The intro also wraps up the story with the preliminary conclusion that sites of SSC/progenitor derivation show heterogeneity – something already also introduced as known. The concluding sentence or two in the introduction should clarify what this contribution provides in terms of new knowledge.

We appreciate the reviewer's comment. Now we conclude the introduction with a few sentences that explicitly states what is new—the causal link between regional SSC identity and BMP-2 response.

Pg.5. While SSC heterogeneity is not a new concept, its functional relevance to BMP-dependent bone repair was previously undefined. Our study bridges this gap by demonstrating that cranial SSCs from different anatomical origins interpret BMP-2 stimulation through distinct lineage programs, uncovering a positional code that governs regenerative behavior. These findings reframe the concept of SSC diversity as a directly clinically relevant determinant of therapeutic response, thus paving the way for precision-targeted BMP treatment for cranial bone regeneration.

3. Reference 16 is referenced in the introduction (as introductory information, but the reference for this is not accessible). Perhaps this will be resolved for publication, but it is probably inappropriate to reference introductory material that is not peer-reviewed or at least accessible.
We agree with this concern and have removed this citation.

4. Figure 1: This reviewer is unsure if 1 month following a PN3 TMX injection is standard for assessing clonality; the authors reference one previous publication by the same authors. Perhaps this can be elaborated upon or reworded.
We appreciate the reviewer's query regarding justification for this choice of time point. We do believe that one month is the most appropriate time point to analyze stem cells' clonal expansion, based on prior studies performed by our and other labs. Newton et al. (A radical switch in clonality reveals a stem cell niche in the epiphyseal growth plate, *Nature,* 2019) demonstrated that stable clones originating from long-term progenitors are observed one month after induction. Cheng et al (Growth Plate Skeletal Stem Cells and Their Actions Within the Stem Cell Niche, International Journal of Molecular Sciences, 2025) also demonstrated clonal expansion of skeletal stem cell at the growth plate at P36, 30 days after tamoxifen induction at P6. Finally, though there is, to our knowledge, no prior study that specifically analyzes clonality using the Rainbow system

in craniofacial bone, Li et al. (Cranial Suture Mesenchymal Stem Cells: Insights and Advances, Biomolecules, 2021) demonstrated in a cranial suture that pronounced expansion of skeletal stem cells is observed at 4 weeks post-injury. We believe this body of research supports the choice of 1 month as a standard timepoint for assessing clonality in bone and have now incorporated these additional citations into the manuscript on pg. 6:

> Pg.6. One month after the initial tamoxifen pulse is the standardized time point for analyzing clonality to indicate the presence of progenitor cells[4,8,28-30].

5. The quantification of Figure 1b must be shown in main text – one cannot state significance with a panel image. In this case, the higher number of 'systematically higher number' from nasal bone appears to be from one single N – is the N shown in the image from what appears to be an outlier – this would be of questionable rigor/reproducibility as the other two data points are much more similar to the rest of the bones.

We apologize for insufficiently documenting this quantification in the original manuscript; the relevant quantification, with information on statistical significance of comparisons, is now fully shown in Fig.1g. (As stated in figure legend, * corresponds to $p < 0.05$; ** $p < 0.01$; *** $p < 0.001$; and **** $p < 0.0001$.) We analyzed n=4-6 mice per condition and took several images from each region and mouse for quantification (as now indicated in the figure legend). Frequency is now normalized to clone number / mm^2.

6. What do the 3 Ns represent? Three sections from a single animal, a single section, FOV from three animals?

N represents the number of biological replicates (individual mice) (now n=3-6 for all experiments and with explanation of sample size detailed in all legends). We took several images per slide for all quantification.

7. Three sections (and FOVs at same magnification) from three animals would likely be minimum necessary. Figure 1b is not even noted in figure legend – also 'confocal image' is used interchangeably with 'magnified' for this figure. Scale bars and legend clarification is needed.

As per the response to comment 6 above, we have added more mice and slides/images for quantification and have detailed the sample size/explanation of replicates in figure legends. We have also added scale bars and clarified the figure legends.

8. Figure 2b: notable more Ns shown for this data. Explanation of what each dot represents needed to assess rigor.

Here, n=2-3 biological replicates with 5-9 technical replicates per condition (each corresponding to a dot); we have updated the figure legend for clarification and rigor.

9. Figure 2d and 2e – again, what does each dot on the quantification represent? Number of animals (biological replicates) and technical replicates needed and should be shown and legends include these details on rigor.

For Fig. 2d, n=3 biological replicates / 9 technical replicates in total (corresponding to each dot); for Fig.2e, n=3 biological replicates with 7-9 technical replicates in total. We have updated the figure legend accordingly.

10. The differential outcomes for this work is very interesting, but highlights the necessity of a rigorous and reproducible assessment. Results from osteo- and chondro-differentiation assays can have high variability. It is unclear if the results have been reproduced sufficiently for publication.

As mentioned above, we have repeated the *in vitro* and *in vivo* assays to ensure reproducibility and sufficient rigor, and we now clearly explain how many biological/technical replicates were done for each assay.

11. Ns used in Figure 2g and h are not shown and should be. Presumably RNAseq was performed on more than a single replicate – heat map should be inclusive of all data, not averaged (or if only one sample, Ns are insufficient).

We appreciate the reviewer's concerns regarding insufficient replicates and as such have removed the majority of this RNA-seq data.

12. Figure 3-5 – The same issues exist for this data - again Ns and visualization of data needs to be demonstrated. Clarification on biological and technical replicates, RNAseq presentation of biological Ns (or insufficient number if just one).

As above, we have now removed the majority of the RNAseq data.

13. Figure 4 – It is insufficient level of examination to conclude the involvement of Wnt signaling from bulk RNAseq results. Validation of this interesting set of results is required. This figure should be dropped or pursued (or put in supplemental) – one cannot make conclusions from it.

We appreciate and agree with this concern regarding our prior evidence being insufficient to support conclusions about the role of Wnt signaling. As above, we have now removed the majority of the RNAseq data with the exception of examination of two specific genes (in Supplementary Fig. 9). Instead, we have added qPCR analysis to examine expression levels of specific key osteogenic and chondrogenic genes (n=3-4 biological replicates; Fig. 2g, h and Fig. 4e-i). We also used qPCR to assess expression of *Wnt5a*, which we identified as significantly enriched in nasal-SSCs (Fig. 4j). To more robustly establish a functional role for Wnt signaling, we then tested the effects of inhibition of Wnt5a with Box5 (a potent and specific Wnt5a antagonist) in an osteogenesis assay. Box5a treatment promoted osteogenesis of nasal-SSCs under

BMP-2 stimulation (Fig.4k-m). These results are delineated in the revised manuscript on pgs. 20-21:

> Pg.20-21. We next investigated the mechanisms underlying these divergent osteogenic capacities, focusing on the striking BMP-2 responsiveness we observed in nasal-SSCs. Bulk RNA-sequencing revealed that Wnt5a expression was significantly higher in nasal-SSCs compared with femur-SSCs (Supplementary Fig. 9a), which was confirmed by qPCR showing nearly 20-fold higher Wnt5a transcript levels in nasal- versus femur-SSCs (Fig. 4j). Based on previous study showing that non-canonical Wnt signaling can suppress osteogenesis in cranial stem cells, we hypothesized that elevated Wnt5a suppresses osteogenic differentiation through enhanced non-canonical Wnt signaling. To test this, we assessed osteogenesis in femur- and nasal-SSCs treated with Box5 (100 µM), a selective Wnt5a antagonist, in the presence or absence of BMP-2 (100 ng/mL) (Fig. 4k). As expected, BMP-2 treatment alone promoted mineralization in both femur- and nasal-SSCs (Fig. 4l-m, first and second bars). However, when Box5 was administered without BMP-2, neither femur- nor nasal-SSCs demonstrated substantial enhancement in mineral deposition relative to untreated controls (Fig. 4l-m). In contrast, Box5 treatment combined with BMP-2 resulted in increased osteogenesis compared with BMP-2 alone, with a modest improvement observed in femur-derived SSCs and a significantly greater enhancement in nasal-SSCs (Fig. 4l-m). These data refine our initial model and indicate that Wnt5a primarily acts to restrain BMP-responsive osteogenesis rather than suppress basal osteogenic differentiation. In femur-SSCs, low basal Wnt5a expression imposes minimal inhibition, resulting in only a slight enhancement when Box5 is added to BMP-2. In contrast, the high Wnt5a expression in nasal-SSCs creates a strong inhibitory threshold that can be relieved when both BMP-2 stimulation and Wnt5a blockade are present. Thus, the reduced osteogenic potential of nasal SSCs compared with femur-derived SSCs under BMP-2 stimulation (Fig. 4k) appears to be driven, at least in part, by elevated non-canonical Wnt signaling mediated by Wnt5a, which restricts their responsiveness to osteoinductive cues such as BMP-2.

Reviewer #2 (Remarks to the Author)

The repair of cranioskeletal tissues is a significant clinical problem in relation to disease and injury. Although mesenchymal stem cells were traditional considered the progenitors of skeletal tissue, their differentiation capacity is quite broad and not skeleton specific. The authors of this study previously identified skeletal stem cells (SSC) that exclusively differentiate into skeletal lineage cells such as bone, cartilage and stromal cells, and through CSF antagonist/BMP2 it's been shown SSCs exhibit enhanced activity in repairing fractures in the elderly.

But different bones in different parts of the body have different mesoderm or neural crest cell origins and while most studies have focused on mesoderm derived long bones, which are formed via endochondral ossification, few have investigated the neural crest or mesoderm derived intramembranous bones of the skull. The authors therefore ask the question of whether cranial SSCs possess unique and functional attributes.

In this study the authors initially use B-actin-Cre rainbow mice to perform lineage tracing and clonal analyses within different bones, then they immunosort SSCs, demonstrate they can be successfully differentiated into bone or cartilage derivatives, evaluate their transcriptional profile, and response to BMP signaling all of which are critical for consideration in surgical skeletal repair, and may avoid some of the complications of autografts from the iliac crest.

We thank the reviewer for their thorough contextualization of our present study, which highlights the knowledge gap filled in by this work as well as its important clinical implications.

1. While it is important to optimize treatment in various areas of cranial bone repair, and the findings are interesting, they are incomplete and the functional criteria the authors want to establish are not met in an acceptable in vivo model. The key limitation is the absence of in vivo functional testing in which the SSCs are used in a critical skull bone injury model.

We thank the reviewer for this suggestion and agree with the importance of validating our findings in vivo. We have now incorporated an in vivo calvarial defect model, in order to interrogate the lineage differentiation bias of different cranial bone SSCs in the context of in vivo bone repair. In this model, a 1-mm non-critical-size defect was selected to maximize donor SSC survival and reproducible engraftment at the feasible cell dose (1×10^4 cells), to preserve periosteal and dural niche cues, and to enable mechanistic assessment of lineage allocation under controlled inflammatory and vascular conditions. These results are now described in Fig. 5 and in the manuscript on pgs. 24-27:

Cranial-SSCs exhibit region-specific lineage responses to BMP-2 *in vivo*

Lastly, we examined how the fate of cranial-SSCs changes upon BMP-2 stimulation *in vivo*. GFP^+ SSCs were harvested from GFP-transgenic mice, embedded in 1% alginate gel, and implanted into 1 mm calvarial defects (10^5 SSCs/defect) with or without added BMP-2 (1 µg/defect). After four weeks, tissues were harvested for histological analysis, with Pentachrome staining to assess matrix composition, and immunohistochemistry (IHC) for Osterix (Osx) and Sox9 to trace osteogenic and chondrogenic differentiation, respectively (**Fig. 5a**). For femur-SSCs transplanted without BMP-2, both GFP^+/Osx^+ and $GFP^+/Sox9^+$ populations were detected (**Fig. 5b-c**), indicating that both osteogenic and chondrogenic differentiation occurred among transplanted cells). Pentachrome staining showed mainly bone formation in this experimental condition (**Fig. 5b**, top left), suggesting that the presence of $Sox9^+$ cells reflects a transient endochondral component during ossification. Upon BMP-2 addition, femur-SSC implants contained predominantly GFP^+/Osx^+ cells, with a modest but nonsignificant decrease in $GFP^+/Sox9^+$ cells (**Fig. 5b,c**).

In defects implanted with parietal-SSCs, the non-BMP-2-treated condition exhibited primarily $GFP^+/Sox9^+$ cells and few GFP^+/Osx^+ cells, consistent with Pentachrome staining revealing abundant cartilage-like matrix (**Fig. 5d-e**). BMP-2 treatment of parietal-SSCs significantly increased the proportion of GFP^+/Osx^+ cells and reduced $GFP^+/Sox9^+$ cells, with Pentachrome staining confirming predominantly bone formation (**Fig. 5d-e**). Occipital-SSC implantation without BMP-2 resulted mainly in bone formation with numerous GFP^+/Osx^+ cells (**Fig. 5f-g**). Interestingly, BMP-2 treatment of occipital-SSCs induced cartilage formation, characterized by increased $GFP^+/Sox9^+$ cells and Alcian Blue-positive matrix on Pentachrome staining (**Fig. 5f-g**). Given the posterior, Hox^+ identity of occipital-SSCs (**Supplementary Fig. 9b**), this response likely reflects a positional bias that directs BMP/TGF-β signaling toward a transient Sox9-dependent, endochondral-like program *in vivo*.

Mandibular-SSC implantation produced distinct ring-like ossification structures, composed of GFP^+/Osx^+ osteoprogenitors surrounding marrow-like cavities, indicative of intramembranous ossification (**Fig. 5h**). $GFP^+/Sox9^+$ cells were rare following mandibular-SSC transplant (**Fig. 5h-i**), consistent with a direct osteogenic differentiation trajectory. Upon BMP-2 treatment, the proportion of GFP^+/Osx^+ cells increased slightly (**Fig. 5h-i**), suggesting that mandibular-SSCs, like other neural crest-derived cranial-SSCs (e.g., nasal-SSCs), respond to BMP-2 mainly by expanding the osteogenic lineage rather than initiating chondrogenesis. Nasal-SSC implantation resulted predominantly in cartilage-like tissue characterized by abundant $GFP^+/Sox9^+$ cells and minimal bone matrix (**Fig. 5j-k**). This pattern reflects a strong intrinsic chondrogenic bias under low BMP signaling conditions. In contrast, BMP-2 treatment markedly shifted the repair outcome toward bone formation, yielding extensive mineralized matrix and numerous GFP^+/Osx^+ osteoprogenitors with a corresponding reduction in $GFP^+/Sox9^+$ cells (**Fig. 5j-k**).

Quantitative comparison of the frequency of Osx^+ and $Sox9^+$ cells among GFP^+ cells from each SSC origin revealed site-specific differences in lineage commitment in the absence of BMP-2 (**Fig. 5l, m**). Occipital-SSC implants contained a significantly

higher proportion of GFP^+/Osx^+ cells compared with femur-SSC implants, whereas nasal-SSC implants showed a significantly higher proportion of $GFP^+/Sox9^+$ cells compared to femur-SSC implants (**Fig. 5l, m**), further highlighting the intrinsic differentiation capacity among cranial-SSCs. To evaluate the effect of BMP-2 across cranial-SSC types, we calculated the percentage change in GFP^+/Osx^+ and $GFP^+/Sox9^+$ fractions before and after BMP-2 treatment (**Fig. 5n, o**). Relative to femur-SSCs, nasal-SSCs exhibited a significantly greater increase in GFP^+/Osx^+ cells and a significantly and markedly greater reduction in $GFP^+/Sox9^+$ cells (**Fig. 5n, o**), indicating a robust osteogenic shift upon BMP-2 stimulation. Collectively, these results reveal that SSCs from distinct cranial regions decode BMP-2 signals in unique ways, underscoring the importance of region-specific tuning of BMP-2 delivery for optimal calvarial bone regeneration.

2. Clonal analyses were performed from P3-P33 (one month) and the nasal tissue was found to contain the highest number of clones, followed by the mandible, but the other cranial bones did not demonstrate high clonality. But what this means isn't clear. How to the authors define high versus low clonality. What's the metric? I did not see any quantification, nor any rationale or articulation of what the clonality represents, or its connection to SSCs, other than just an observation. Is there any physiological or functional difference?

We thank the reviewer for their thoughtful critique of these experiments and have made multiple changes to clarify and make these conclusions more robust. First, we have now adjusted the tamoxifen induction time from P3 to P10, in order to more selectively trace postnatal SSCs. Induction at P10 ensures sparse, clonal labeling while avoiding widespread labeling of transient embryonic progenitors that may still be present at earlier stages. At P10, cranial sutures remain active but developmental ossification has slowed, enabling reliable visualization of long-term SSC-derived clones. Second, we added a later analysis time point at P13.5 weeks, which enabled us to assess the persistence of labeled clones and distinguish long-term SSCs from short-lived progenitors. This analysis revealed that the frontal bone had a marked reduction in clonality by 13.5 weeks compared to 4 weeks, suggesting that most of the labeled cells in the frontal bone at 4 weeks represented transient progenitors rather than bona fide SSCs. These additions clarify the interpretation of "high" versus "low" clonality and strengthen the biological significance of our findings. These results are covered in the revised manuscript on pgs. 6-7:

In the frontal bone, clones were mainly found at the posterior-frontal suture and occasionally in mineralized bone areas (**Fig. 1b,** yellow circles). Similarly, in the parietal bone, clones were primarily located at the sagittal suture and sometimes in mineralized bone areas (**Fig. 1c**). In the occipital bone, numerous clones were observed within the suture mesenchyme (**Fig. 1d**). In the nasal bone, clones were mainly located in the cartilaginous area (**Fig. 1e**)**.** In the mandibular bone, clones were predominantly located in the area of the periodontal ligament (PDL) (**Fig. 1f**). Quantification of clone density in each bone revealed that clones were most

abundant in the nasal bone, followed by the occipital, mandibular, parietal, and frontal bones (**Fig. 1g**).

To further interrogate whether the observed clones in fact represented long-lasting stem cells, we examined clonality 12 weeks after tamoxifen induction, corresponding to the P13.5-week time point (**Supplemental Fig. 1a-e**). Similar to the P45 time point, the highest clonality at P13.5-weeks was observed in the nasal bone, followed by the occipital, mandibular, and parietal bones (**Supplemental Fig. 1f**). Interestingly, no clones were detected in the frontal bone at this later time point (**Supplemental Fig. 1a**). This finding was supported by quantitative comparison of clone density between P45 and P13.5-weeks (**Supplemental Fig. 2a**), suggesting that the frontal bone cells found to give rise to clones at P10 are likely progenitor cells rather than long-lasting SSCs. In contrast to this finding in the frontal bone, clone density in the parietal, occipital, mandibular, and nasal bones remained similar between P45 and P13.5-weeks (**Supplemental Fig. 2b-e**), indicating that labeled cells in these regions are in fact long-term stem cells. **Supplemental Figures 3 and 4** show the corresponding hematoxylin and eosin (H&E) staining for the P45 and P13.5-weeks time points.

3. As the authors show later, the gene expression profiles across the different bone samples are very different, and simple differences in cell adhesion could make all the difference in clonality score. Similarly, after sorting SSCs from different cranial bones, nasal derived SSCs had the highest colony forming ability, while in contrast it was low for the frontal bone. But the significance of this isn't made clear.

We thank the reviewer for this constructive comment and for the opportunity to clarify the significance of our clonality and colony-forming data. First, SSCs, regardless of the subtype heterogeneity identified in this study, are expected to adhere well under the culture conditions used; therefore, we do not anticipate that differences in cell adhesion are a major factor influencing the CFU assay results. It is possible that differential expression of adhesion-related genes among cranial SSC populations may contribute to variations in CFU formation. Although a deeper investigation of adhesion molecule expression across cranial SSCs could provide additional insight, this was not the primary focus of this study. Importantly, the enhanced colony-forming activity observed in nasal SSCs corresponds to greater clonal expansion in this region in vivo, whereas the relatively low colony-forming ability of frontal SSCs is consistent with the limited frontal bone clonality observed in vivo (Fig. 1g). These findings demonstrate that our in vitro CFU results closely mirror the in vivo clonality patterns, supporting the conclusion that differences in CFU capacity primarily reflect intrinsic, site-specific clonal skeletogenic potential of SSCs.

Pg.9-10.We first conducted an *in vitro* colony-forming assay to evaluate clonogenic potential of SSCs from each region of the cranium (**Fig. 2a**). Upon plating, nasal bone-derived SSCs exhibited the highest colony-forming ability, followed by femoral, mandibular, parietal, occipital, and frontal SSCs (**Fig. 2b**). The enhanced colony-

forming activity observed in nasal SSCs corresponds to greater clonal expansion in this bone *in vivo*, while the low colony-forming ability of frontal SSCs is consistent with limited frontal bone clonality observed *in vivo* (**Fig. 1g**). Collectively, these findings support that *in vitro* colony-forming potential does reflect the *in vivo* abundance and self-renewal capacity of SSCs within each region.

4. One of the challenges to the data is that there's no real pattern or consistency among the different bones, in terms of their clonal distribution or colony forming ability, except being highest for the nasal tissue, and the fact that nasal-SSCs do not have osteogenic capacity in the (cell culture and kidney capsule) differentiation assays performed, whereas all the different bone derived SSCs have chondrogenic ability. The osteogenic versus chondrogenic differentiation capacity may be associated with expression of bone anabolic or remodeling gene signatures which were high in the mandible and femur, but low in the nasal SSCs. But this then raises the question of why there are differences in the efficacy of grafts of mandibular bone versus femur bone in critical size bone defect repair.

We appreciate the reviewer's thoughtful comment. We agree that the superior healing capacity of mandibular versus femoral bone likely involves multiple factors beyond stem cell-intrinsic properties (e.g., differences in vascularization, mechanical loading, and marrow composition). These tissue- and site-specific environmental factors undoubtedly influence repair outcomes and interact with the intrinsic behavior of SSCs. However, the current study focuses specifically on the intrinsic diversity of SSCs and their differential response to BMP-2 stimulation, which, given the known central role of both SSCs and BMP signaling in bone healing/regeneration, are highly likely to impact site-specific healing differences. We have clarified this scope in the Discussion and noted that future studies integrating other factors, such as vascular and mechanical contexts, will be necessary to fully explain site-specific differences in regenerative capacity, on pg. 31:

Pg.31.There are likely additional factors beyond SSC heterogeneity that impact differential regeneration between bone sites. For instance, the observation that mandibular bone generally exhibits superior regenerative capacity compared with the femur likely results from multiple factors beyond SSC behavior—including region-specific vascularization, marrow composition, and mechanical loading. By thoroughly delineating the intrinsic diversity and BMP-2 responsiveness of SSCs, our study provides a framework for future investigations that integrate these other factors—such as biomechanical and vascular contexts—to fully explain and predict site-specific regenerative outcomes.

5. The data comparing the ratio of SSCs and Bone Cartilage Stromal Progenitors from P3-1 year is interesting but also somewhat confounding. At least in the skull, the first 2-3 weeks of postnatal development coincide with the highest degree of craniofacial growth so isn't it counterintuitive that SSC's ramp up after this period?

We appreciate the reviewer's thoughtful observation and the opportunity to clarify this point, as we agree that it may seem somewhat counterintuitive. First, it is important to

distinguish overall *numbers* of SSCs or BCSPs versus the BCSP/SSC *ratio.* As shown in Fig. 3a, SSC numbers are in fact high during the initial weeks of postnatal development during which, as this reviewer correctly points out, there is a high degree of craniofacial growth. Second, the BCSP/SSC ratio reflects an important and distinct aspect of stem cell dynamics which is consistent with these growth demands. As described in the manuscript, bone growth correlates with a high BCSP/SSC ratio, since BCSPs represent actively differentiating progenitors that give rise to bone, cartilage, and stromal cells. During early postnatal development (P3–P14), this ratio is elevated, reflecting robust skeletal growth driven by active SSC differentiation *into* BCSPs (such that the *relative* numbers of BCSPs are high compared to SSCs). In contrast, in mature or aged mice, the *proportion* of SSCs increases relative to BCSPs, indicating a shift toward a quiescent, self-renewing SSC pool with reduced production of committed progenitors (such as BCSPs) given limited bone growth needs. Therefore, rather than SSCs “ramping up” later in development, the observed dynamics instead reflect an accumulation of resting SSCs (as well as a relative paucity of BCSPs), as the bone transitions from a growth phase to homeostasis. We have clarified this point in the revised manuscript on pg. 32:

> Pg.32. Although SSC prevalence does appear to increase after this developmental window, this change likely reflects the accumulation of quiescent, self-renewing SSCs rather than renewed proliferative activity, and is consistent with a transition from growth to maintenance.

6. The ratio peak at 2 weeks may therefore be more relevant. I do however think it is important that the number of SSCs is higher in 1 year old mice in the mandible than the femur, when considering the source of bone for a cranial skeletal defect repair in humans or why mandible holes repair faster than femur holes. But there are many other reasons why this might be the case considering the adult functions and physiological forces and stresses that each of these bone are affected or influenced by in an adult. Nonetheless it is interesting that the SSC dynamics are different between cranial bones and long bones and this can help determine where too source the cells for the most efficacious autologous grafts.

We thank the reviewer for this insightful comment and agree that the persistently higher number of SSCs in the adult mandible likely contributes to its superior repair potential compared with long bones. As aptly noted, this difference may be further influenced by site-specific physiological factors such as mechanical loading, vascularization, and marrow environment. While our current study could not directly assess all of these factors that could impact defect healing, we believe our findings provide a cellular and molecular framework to explain these site-dependent differences and highlight the mandible as a potentially advantageous SSC source for autologous grafting.

7. The bulk RNA sequencing of SSCs isolated from each bone has the potential to reveal important differences, but too many assumptions are made about the presence of individual Wnt factors or stemness markers or their downstream genes, as well as

cytokines and ctsk. This section describing every assay and parameter and bone was really confusing. There was no coherent message connecting the dots, but more importantly the data is primarily correlative and speculative with no confirmation of function.

We agree with reviewers' concerns regarding the limited conclusions possible from these data, and have now removed the majority of the bulk RNA-seq results.

8. I have similar concerns with the experiments involving different populations of SSCs and their responses to BMP signaling. There's a lot of variation which may be possible to tie to intrinsic gene expression profiles, but again the interpretations are quite speculative and a demonstration of the mechanism underlying the differences is not provided. Overall, it's interesting that the nasal tissue has the highest number of SSCs and the greatest response to BMP2, but their differentiation is limited to chondrogenesis in the assays used. The full potency was not tested under other possible differentiation conditions.

We appreciate this concern and have now included additional experiments involving Wnt5a inhibition to investigate the mechanism underlying the low basal osteogenic activity of nasal-SSCs. Using qPCR, we identified that *Wnt5a* was significantly enriched in nasal-SSCs (Fig. 4j). We inhibited Wnt5a signaling using Box5, a potent and selective Wnt5a antagonist, and observed that Box5 treatment enhanced the osteogenic capacity of nasal SSCs under BMP-2 stimulation (Fig. 4k–m). These findings provide mechanistic insight into the reduced osteogenic potential of nasal SSCs compared with femur-derived SSCs under BMP-2 stimulation, and demonstrate that modulating this pathway can restore osteogenic differentiation. These results are delineated in the revised manuscript on pgs. 20-21:

> Pg.20-21. We next investigated the mechanisms underlying these divergent osteogenic capacities, focusing on the striking BMP-2 responsiveness we observed in nasal-SSCs. Bulk RNA-sequencing revealed that Wnt5a expression was significantly higher in nasal-SSCs compared with femur-SSCs (Supplementary Fig. 9a), which was confirmed by qPCR showing nearly 20-fold higher Wnt5a transcript levels in nasal- versus femur-SSCs (Fig. 4j). Based on previous study showing that non-canonical Wnt signaling can suppress osteogenesis in cranial stem cells36, we hypothesized that elevated Wnt5a suppresses osteogenic differentiation through enhanced non-canonical Wnt signaling. To test this, we assessed osteogenesis in femur- and nasal-SSCs treated with Box5 (100 µM), a selective Wnt5a antagonist, in the presence or absence of BMP-2 (100 ng/mL) (Fig. 4k). As expected, BMP-2 treatment alone promoted mineralization in both femur- and nasal-SSCs (Fig. 4l-m, first and second bars). However, when Box5 was administered without BMP-2, neither femur- nor nasal-SSCs demonstrated substantial enhancement in mineral deposition relative to untreated controls (Fig. 4l-m). In contrast, Box5 treatment combined with BMP-2 resulted in increased osteogenesis compared with BMP-2 alone, with a modest improvement observed in femur-derived SSCs and a significantly greater enhancement in nasal-SSCs (Fig. 4l-m). These data refine our

initial model and indicate that Wnt5a primarily acts to restrain BMP-responsive osteogenesis rather than suppress basal osteogenic differentiation. In femur-SSCs, low basal Wnt5a expression imposes minimal inhibition, resulting in only a slight enhancement when Box5 is added to BMP-2. In contrast, the high Wnt5a expression in nasal-SSCs creates a strong inhibitory threshold that can be relieved when both BMP-2 stimulation and Wnt5a blockade are present. Thus, the reduced osteogenic potential of nasal SSCs compared with femur-derived SSCs under BMP-2 stimulation (Fig. 4k) appears to be driven, at least in part, by elevated non-canonical Wnt signaling mediated by Wnt5a, which restricts their responsiveness to osteoinductive cues such as BMP-2.

9. I think it is incorrect to state for example on line 289 that “we have uncovered the genetic and functional diversity within cranial SSCs which critically influences the outcomes of bone regenerative therapies” because this was never functionally tested. Testing whether the different SSCs truly have distinct repair capacities in critical sized bone defects would seem to be an absolutely essential part of the study to validate the claims.

We thank the reviewer for this valuable comment. To address this point, we have now incorporated an *in vivo* calvarial defect model to assess SSC lineage differentiation capacity under a clinically relevant condition. This new dataset demonstrates that SSCs from distinct cranial regions exhibit site-specific differentiation preferences when implanted into bone defects. Moreover, we compared how each SSC population responds to BMP-2 stimulation *in vivo* and found clear differences in osteogenic versus chondrogenic lineage allocation across anatomical origins. Quantification of OSX^+ (osteogenic) and $SOX9^+$ (chondrogenic) donor-derived cells revealed that certain SSCs, such as nasal SSCs, respond more robustly to BMP-2 cues than others. These results, now presented in revised Figure 5, provide direct *in vivo* functional validation of the region-dependent diversity of cranial SSCs and are discussed in the revised manuscript on pgs. 24-27 (“**Cranial-SSCs exhibit region-specific lineage responses to BMP-2 *in vivo***”).

Reviewer #3 (Remarks to the Author):

The Diversity in Cranial Skeletal Stem Cells Regulates Bone Regeneration with Bone Morphogenetic Proteins

In this study, Takematsu et al. (2024) report the diversity in cranial skeletal stem cells (SSCs) regulates bone regeneration with bone morphogenetic proteins. They evaluated the clonality of osteogenic lineage cells in the cranial bones by using Actb-CreERT;Rainbow mouse. They next isolate SSCs from each bone (femur, frontal, parietal, occipital, nasal, and mandible) and compared the clonal capacity, the differentiation capacity, and the gene expression property. They demonstrated that higher osteogenic property in the SSCs isolated from femur, occipital and mandible, while cells from nasal cells showed high chondrogenic property. They also evaluated the percentage of SSCs, osteogenic cell, chondrogenic cells, and bone cartilage stromal progenitors (BCSPs) in the isolated cells from each bone. From DEG analysis of RNA sequence, authors presented marker genes specific for each bone. Finally, they tested the effects of bone morphogenetic protein (BMP)-2 on induction osteogenic capacity of isolated cells.
The viewpoints of this study are valuable, and they identified different properties of osteogenic lineage cells in each cranial bone. However, results are still descriptive and lacking molecular functional analyses explaining each cellar properties and gene expression. The study could be very interesting but it is too premature at this point. The manuscript is too unfriendly to the readers.

We thank the reviewer for their careful reading of our study and appreciate their recognition of the value of this work. We sincerely appreciate the critiques regarding the limitations of our original study; we believe the manuscript is now much strengthened by our revisions, which are outlined below in response to specific comments.

I also describe some points which require to improve this study.

1. From which stage of mice did the authors isolated cells for in vitro study? The property of the cells must be different between stages. I could not find it but even if the authors describe it in the text, they should mention it in the result section.

Cells for in vitro experiments were isolated from P3 mouse pups. We regret omitting this information from the original manuscript and now include it in the revised manuscript on pg. 9:

> Pg.9. In order to evaluate the intrinsic functional ability of SSCs residing in each cranial region, we dissected the cranial bones from P3 mice pups and prospectively isolated SSCs from these bones using fluorescence activated cell sorting (FACS) with surface markers established previously[4] (FACS gating strategy demonstrated in **Supplemental Fig. 5**). We also isolated P3 femoral SSCs in the same manner for comparison.

While we agree with the reviewer's point that these cells' properties could differ between developmental stages, we feel that a full developmental time course of these *in vitro* studies would be outside of the scope of the present study. However, we do believe the ensuing *in vivo* analyses—which compare SSCs at multiple postnatal time points representing distinct stages of craniofacial development (e.g., Fig. 3)—provide valuable insight into regional SSC dynamics over the course of craniofacial development.

2. It is difficult to understanding the tissue structure from Figure 1b-j. Haematoxylin-Eosin staining should be included with fluorescent images. Because it is very important to understand original tissues to consider the properties of isolated cells.
We appreciate this suggestion, as this addition is important for contextualizing our findings and particularly so for readers who may be less familiar with craniofacial skeletal architecture. H and E staining images for the corresponding confocal images are now shown in Supplemental Figures 3 and 4.

3. It is difficult to understand the meaning of the figures. Appropriate labelling is required. It is not reader friendly.

We have made every effort to revise all figure legends for maximum clarity.

4. Nasal bone has large cartilaginous region. Authors may also show the cartilaginous region of nasal bone in Figure 1b. They tested the chondrogenic properties of SSCs, but it is considered that isolated cells from the nasal bone were chondro-progenitors rather than SSCs.

We have incorporated new experiments which provide added insight into a potential molecular basis for the distinct properties observed in nasal-SSCs. After careful examination of gene expression in nasal-SSCs, we found that Wnt5a was highly expressed in nasal-SSCs compared with other SSC populations (Fig. 4j). Blocking Wnt5a signaling with Box5 treatment restored the osteogenic capacity of nasal SSCs (Fig. 4k, m). These results indicate that nasal SSCs possess an intrinsic ability to differentiate into bone, but this potential is suppressed under basal conditions by non canonical Wnt regulatory pathway. This mechanism and its functional consequences are presented in detail in the Results section on pgs. 20-21:

> Pg.20-21. We next investigated the mechanisms underlying these divergent osteogenic capacities, focusing on the striking BMP-2 responsiveness we observed in nasal-SSCs. Bulk RNA-sequencing revealed that Wnt5a expression was significantly higher in nasal-SSCs compared with femur-SSCs (Supplementary Fig. 9a), which was confirmed by qPCR showing nearly 20-fold higher Wnt5a transcript levels in nasal- versus femur-SSCs (Fig. 4j). Based on previous study showing that non-canonical Wnt signaling can suppress osteogenesis in cranial stem cells36, we hypothesized that elevated Wnt5a suppresses osteogenic differentiation through enhanced non-canonical Wnt signaling. To test this, we assessed osteogenesis in femur- and nasal-SSCs treated with Box5 (100 μM), a selective Wnt5a antagonist, in the presence or absence of BMP-2 (100 ng/mL) (Fig. 4k). As expected, BMP-2

treatment alone promoted mineralization in both femur- and nasal-SSCs (Fig. 4l-m, first and second bars). However, when Box5 was administered without BMP-2, neither femur- nor nasal-SSCs demonstrated substantial enhancement in mineral deposition relative to untreated controls (Fig. 4l-m). In contrast, Box5 treatment combined with BMP-2 resulted in increased osteogenesis compared with BMP-2 alone, with a modest improvement observed in femur-derived SSCs and a significantly greater enhancement in nasal-SSCs (Fig. 4l-m). These data refine our initial model and indicate that Wnt5a primarily acts to restrain BMP-responsive osteogenesis rather than suppress basal osteogenic differentiation. In femur-SSCs, low basal Wnt5a expression imposes minimal inhibition, resulting in only a slight enhancement when Box5 is added to BMP-2. In contrast, the high Wnt5a expression in nasal-SSCs creates a strong inhibitory threshold that can be relieved when both BMP-2 stimulation and Wnt5a blockade are present. Thus, the reduced osteogenic potential of nasal SSCs compared with femur-derived SSCs under BMP-2 stimulation (Fig. 4k) appears to be driven, at least in part, by elevated non-canonical Wnt signaling mediated by Wnt5a, which restricts their responsiveness to osteoinductive cues such as BMP-2.

5. Authors should describe material and method or experimental strategies more kindly, for example, age and gender of used mice, tissue isolation procedures, and detail information of the antibodies.

We now provide all of these details in the revised Methods section.

6. Authors define the SSCs properties from each bone, but they also should show which part of the tissue they originally isolated cells from.

Detail on the tissue isolation method is now included in the Methods section on pgs. 34-35:

FACS isolation and analysis of mouse SSCs

The distal femur; frontal, parietal, occipital and nasal bones; and mandible were dissected from postnatal day 3 (P3) mice of both sexes. For the frontal bone, the posterior frontal suture was included; for the parietal bone, the sagittal suture was included; and for the occipital bone, the lambdoid suture was included. Dissections of the nasal bone encompassed the maxilloturbinate and nasoturbinate regions. For the mandible, due to the technical difficulty of removing developing molar teeth and to avoid the potential loss of stem cells associated with the periodontal ligament, all tissue compartments—including the molar teeth—were collected.

Response to Reviewers

Table of Contents

Reviewer comments	Page Number
Reviewer 2	
Comment 1-2	Page 2-3
Comment 3-4	Page 3-4
Comment 5-6	Page 4
Comment 7-8	Page 5-6
Comment 9	Page 6
Comment 10-12	Page 7-8
Reviewer 3	
Comment 1-2	Page 8-9
Comment 3-4	Page 9-10
Comment 5-6	Page 10-11
Comment 7-8	Page 11-12
Reviewer 1 (Provided by Reviewer 2)	
Comment1	Page 12-13

Reviewer #2 (Remarks to the Author)

The authors should be commended for their efforts to revise their manuscript in response to the reviewers' concerns. I especially appreciate the addition of the critical size calvarial bone defect transplantation repair assay. This was not a trivial experiment but the new data does provide important in vivo support for the authors' interpretations and conclusions. Removing some of the transcriptomic data together with elimination of the speculation in the previous submission has strengthened and tightened the study.

→ We thank the reviewer for their positive and encouraging feedback and for recognizing the improvements made in the revised manuscript.

My remaining questions are comparatively minor but clarification can further strengthen the study.

1. For the benefit of the readers, especially non-experts, it would be helpful to indicate the plane of section in the clonal and other skull analyses.

→ Thank you very much for your suggestion. Revised manuscript now indicates the plane of section.

Pg9 Ln160-161: Note: b-e is coronal and f is sagittal section
Pg28 Ln513-514: All images are sectioned in the coronal plane.

2. The clonality score is still somewhat confusing because, although clones were noted, the methodology used doesn't convey a complete evaluation of the total number of clones. How does one ensure clones were not missed in the sea of different colors which to my eye can make it difficult to identify all the clones in the first place.

→ We thank the reviewer for this important comment and for highlighting the need for clarification regarding clonality analysis and lineage tracing strategy.

For clarification of clonality scoring and clone detection, we agree that identification of clones in multicolor reporter systems can be challenging due to color complexity. In our study, clonality was not assessed solely by visual color distinction, but by integrating spatial continuity, cellular morphology, and color similarity across contiguous cells. Specifically, clones were defined as groups of adjacent cells sharing similar spectral signatures and forming spatially coherent clusters, rather than isolated color-matched cells. To minimize the risk of missing clones, we used high-resolution imaging to capture entire sections, and analyses were performed systematically across the full section rather than selected fields. In cases of ambiguous color overlap, spatial criteria (cell adjacency and continuity) were

prioritized over color alone. All clonality assessments were conducted in a blinded manner to reduce bias. We acknowledge that multicolor reporter systems such as Rainbow may underestimate the total number of clones due to color convergence. Therefore, the clonality score reported here should be interpreted as a conservative estimate of clonal expansion rather than an absolute quantification. This limitation has now been clarified in the revised manuscript (Method sections).

Pg 39 Line 754-759: Clone identification and counting were performed in a blinded manner. For each section, the number of distinct clones per area (mm^2) were recorded. Due to the stochastic nature of reporter recombination and the limited number of distinguishable colors, some clones with similar color profiles may not have been resolved; therefore, clone counts likely represent an underestimate of true clonal frequency.

3. Also, can the authors better justify β actin-CreERT/Rainbow instead of maybe Sox10-CreERT as an indelible label of SSCs?

→ We appreciate the reviewer's suggestion regarding lineage specificity. We used β-actin-CreERT/Rainbow to achieve broad, unbiased labeling of all cell populations, allowing assessment of clonal behavior without restricting analysis to a predefined lineage. This is particularly important as the cellular origins of skeletal stem cells (SSCs) remain under investigation. In contrast, Sox10-CreERT labels a more restricted neural crest–derived population and may overlook contributions from other progenitors. Our aim was to capture global clonal dynamics within the tissue rather than lineage-restricted tracing. We have now clarified this rationale in the revised manuscript.

Pg 6 Ln 115-118: The β-actin-CreERT/Rainbow system enables assessment of clonal dynamics without restricting analysis to a predefined lineage. Thus, labeled cells may include SSCs and/or other progenitor populations. This approach allows comprehensive evaluation of cellular contributions within the tissue, which would not be achievable using lineage-specific drivers.

4. Line 60 reports the Actb-CreERT (βactin-CreERT/Rainbow) mice, but the methods refer to ActinCreER-R26VT2/GK3. The nomenclature for the mice used should be consistent throughout the manuscript.

→ Apologies for this inconsistency. We fixed the nomenclature under method section.

Pg 35 Ln672: For clonality analysis, we utilized ***β actin-CreERT/Rainbow*** transgenic reporter mice. These mice were generated by crossing the ActinCreER transgenic mouse with the R26VT2/GK3 transgenic mouse, to enable Cre-dependent cell labeling with a 4-color reporter construct at the ROSA locus. In this construct, tamoxifen delivery activates Cre recombinase that induces random and specific recombination, causing Cre-positive cells to express 1 of 10 possible color combinations of 4 fluorescent proteins at the ROSA locus: GFP (green fluorescent protein), CFP (cyan fluorescent protein), RFP (red fluorescent

protein), or OFP (orange fluorescent protein). Actin is ubiquitously expressed; thus, tamoxifen induction of ***β actin-CreERT/Rainbow*** mice allows for an unbiased in vivo survey of clonal activity by all potential cell types, rather than specific lineage-restricted promoters.

5. Why do the authors think there were no clones in the P13.5W neural crest cell derived frontal bone?

→ In the image, some labeled cells are visible; however, these are primarily located within the marrow space or represent green-colored cells. As GFP represents the baseline (non-recombined) color, green-only cells were excluded from clonal analysis. Therefore, we concluded that no clones were present at the P13.5-week time point. In addition, cells labeled at P10 showed clear clonal expansion in the frontal bone at earlier time points (e.g., P45), but not at P13.5 weeks. This suggests that the labeled cells contribute transiently and do not persist long-term, consistent with progenitor-like behavior rather than long-lasting SSCs. This has now been clarified in the revised manuscript.

Pg7 Ln144-145: The transient nature of clonal contribution suggests that these cells lack long-term self-renewal, a defining feature of skeletal stem cells.

6. If they are progenitor cells instead of SSCs, is this a limitation with the βactin-CreERT/Rainbow approach or are those original neural crest cells replaced by some other non βactin-CreERT cell type?

→ We thank the reviewer for this important comment. The β-actin-CreERT/Rainbow system labels cells in a broad and unbiased manner at the time of induction, enabling lineage tracing of all recombined cells and their progeny. Therefore, the observed clones reflect the behavior of labeled progenitor populations present at induction, regardless of whether they meet strict definitions of SSCs. We acknowledge that this approach does not distinguish SSCs from other progenitor cell types, which represents a limitation of the system. However, it allows comprehensive assessment of clonal dynamics without restricting analysis to a predefined lineage. We cannot exclude the possibility that some cell populations were not labeled due to incomplete recombination efficiency. Nonetheless, β-actin/CreERT is widely used for ubiquitous labeling, and thus unlabeled contributions are expected to be minimal. We have now clarified this limitation in the revised manuscript.

Pg6 Ln115-118: The β-actin-CreERT/Rainbow system enables assessment of clonal dynamics without restricting analysis to a predefined lineage. Thus, labeled cells may include SSCs and/or other progenitor populations. This approach allows comprehensive evaluation of cellular contributions within the tissue, which would not be achievable using lineage-specific drivers.

7. The small number of SSCs in the frontal bone and their low colony forming assay ability was surprising – any explanation – are they NCC derived or rapidly replaced. Is it the bone of maximum growth in the skull during the post -natal period analysed?

→ We thank the reviewer for this insightful observation. The low frequency and clonogenic potential of frontal SSCs do not necessarily reflect replacement of neural crest–derived cells, as NCC-derived skeletal cells are known to persist into adulthood. Rather, our findings suggest that the frontal bone may rely more on short-lived progenitor populations with limited long-term self-renewal capacity. This is consistent with a progenitor-dominant model of tissue maintenance, potentially influenced by regional differences in developmental origin, growth dynamics, or niche signaling.

Pg 29/30 Ln 555-558: As a neural crest–derived bone, the frontal bone may rely more on transient progenitor populations than on a long-lived SSCs. In addition, the frontal bone undergoes active growth and remodeling during the postnatal period analyzed, which may further favor progenitor turnover over maintenance of a stable SSC population.

8. The data is a bit confusing because at P21, the nasal bones have comparatively more SSCs than other calvarial bone regions. And yet, the that nasal bone doesn't have osteogenic capacity but primarily forms cartilage under renal capsule assay? Can the authors account for this or does it come down to Wnt5-Bmp2 competing expression?

→ We thank the reviewer for this insightful comment. For the renal capsule assay, SSCs were isolated from P3 mice rather than P21, as the frequency of SSCs at P21 and later stages is substantially lower, making it difficult to obtain sufficient cell numbers for transplantation (as shown in Fig. 3a, ~200 SSCs per million cells at P21). We have now clarified this point in the revised manuscript.

Pg 10 Ln 191-193: We further demonstrated the intrinsic functional activity of cranial-SSCs *in vivo* using renal capsule transplantation. SSCs ***isolated from P3 mice*** from each region (100K cells/graft) were transplanted into the kidney capsule.

Pg 14 Ln 271-273: Representative Pentachrome staining of recipient kidneys 4 weeks following renal transplant of SSCs (1×10^5 SSCs ***isolated from P3 mice*** per implantation) from the indicated bones.

Pg 38 Ln 736-738: Then, a 2-mm incision was created in the renal capsule using a needle bevel, and 10×10^4 FACS-purified SSCs ***isolated from P3 mice were*** resuspended in 3 μl of Matrigel and transplanted beneath the capsule.

→ We also note that Wnt5 signaling alone does not appear to inhibit osteogenic differentiation, as addition of Box5 was not sufficient to restore osteogenesis *in vitro*; osteogenesis was enhanced only when Box5 was combined with BMP-2. These findings

suggest that the observed chondrogenic outcome is likely context-dependent rather than solely driven by Wnt5 signaling. This further indicates that additional regulatory factors are required to control the fate of nasal SSCs under basal conditions. Furthermore, cell fate decisions may be established at earlier developmental stages (e.g., neonatal time points), which were not addressed in this study. Although these possibilities are beyond the scope of the current work, we have included this discussion in the revised manuscript.

Pg 30 Ln 575-577: These findings suggest that additional regulatory factors are required to control the fate of nasal SSCs under basal conditions. Furthermore, cell fate decisions may be established at earlier developmental stages (e.g., neonatal time points), warranting further investigation.

9. Why do immunophenotypically defined SSCs from different cranial regions display distinct functional activities – are there any known differences in the physiology of occipital versus parietal versus frontal versus nasal bone (apart from progenitor cell origin). The authors allude to a difference in the mandible which may have a physiologcal-mechanical underpinning, but what about for the calvarial bones? Or is it simply technical and and experimental limitation.

→ We thank the reviewer for this important question. We believe the observed functional differences among immunophenotypically defined SSCs from distinct cranial regions are driven by region-specific biological factors rather than solely technical variation. In addition to differences in developmental origin, these regions are associated with distinct microenvironments that can influence SSC behavior. One possible explanation is that neural crest–derived SSCs may be regionally primed at earlier developmental stages, resulting in lineage-biased differentiation observed postnatally. This could account for the preferential chondrogenic or osteogenic differentiation observed in nasal and mandibular SSCs, respectively. Notably, the occipital region is associated with substantial muscle attachment and mechanical loading, which may influence the local niche through mechanotransduction and muscle-derived signaling factors, potentially promoting osteogenic differentiation and contributing to the observed lineage bias of occipital SSCs. In contrast, parietal regions, which experience less direct mechanical loading, may maintain a more permissive environment that supports broader differentiation potential. At the same time, we acknowledge that technical and experimental factors, including cell yield and isolation efficiency, may contribute to some variability. However, the consistent trends observed across multiple assays support a biological basis for these differences. We have expanded the Discussion to reflect these points.

Pg 31 Ln 580-584: Notably, regional differences in SSC behavior may also reflect distinct microenvironmental influences. For example, the occipital region is associated with greater muscle attachment and mechanical loading, which may promote osteogenic differentiation through niche-dependent cues. In contrast, parietal regions may provide a more permissive environment that supports broader lineage potential.

10. The BCSP/SSC ratio is an intriguing measure for active growth but how do the authors correlate that with the relative paucity of SSCs in the nasal bones compared to other cranial bones and what can the authors say about the distinct growth patterns of the nasal bone relative to other bones that could account for this.

→ We thank the reviewer for this insightful comment. The nasal bone exhibits distinct growth characteristics compared to other cranial bones, including a greater association with cartilage structures and a bias toward chondrogenic differentiation. These features may contribute to both the lower SSC frequency and the distinct functional behavior observed in this region. We further speculate that an increased demand for lineage-committed cells during earlier developmental stages may reduce the relative SSC pool in the nasal bone compared to other cranial regions. We have now incorporated this interpretation into the Discussion section.

Pg 32/33 Ln 622-624: We further speculate that an increased demand for lineage-committed cells during earlier developmental stages may reduce the relative SSC pool in the nasal bone compared to other cranial regions (**Figure 3c**).

11. It's interesting that nasal SSCs exhibited the strongest response to BMP-2. Maybe the nasal SSCs are a bit of an enigma because, they produce the highest frequency of clones, yet in the critical size calvarial bone defect assay the nasal SSCs predominantly formed cartilage unless treated with BMP2.

→ We thank the reviewer for this insightful observation. We agree that nasal SSCs exhibit a distinct functional profile, characterized by a high clonal frequency yet a predominantly chondrogenic outcome in the calvarial defect assay under basal conditions. We interpret this as evidence that nasal SSCs are not inherently limited in osteogenic potential, but rather are biased toward chondrogenic differentiation in the absence of appropriate cues. The strong response to BMP2 suggests that these cells retain osteogenic competence but require additional signaling input to activate this program. Thus, the observed discrepancy likely reflects context-dependent differentiation rather than intrinsic lineage restriction. We have clarified this point in the Discussion of the revised manuscript.

Pg 31 Ln 594-598: Notably, despite exhibiting high clonal frequency, nasal SSCs predominantly formed cartilage under basal conditions, indicating that proliferative capacity and lineage outcome are independently regulated. This suggests that nasal SSCs are highly proliferative yet exhibit a context-dependent lineage bias, requiring additional cues such as BMP-2 to fully activate osteogenic differentiation.

12. If cranial-SSCs decode BMP-2 cues in a region-dependent manner, what does the field now do with this information when it comes to calvarial or other cranioskeletal

repair? Is like for like repair the recommendation wherever possible? Concluding the manuscript with a recommendation based on the authors data would make the manuscript more complete.

→ We thank the reviewer for this important suggestion. Our findings indicate that cranial SSCs exhibit region-specific responses to BMP-2, suggesting that a "one-size-fits-all" approach to craniofacial repair may not be optimal. Instead, our data support the concept that repair strategies should consider the regional identity of the target tissue. In particular, tailoring BMP-2 dosage and delivery to the local cellular context, and where feasible, employing region-matched or lineage-appropriate cell sources, may improve regenerative outcomes. We have now incorporated this perspective into the concluding section of the Discussion.

Pg 33 Ln633-637: Importantly, these results suggest that craniofacial repair strategies should account for region-specific SSC behavior, as a uniform BMP-2–based approach may not be optimal across different cranial sites. Where feasible, tailoring morphogen delivery and considering region-matched cellular sources may improve regenerative outcomes.

Reviewer #3 (Remarks to the Author)

The manuscript entitled "The Diversity in Cranial Skeletal Stem Cells Regulates Bone Regeneration with Bone Morphogenetic Proteins" has been revised. The results in the manuscript indicate the importance to optimize of BMP-2–based bone regeneraton therapies. The first part that analyzed the SSC status during mouse postnatal became clearer to suggest that the ratio of SSC number and BCSP number could be explained by the requirement of Bone Cartilage Stromal Progenitors.

→ We thank the reviewer for this positive comment and are pleased that the revised manuscript more clearly conveys the relationship between SSC and BCSP populations.

1. In the introduction, when the proper name of BCSC is not written when it first appears.

→ We apologize for not spelling out BCSP when it first appeared in the manuscript. This has now been corrected in the Introduction.

Pg 4 Ln74-75: (Bone Cartilage Stromal Progenitors /Skeletal Stem Cell ratio)

2. It may not be the main interference of the manuscript but in Fig.3, the authors write that the mandible generally heals more rapidly than the femur. They attribute the finding to the higher number of mandibular SSC. At the beginning, the authors find the main SSC localization in the mandible is the periodontal ligament. Does it mean the healing is better when mandibular body was broken compared to when that ramus of the mandible is broken? Since dental mesenchyme could be different from

that to form mandibular bone since the periodontal ligament is mainly derived from the dental follicle that is dental mesenchyme. The dental follicle forms alveolar bone but not mandibular bone during development. The explanation seems to be required.

→ We thank the reviewer for this insightful comment. We agree that SSC localization within the mandible is region-specific, with enrichment observed in the periodontal ligament (PDL). Our statement that the mandible exhibits enhanced healing compared to the femur refers to overall regenerative capacity and should not be interpreted as uniform across all mandibular regions. We cannot exclude the possibility that healing capacity differs between anatomical subregions, such as the alveolar region versus the mandibular body or ramus. We also acknowledge that dental mesenchyme–derived PDL cells are developmentally distinct from those contributing to mandibular bone formation. However, these cells may still contribute to local regenerative processes by serving as a progenitor pool under injury conditions. We have clarified this point in the revised manuscript to avoid overgeneralization.

Pg 15 Ln 301-305: Importantly, SSC enrichment within the mandible is primarily localized to the periodontal ligament–associated alveolar bone (**Fig.1f**), which arises from dental follicle–derived mesenchyme. Therefore, these findings likely reflect progenitor dynamics within tooth-associated niches and may not uniformly represent all mandibular regions, such as the body or ramus.

3. The authors did not analyze the frontal SSC in Fig. 2 since they could not obtain a good number of cells, however, Fig.3 shows that the rate of the frontal SSC is more or less the same.

→ We thank the reviewer for this comment. Although SSC frequencies in Fig. 3a appear similar at P3, it is important to note that the y-axis is presented on a logarithmic scale, which can visually compress differences between groups. In absolute terms, the average SSC count in the frontal bone (~18,589 per million cells) is substantially lower than that in the nasal bone (~30,158 per million cells). In addition, the frontal bone is relatively small, limiting the total number of cells that can be obtained per litter. As a result, we were unable to isolate sufficient numbers of frontal SSCs to perform both osteogenic and chondrogenic assays reliably, and therefore excluded this group from further functional analysis. We have clarified this point in the revised manuscript.

Pg 19 Ln 354: Data are shown on a logarithmic scale.

4. Were Fig.2g and h results obtained from SSC or after differentiation induction?

→ We thank the reviewer for this question. Figures 2g and 2h were obtained from freshly isolated SSCs, without differentiation induction. We have clarified this in the figure legend.

Pg 14 Ln280-282: **(g-h)** Quantification of relative expression levels of **(g)** chondrogenic genes *Acan, Col2, Col9, and Sox9*; and **(h)** osteogenic genes *Alpl, Col1, Sp7, Bglap, Spp1, and Runx2;* from qPCR analysis of ***freshly isolated*** SSCs from indicated bones.

5. In Fig. 4, the authors demonstrate that Wnt5a is highly expressed in the nasal SSC, that is the reason of different response to BMP2 from the femur SSC. However, BMP2 addition can induce calcification in the nasal SSC although addition of Box5 enhanced the effect. Are there any possibilities that BMP2 treatment affect Wnt signaling?

→ We thank the reviewer for this insightful question. It is possible that BMP2 influences Wnt5a signaling. Previous studies (e.g., Nemoto et al.) have demonstrated crosstalk between BMP2 and Wnt5a in osteoblasts, suggesting that some interactions may occur in SSCs. However, as we did not directly assess Wnt pathway activity following BMP2 stimulation in this study, the precise interaction between BMP2 and Wnt5a remains to be determined. We have clarified this point in the revised Discussion.

Pg 21/22 Ln416-420: BMP-2 may interact with Wnt5a signaling; this interaction may contribute to the enhanced effect of Wnt5a inhibition by Box5 in the presence of BMP-2. However, as we did not directly assess Wnt pathway activity following BMP-2 stimulation in this study, the precise interaction between BMP-2 and Wnt5a remains to be determined.

6. Fig. 5 is a chaos. It is hard to recognize the contribution of each SSC in the images. For instance, in Fig. 5J Nasal SSC appears to form the cartilage like structure without BMP2, however, with BMP2 newly formed bone osteocytes seem to be negative for GFP. Didn't nasal SSC contribute to bone formation? In this case, since there are many players, it is hard to get the simple answer. However, the authors carried out a lot of experiments, the readers would like to have as simple answer as possible.

→ We thank the reviewer for this helpful comment and apologize for the lack of clarity in the original images. In the revised manuscript, we have added more arrows to highlight GFP^+/Osx^+ and $GFP^+/Sox9^+$ cells to better indicate lineage contributions. Regarding Fig. 5J, nasal SSCs do contribute to bone formation upon BMP-2 treatment. GFP^+/Osx^+ cells indicate that transplanted SSCs differentiate into osteoblasts. Although we did not assess terminal osteocyte markers (e.g., Sclerostin), GFP^+ cells are also observed within the bone matrix, suggesting that nasal SSC–derived cells contribute to newly formed bone. Additionally, to provide a simple answer for reader, we have added conclusion sentences to better convey our message.

Pg26 Ln 490-491: Nasal SSCs further contributed to bone formation upon BMP-2 treatment, as indicated by GFP^+ cells within newly formed bone (**Fig. 5j**).

Pg26 Ln502-505: Overall, BMP-2 induces region-specific lineage responses in cranial SSCs: nasal and parietal SSCs shift from chondrogenic to osteogenic differentiation, mandibular SSCs enhance osteogenesis without initiating cartilage formation, and occipital SSCs exhibit a distinct response with slightly increased chondrogenesis.

7. One more concern is that the authors are looking at the results. After bone defect formation, the healing process starts at least by day 7 and it might be appropriate to observe earlier stage.

→ We thank the reviewer for this important suggestion. We agree that examining earlier time points would provide additional insight into the initial repair process and lineage decisions after defect formation. In this study, we focused on the 4-week endpoint to assess overall regenerative outcome, while recognizing that earlier stages such as day 7 may be informative and warrant future investigation. This limitation is now noted in the result section.

Pg24 Ln455-457: We focused on the 4-week endpoint to assess overall regenerative outcome, while recognizing that earlier stages such as day 7 may be informative and warrant future investigation.

8. In addition, all cell types were implanted to the parietal defect. It is probably the best place to carry out stable experiments, however, It is possible that environmental factors implanted into the parietal bone might have some effects on the results.

→ We thank the reviewer for this important point. We agree that the parietal defect environment may influence the behavior of transplanted SSCs. We selected the parietal bone as a standardized and well-established site to enable consistent comparison across SSC populations. While local niche factors may contribute to the observed outcomes, the consistent, region-specific differentiation patterns observed across multiple SSC batches suggest that intrinsic properties of the cells play a major role. We have now acknowledged this in the result section.

Pg 24 Ln 457-460: While local niche factors may contribute to the regenerative outcome, parietal bone was selected as the defect site because it is a standardized and well-established location for consistent comparison across SSC populations.

Is Fig6 required? There must be a better way to describe the authors' observation.

→ We thank the reviewer for this suggestion. We agree that clarity of Fig. 6 can be improved. We have revised the schematic to clearly illustrate baseline lineage bias and BMP-2–induced shifts across SSC populations.

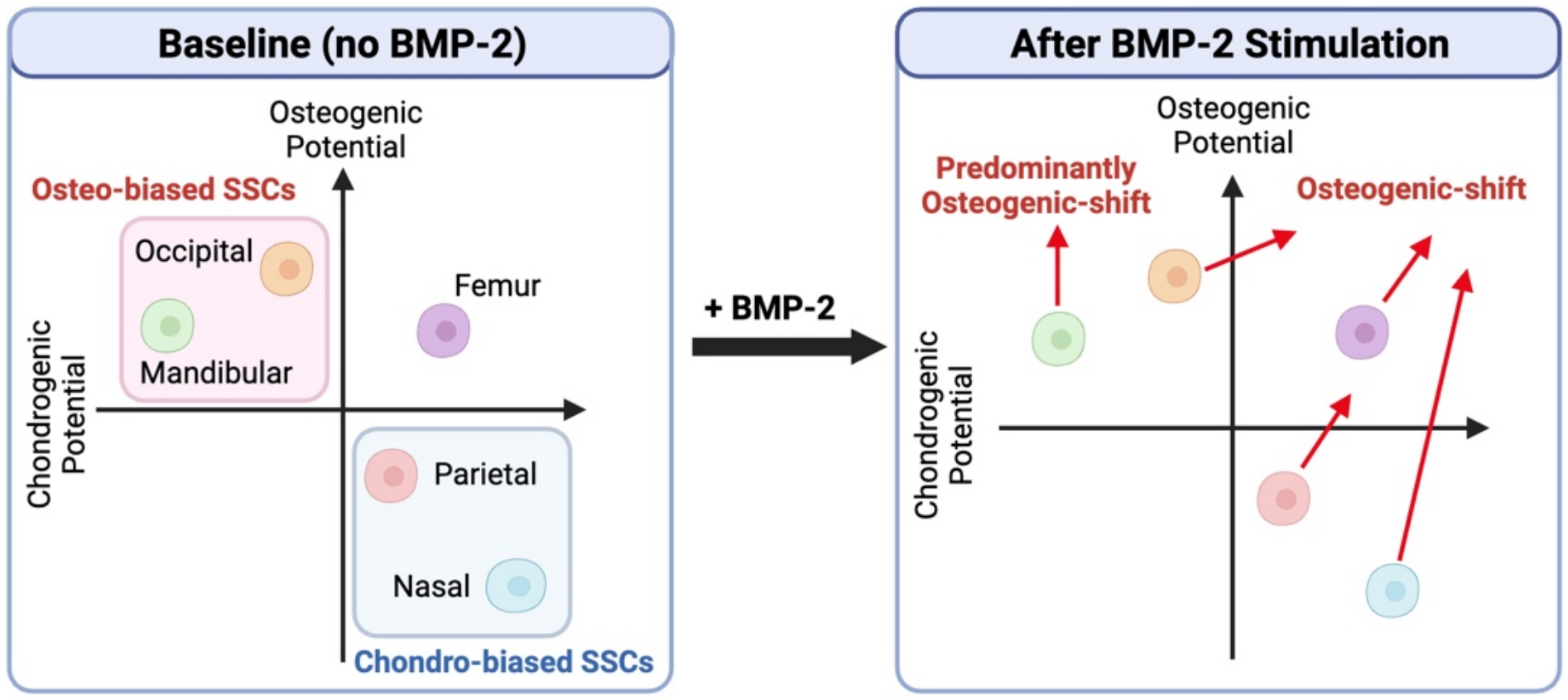

In addition to the points listed below, we asked both referees to comment on your response to reviewer #1 as we were unable to obtain further comment from this referee. Both reviewers agreed that the response to this referee was largely satisfactory. However, reviewer #2 did have some additional comments regarding your response to referee #1 point #4.

1. Reviewer #2 questioned whether there is really a standardized time point in the field for clonal analysis. The reviewer pointed to Clayton et al 2007 who analysed clonality in the skin beginning 2 days post tamoxifen injection and continuing for up to 12 months and Rinkevich et al 2011 & Rinkevich et al 2015 who analyzed the mice at 1, 2, 4 and 7 months and used 8 clustered cells to define a clone. In addition, the reviewer indicated that some additional information/justification is needed as to why 5 cells of the same color in a cluster is used to define a clonal population.

→ We thank the reviewer for this important comment. We agree that there is no universally standardized time point for clonal analysis, and that the timing and criteria vary depending on the tissue context and experimental design. Previous studies, such as Clayton et al. (2007) and Rinkevich et al. (2011, 2015), have used different induction-to-analysis intervals and clone size thresholds tailored to their respective systems. In our study, we selected our time points to capture stable clonal expansion rather than early transient labeling, focusing on later stages to assess sustained progenitor contribution. Regarding clone definition, we used a threshold of ≥5 contiguous cells of the same color to define a clone. This criterion was chosen to minimize false-positive identification due to stochastic labeling overlap or imaging noise, and thus represents a conservative estimate of clonal expansion. We note that other studies have used different thresholds (e.g., ≥8 cells), reflecting differences in tissue architecture and labeling systems. We have now clarified both the rationale for time point selection and clone definition in the revised Methods section.

Pg 39 Ln 750-754: Clones were defined as clusters of ≥5 contiguous cells sharing similar spectral signatures, a threshold selected to minimize false-positive identification due to stochastic color overlap and to provide a conservative estimate of clonal expansion. There is no universal standard for clone size thresholds or analysis time points, and these parameters vary depending on tissue context and labeling system; thus, our criteria were selected to provide a conservative and robust estimate of clonal expansion

Table of Contents

Reviewer comments	Page Number
Reviewer 2	
Comment 1	Page 2
Reviewer 3	
Comment 1-5	Page 3
Comment 6-7	Page 4
Comment 8	Page 5
Comment 9-10	Page 6

Reviewer #2 (Remarks to the Author):

The authors have satisfactorily addressed my primary concerns and clarified a number of issues which has improved their paper.

I am however still hung up on the description of the Cre line, because some of the authors of this paper have used the same clonal labeling strategy in previous papers, most notably Rinkevitch et al., 2015 where the line was first denoted as ActinCreER (and a gift from Liqun Lo) to generate ActinCreER; R26VT2/GK3 mice, and again in Zhao et al 2023 where the mice were denoted as ActinCreER/R26VT2/GK3 Rainbow reporter mice.

If the authors now call this line B-ActinCreERT then it becomes near impossible and confusing to trace the origins of original Cre line used in their study and this is important for accuracy and context. The original tamoxifen inducible B-ActinCre (B-ActinCreERT) line was generated by Guo, Yang and Lobe (https://doi.org/10.1002/gene.10021)

In the interests of the field and to be consistent with prior literature (especially papers published by some of the same authors) please use the original nomenclature throughout the manuscript so that the field clearly understands what Cre line was used and what was the original source of the Cre line used (with citations). This is critical for rigor and reproducibility.

→ We thank the reviewer for this thoughtful comment and agree that consistent nomenclature is important for rigor, reproducibility, and continuity with prior literature. To address this concern and avoid confusion regarding the origin of the Cre line used in this study, we have revised the manuscript to consistently use the nomenclature "$Actin^{CreER}$;$R26^{VT2/GK3}$" throughout the manuscript, consistent with prior publications using the same clonal labeling strategy (e.g., Rinkevitch et al., 2014; Zhao et al., 2023). We appreciate the reviewer's suggestion, which has improved the clarity and traceability of the genetic model used in this study.

Pg4Ln69, Pg6 Ln110,112,114, Pg9Ln154,158, Pg30Ln554, Pg37Ln686, 687, 693, Pg40Ln756; Nomenclature changed to $Actin^{CreER}$;$R26^{VT2/GK3}$

Reviewer #3 (Remarks to the Author):

The manuscript has been revised and now easier to understand the content. However, it still has the points to be proved.

1. Some Figures do not show the temporal sequence. Fig.1 f and g should be swapped. In Fig. 5, l and m should be located at least before b.
→ We thank the reviewer for pointing out the issue regarding temporal sequence in the figures. In response, we have switched the positions of Fig. 1f and g to improve the chronological flow of the data presentation. Regarding Fig. 5, we appreciate the reviewer's suggestion. However, we intentionally positioned panels l and m after panels b–k because these panels provide quantitative summaries of the representative histological and immunostaining findings shown in b–k. We believe that presenting representative data first, followed by quantitative analysis, improves interpretability and follows a conventional figure organization. Therefore, we respectfully retained the original panel order.

2. In Sup Fig.5, the authors describe the isolation of SSC from parietal, occipital, mandible, and nasal bone, but not frontal.
→ We thank the reviewer for pointing this out. We inadvertently omitted the frontal bone from Supplementary Fig. 5. A representative FACS plot for the frontal bone has now been added to the revised figure.

3. In Fig. 2 the result of colony forming assay involves frontal cells. It is therefore confusing if the authors adopted the same method to isolate SSC through the investigation Later in Fig. 3 there are results with frontal cells. It would be important to make the text easy to understand.
→ We thank the reviewer for raising this point and agree that clarification is important. For the colony formation (CFU) assay in Fig. 2, only 1,000 cells per well were required, making it feasible to include frontal SSCs despite their relatively limited yield. In contrast, osteogenic and chondrogenic differentiation assays required substantially higher cell numbers (2,000 and 20,000 cells per well, respectively), which made inclusion of the frontal group technically challenging due to insufficient cell recovery. Therefore, frontal SSCs were excluded from these downstream functional assays. In Fig. 3, the analysis was based on FACS profiling, which requires substantially fewer isolated SSCs and can be performed from approximately 100,000 total bone cells. Thus, inclusion of the frontal group was feasible in this experiment. We have revised the text to clarify this rationale and improve readability.

Pg15 Ln291-293: Because FACS-based analysis requires relatively few cells, frontal bone was included in this experiment despite being excluded from downstream functional assays due to limited SSC yield in the previous section.

4. In Sup Fig. 8, are all y-axes the same, Counts/ Million cell?
→ We apologize for the inconsistent labeling and thank the reviewer for pointing this out. For Supplementary Fig. 8a–i, the y-axis represents counts per million cells. For Fig. 8j–n, the y-axis represents the ratio of BCSPs to SSCs (BCSP/SSC). The figure labels have now been revised to clearly reflect these differences.

5. In Fig.5a, GFP+/Sox9+ cells indicated by arrows do not show chondrogenic morphology. Since GFP+ cells were implanted, their characters may change, however, they do not look like chondrocytes compared with arrows in 9d. The results are after 4 weeks so it is hard to consider the cells are in differentiation process.

→ We thank the reviewer for this important comment. We agree that the $GFP^+/Sox9^+$ cells indicated in Fig. 5b do not display the classical morphology of mature chondrocytes. However, Sox9 expression is not restricted to morphologically mature chondrocytes and may also be detected in cartilage-associated progenitors or cells undergoing cartilage-related lineage programs in regenerative environments. Because the transplanted GFP^+ cells were implanted into a calvarial defect and assessed after 4 weeks, their morphology may differ from canonical cartilage tissue while still retaining Sox9 expression. To avoid overinterpretation, we have revised the text to indicate that $GFP^+/Sox9^+$ cells reflect activation of a chondrogenic program rather than definitive mature chondrocyte identity.

Pg24 Ln461-468: For femur-SSCs transplanted without BMP-2, both GFP^+/Osx^+ and $GFP^+/Sox9^+$ populations were detected (Fig. 5b-c), suggesting activation of both osteogenic and chondrogenic-associated lineage programs among transplanted cells. Although the $GFP^+/Sox9^+$ cells did not exhibit the classical morphology of mature chondrocytes, Sox9 expression may reflect cartilage-associated lineage activity in the regenerative environment. Pentachrome staining showed mainly bone formation in this experimental condition (Fig. 5b, top left), supporting the interpretation that $Sox9^+$ cells may represent a transient cartilage-associated component during ossification rather than mature cartilage tissue.

6. P.25, the authors describe the result with mandibular SSC and mention that mandibular bone occurs by intramembranous ossification, however, the parietal bone forms by intramembranous ossification. It seems that parietal SSC seems to show the same tendency.

→ We thank the reviewer for this insightful comment and agree that the parietal bone also develops through intramembranous ossification. We apologize for the lack of clarity in the original text. Our intention was not to imply that mandibular SSCs uniquely reflect intramembranous ossification. Rather, we aimed to highlight the distinct osteogenic phenotype observed following mandibular-SSC transplantation, characterized by ring-like ossification structures and a predominantly osteogenic, Sox9-low profile. Importantly, we agree that parietal SSCs, particularly following BMP-2 treatment, exhibited a similar tendency. We have revised the text to clarify this similarity and avoid overemphasizing ossification mode as a mandibular-specific feature.

Pg25 Ln483-488: Similar to BMP-2-treated parietal SSCs, mandibular-SSC implants exhibited predominantly osteogenic features with minimal $GFP^+/Sox9^+$ cells (Fig. 5h–i), consistent with a direct osteogenic differentiation trajectory. Upon BMP-2 treatment, the proportion of GFP^+/Osx^+ cells increased slightly (Fig. 5h–i), suggesting that mandibular SSCs primarily respond to BMP-2 by enhancing an already osteogenic program rather than initiating a cartilage-associated trajectory.

7. In Fig. 6, the position of Parietal and Femur circles should be considered. Although in vitro cartilage induction indicated that Femur has mot capacity, Parietal demonstrate more Sox9+GFP+ cells in Fig. 5. In addition, with BMP2 treatment, in vivo experiments exhibited suppression of cartilage formation overall. Therefore, it seems not right to see that most of arrows move to top right direction.

→ We thank the reviewer for this insightful suggestion. We have revised Fig. 6 to better reflect the lineage responses observed across both the in vitro and in vivo experiments. In the baseline panel, parietal SSCs were repositioned to reflect their relatively greater Sox9-associated/chondrogenic features observed in vivo.

In the BMP-2 response panel, arrow directions were revised to represent region-specific lineage changes. Nasal SSCs exhibited the most dramatic BMP-2-induced shift, characterized by enhanced osteogenic potential and reduced chondrogenic features in vivo; therefore, this response is depicted with a long arrow toward an osteogenic and reduced chondrogenic state. Parietal SSCs were adjusted to reflect enhanced osteogenesis with partial chondrogenic responsiveness, consistent with significant osteogenic induction in vivo and preserved chondrogenic responsiveness in vitro. Femur SSCs were depicted with a modest shift, reflecting limited in vivo changes despite responsiveness in vitro. Occipital SSCs were shown with an upper-right shift to reflect increased Sox9-associated/chondrogenic features together with maintained osteogenic potential following BMP-2 treatment. Mandibular SSCs were depicted as predominantly osteogenic, with minimal chondrogenic induction.

Pg35 Ln663-674: Nasal SSCs exhibit the most dramatic response, showing a marked shift toward osteogenesis accompanied by suppression of the chondrogenic program *in vivo* (**Fig. 5j,k**), reflected by the prominent upper-left trajectory. Occipital SSCs display mildly enhanced Sox9-associated/chondrogenic features *in vivo* while maintaining osteogenic potential, represented by an upper-right shift. Parietal SSCs exhibit enhanced osteogenesis *in vivo* together with preserved chondrogenic responsiveness *in vitro*, also reflected by an upper-right shift. Femur SSCs demonstrate limited lineage changes *in vivo* despite enhanced osteogenic and chondrogenic responses *in vitro*, resulting in a modest upper-right trajectory. Mandibular SSCs respond to BMP-2 predominantly through enhanced osteogenesis with minimal chondrogenic induction. This schematic highlights how intrinsic SSC diversity governs BMP-2 responsiveness and underscores the need for region-specific optimization of BMP-2–based bone regeneration strategies.

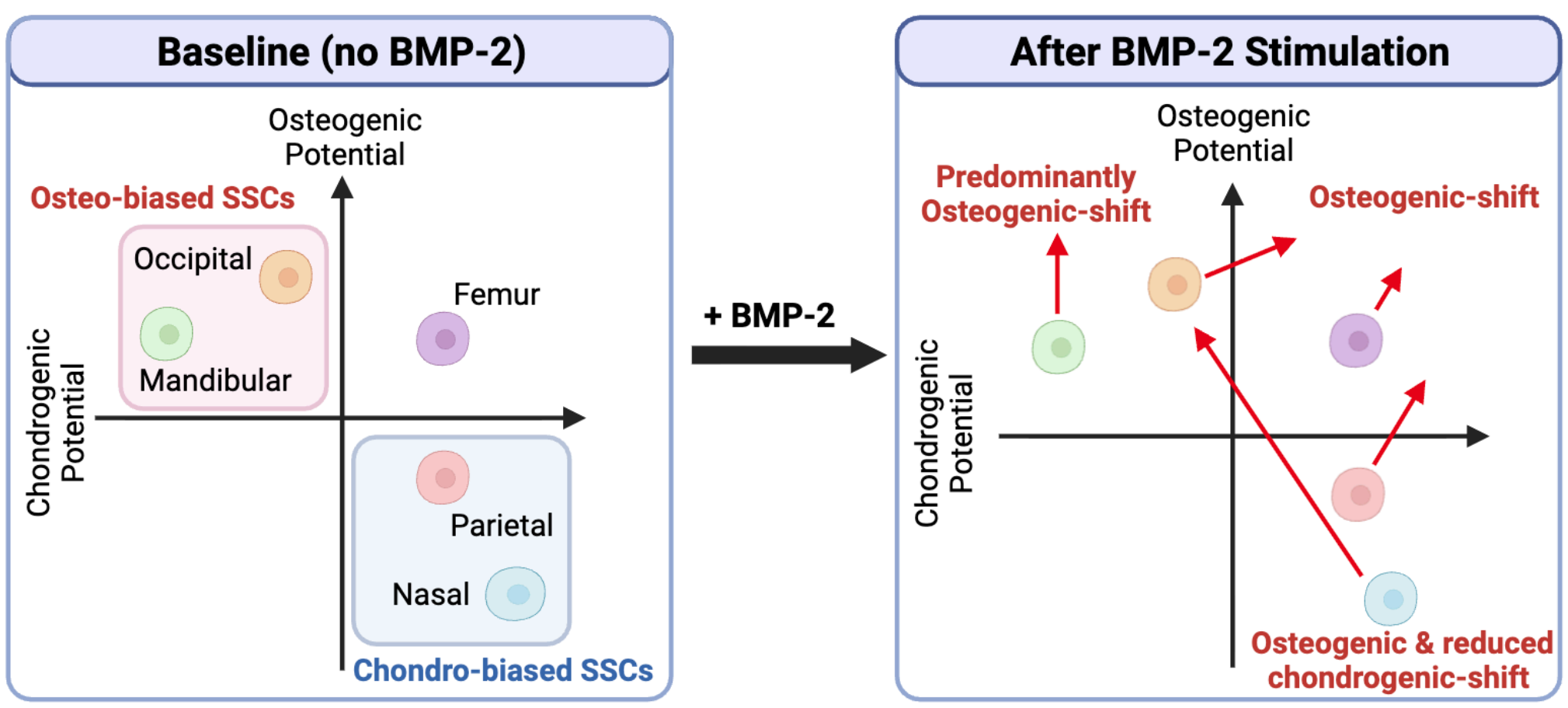

8. Line 584: The use of the parietal bone defect is understandable, however, “parietal regions may provide a more permissive environment that supports broader lineage potential” is the authors’ expectation without trying other bone defects.

→ We thank the reviewer for this comment. We agree that the contribution of regional niche environments to SSC behavior was not directly tested in the present study. Our intention was not to conclude that parietal regions definitively provide a more permissive environment, but rather to propose a potential explanation for the observed regional differences in SSC lineage behavior. We have revised the text to clarify that these niche-dependent influences remain speculative and warrant future investigation.

Pg32 Ln587-589: Conversely, differences in the local cranial niche may contribute to broader

lineage potential in other regions, although this possibility was not directly examined in the present study.

9. Line 594: Is high clonality generally associated with the differentiation bias of the isolated SSC or high proliferation?
→We thank the reviewer for this important comment. High clonality is generally considered to reflect colony-forming and self-renewal capacity and is often associated with greater stemness or multipotency. However, it does not necessarily predict differentiation fate. In our study, nasal SSCs exhibited high clonal frequency but predominantly differentiated into cartilage under basal conditions, indicating a strong chondrogenic bias despite robust clonogenic potential. This suggests that clonogenic capacity and lineage bias can be independently regulated. We have clarified this point in the revised manuscript.

Pg32Ln599-602: Notably, despite exhibiting high clonal frequency—a feature generally associated with proliferative and self-renewal potential—nasal SSCs predominantly formed cartilage under basal conditions, indicating that proliferative capacity and lineage fate are not necessarily coupled.

10. Line 617: How does airflow produce mechanical stimulation?

→ We thank the reviewer for this comment. We agree that the mechanism by which airflow may influence SSC maintenance was insufficiently explained. Our intention was to speculate that repeated airflow through the nasal cavity could contribute to local biomechanical or physiological stimulation of the surrounding tissues, analogous to how mastication provides mechanical loading in the mandible. However, this was not directly examined in the present study. We have revised the text to clarify this point and temper the interpretation.

Pg33Ln623-625: possibly reflecting region-specific physiological stimuli, including mastication in the mandible and local biomechanical or physiological stimulation associated with airflow in the nasal cavity, although this possibility remains speculative.

Response to Reviewer 3

Line: 344-345: Mouse weaning does not occur at P14. The authors refer the paper that describe "In commercial breeding facilities, weaning occurs usually in the third week after birth. If dams are checked for parturition twice weekly—which according to personal communication with various commercial breeders seems to be common practice—newly weaned animals may range in age from 17 to 25 days of age.". The data for BCSP/SSC ratio in mandible must have other reasons. At p14, the pups are still drinking milk.

→ We thank Reviewer 3 for this insightful comment. We agree that mouse pups remain primarily dependent on maternal milk at P14, although they begin transitioning toward solid food during this developmental period. We also note that P14 falls within the period of rapid postnatal mandibular growth, with anterior–posterior growth occurring from P7–P28 and vertical growth in the posterior ramus increasing from P7–P21 before slowing thereafter (Ref. 34). Accordingly, we have softened our interpretation, removed the previous reference linking P14 to the initiation of solid food consumption, and instead cite the mandibular growth study. We now emphasize that the underlying mechanism remains unclear and present mandibular maturation as a possible explanation for the transient increase in the BCSP/SSC ratio.

Line 275-280: This transient increase occurs during the period of rapid postnatal mandibular growth (P7–P21), when mouse pups begin transitioning toward solid food while continuing to nurse[34]. Although the underlying mechanism remains unclear, the elevated BCSP/SSC ratio may reflect the developmental demands associated with mandibular maturation. Beyond P14, the BCSP/SSC ratio subsequently decreases, suggesting progression beyond this transient developmental stage.
